# Single-cell RNA sequencing integrated with bulk RNA sequencing analysis identifies a tumor immune microenvironment-related lncRNA signature in lung adenocarcinoma

**DOI:** 10.1186/s12915-024-01866-5

**Published:** 2024-03-22

**Authors:** Yuqing Ren, Ruhao Wu, Chunwei Li, Long Liu, Lifeng Li, Siyuan Weng, Hui Xu, Zhe Xing, Yuyuan Zhang, Libo Wang, Zaoqu Liu, Xinwei Han

**Affiliations:** 1 Department of Interventional Radiology, The First Affiliated Hospital of Zhengzhou University, Zhengzhou, 450052 Henan China; 2 Department of Respiratory and Critical Care Medicine, The First Affiliated Hospital of Zhengzhou University, Zhengzhou, 450052 Henan China; 3 Internet Medical and System Applications of National Engineering Laboratory, The First Affiliated Hospital of Zhengzhou University, Zhengzhou, 450052 Henan China; 4 Department of Pharmacy, The First Affiliated Hospital of Zhengzhou University, Zhengzhou, 450052 Henan China; 5 Department of Hepatobiliary and Pancreatic Surgery, The First Affiliated Hospital of Xi’an Jiaotong University, Xi’an, 710061 Shanxi China; 6 Cancer Center, The First Affiliated Hospital of Zhengzhou University, Zhengzhou, 450052 Henan China; 7 Department of Neurosurgery, The Fifth Affiliated Hospital of Zhengzhou University, Zhengzhou, 450000 Henan China; 8 Department of Pancreatic Cancer, Tianjin Medical University Cancer Institute and Hospital, Tianjin, 300060 China; 9 Institute of Basic Medical Sciences, Chinese Academy of Medical Sciences and Peking Union Medical College, Beijing, 100730 China

**Keywords:** Lung adenocarcinoma, Tumor immune microenvironment, Single-cell RNA sequencing, Immunotherapy

## Abstract

**Background:**

Recently, long non-coding RNAs (lncRNAs) have been demonstrated as essential roles in tumor immune microenvironments (TIME). Nevertheless, researches on the clinical significance of TIME-related lncRNAs are limited in lung adenocarcinoma (LUAD).

**Methods:**

Single-cell RNA sequencing and bulk RNA sequencing data are integrated to identify TIME-related lncRNAs. A total of 1368 LUAD patients are enrolled from 6 independent datasets. An integrative machine learning framework is introduced to develop a TIME-related lncRNA signature (TRLS).

**Results:**

This study identified TIME-related lncRNAs from integrated analysis of single‑cell and bulk RNA sequencing data. According to these lncRNAs, a TIME-related lncRNA signature was developed and validated from an integrative procedure in six independent cohorts. TRLS exhibited a robust and reliable performance in predicting overall survival. Superior prediction performance barged TRLS to the forefront from comparison with general clinical features, molecular characters, and published signatures. Moreover, patients with low TRLS displayed abundant immune cell infiltration and active lipid metabolism, while patients with high TRLS harbored significant genomic alterations, high PD-L1 expression, and elevated DNA damage repair (DDR) relevance. Notably, subclass mapping analysis of nine immunotherapeutic cohorts demonstrated that patients with high TRLS were more sensitive to immunotherapy.

**Conclusions:**

This study developed a promising tool based on TIME-related lncRNAs, which might contribute to tailored treatment and prognosis management of LUAD patients.

**Supplementary Information:**

The online version contains supplementary material available at 10.1186/s12915-024-01866-5.

## Background

As the second most prevalent cancer, lung cancer remains the major cause of cancer-related mortality worldwide [1]. The majority of lung cancers are occupied by non-small cell lung cancer (NSCLC), composing lung adenocarcinoma (LUAD) and lung squamous cell carcinoma (LUSC) [2]. LUAD, the most predominant pathological subtype, occupies more than 40% of lung cancer cases. The emergence and development of molecular-targeted therapy and immune checkpoint blockade therapy provide LUAD patients with more therapeutic options and clinical benefits [3]. However, LUAD patients have poor median overall survival (OS), and the 5-year survival rates were also disappointing [4]. It is inevitable and urgent to distinguish and examine LUAD patients with poor prognoses to offer personalized treatment.

Consisting predominantly of different immune cell populations, tumor immune microenvironment (TIME) exacts a profound impact on the clinical outcomes and immunotherapeutic response [5–7]. Despite numerous TIME-related studies that have been carried out in bulk RNA-seq data, limited resolution and lack of cellular heterogeneity remain hindrances and obstacles to further exploration of TIME. Recently, single-cell RNA-seq (scRNA-seq) technology has flourished as a powerful platform for accurately deciphering TIME characteristics at specific single-cell resolutions [8]. For instance, He et al. portrayed the TIME landscape of TKI-resistant LUAD patients by scRNA-seq and uncovered its connection with circadian rhythm disorder [9]. Based on scRNA-seq analysis, Di et al. revealed the TIME heterogeneity of early-stage LUAD patients harboring EGFR mutations [10]. Consequently, scRNA-seq might be a promising booster to dissect TIME for LUAD patients. Based on lncRNA-miRNA or lncRNA-mRNA interactions, lncRNA could influence tumor progression by regulating essential gene expressions, such as oncogenes and tumor suppressor genes [11]. Accumulating evidences have revealed that this regulation could reallocate the proportion of composed cells and affect chemokine expression in tumor initiation and progression, to the point that TIME was remodeled [12, 13]. Based on these findings, a couple of studies were carried out to explain the crucial impact of lncRNA on prognosis prediction and immunotherapy [14, 15]. Nevertheless, the clinical outcome-related research of lncRNAs remains largely deficient in LUAD.

In this study, we systematically integrated both scRNA-seq and bulk RNA-seq data to identify TIME-related lncRNAs. To further explore the clinical significance of TIME-related lncRNAs, we applied ten popular machine learning algorithms to generate an optimal TIME-related lncRNA signature (TRLS). TRLS displayed stable and robust performance for predicting prognosis in six independent multi-center cohorts. Subsequent comparison with clinical characteristics and published signatures indicated that TRLS possessed a superior accuracy. We also evaluated TRLS thoroughly in terms of potential biological mechanisms, immune landscape, genomic alterations, and immunotherapy responses underlying TRLS. Taken together, our TRLS model is a promising tool for improving prognosis and precision treatment for LUAD patients.

## Methods

### Data collection and processing

#### Single-cell RNA sequencing data

The 10X single-cell transcriptome data from GSE171145 [16] (including 40,799 cells, https://www.ncbi.nlm.nih.gov/geo/query/acc.cgi?acc=GSE171145) were downloaded from the Gene Expression Omnibus (GEO) (http://www.ncbi.nlm.nih.gov/geo/) database.

#### LUAD patient cohorts

Transcriptome and clinical data of LUAD patients were acquired from the Cancer Genome Atlas (TCGA) and GEO databases. Ultimately, we incorporated six independent cohorts with abundant profiles and complete prognosis information, including TCGA-LUAD (*n* = 497, https://www.cancer.gov/tcga), GSE72094 (*n* = 398, https://www.ncbi.nlm.nih.gov/geo/query/acc.cgi?acc=GSE72094) [17], GSE50081 (*n* = 127, https://www.ncbi.nlm.nih.gov/geo/query/acc.cgi?acc=GSE50081) [18], GSE31210 (*n* = 226, https://www.ncbi.nlm.nih.gov/geo/query/acc.cgi?acc=GSE31210) [19], GSE30219 (*n* = 83, https://www.ncbi.nlm.nih.gov/geo/query/acc.cgi?acc=GSE30219) [20], and GSE3141 (*n* = 37, https://www.ncbi.nlm.nih.gov/geo/query/acc.cgi?acc=GSE3141) [21].

#### Immunotherapeutic cohorts

Patients from nine immunotherapeutic cohorts, including GSE35640 (*n* = 65, https://www.ncbi.nlm.nih.gov/geo/query/acc.cgi?acc=GSE35640) [22], GSE78220 (*n* = 28, https://www.ncbi.nlm.nih.gov/geo/query/acc.cgi?acc=GSE78220) [23], GSE91061 (*n* = 109, https://www.ncbi.nlm.nih.gov/geo/query/acc.cgi?acc=GSE91061) [24], GSE93157 (*n* = 65, https://www.ncbi.nlm.nih.gov/geo/query/acc.cgi?acc=GSE93157) [25], GSE100797 (*n* = 25, https://www.ncbi.nlm.nih.gov/geo/query/acc.cgi?acc=GSE100797) [26], GSE115821 (*n* = 37, https://www.ncbi.nlm.nih.gov/geo/query/acc.cgi?acc=GSE115821) [27], GSE126044 (*n* = 16, https://www.ncbi.nlm.nih.gov/geo/query/acc.cgi?acc=GSE126044) [28], GSE136961 (*n* = 21, https://www.ncbi.nlm.nih.gov/geo/query/acc.cgi?acc=GSE136961) [29], and GSE145996 (*n* = 14, https://www.ncbi.nlm.nih.gov/geo/query/acc.cgi?acc=GSE145996) [30], were enrolled to estimate the immunotherapeutic response. Baseline data of LUAD cohorts and immunotherapeutic cohorts were presented in Additional file 7.

#### Multi-omics data for TCGA-LUAD

Somatic mutation profile and segmented copy number variation (CNV) of TCGA-LUAD patients were downloaded from the TCGA database. Moreover, mRNA stemness indices and tumor mutation burden (TMB) were respectively calculated to evaluate the correlation with TRLS. In the cancer-immune group atlas (TCIA, https://tcia.at/home), we collected the neoantigen data of LUAD patients in the TCGA-LUAD cohort [31].

#### Data processing

Quality control of single-cell RNA sequencing data was executed with the following criteria: > 200 genes/cell, < 3000 genes/cell, > 3 cells/gene, and < 20% mitochondrion genes. The batch effect of 9 samples was eliminated using IntegrateData of Seurat packages [32]. The top 30 principal component analysis (PCA) components were determined after the identification of the top 2000 highly variable genes. Then, the first 15 significant PCs determined by jackstraw analysis were incorporated to conduct cell clustering using the findCluster method (resolution = 0.2), which implements the Louvain network-based clustering algorithm. Subsequently, Uniform Manifold Approximation and Projection (UMAP) analysis was applied for further dimensional reduction and clustering visualization [33]. We further performed the “FindAllMarkers” function to identify TIME-related genes, which were defined as genes with |log2 (fold change)|> 1 and adjusted *P*-value < 0.05 for TIME cells. After converting RNA-seq count data to transcripts per kilobase million (TPM) and further log-2 transferring, microarray data harbors more comparability was acquired. The robust multi-array average (RMA) algorithm was implemented to normalize microarray data in the affy package. Across all cohorts, the expression of each gene was converted into a *Z*-score value before model construction. For the TCGA-LUAD dataset and GEO datasets, each cohort was regarded as an independent cohort, and no batch effect elimination was necessary. The maftools package was applied for processing and visualizing the genomic alteration data [26]. The burden of copy number alteration, including amplification and deletion, was measured at both the focal and arm levels using CNV data from the GISTIC 2.0 pipeline. As previously reported, we estimated the percentage of genome alteration (FGA), fraction of genomic gain (FGG), and fraction of genome loss (FGL) [19].

### Consensus clustering

In the TCGA-LUAD cohort, the ConsensusClusterPlus package was applied for immune subtype discovery based on TIME-related gene expression via the following parameters: number of iterations = 100, possible cluster numbers = 2–9, cluster algorithm = *K*-means, and Euclidean distance [34]. Afterward, related indicators were employed to determine the optimal number of clusters, including the proportion of ambiguous clustering (PAC) score, cumulative distribution function (CDF) curve, and the consensus score matrix [35]. Subsequently, seven algorithms were conducted to distinguish and verify the immune infiltration between the two identified clusters, including CIBERSORT, EPIC, ESTIMATE, MCP-counter, quanTIseq, TIMER, and xCell [36, 37].

### Weighted correlation network analysis (WGCNA)

The construction of TCGA-LUAD co-expression lncRNA networks was realized by employing the WGCNA package. After determining a suitable soft threshold *β*, a topological overlap matrix (TOM) was created by transforming the weighted adjacency matrix to generate clustered modules. Then, the correlation between modules and two clusters was calculated. To recognize lncRNAs significantly correlated with two clusters, lncRNAs in modules with a correlation coefficient > 0.3 or <  − 0.3 were selected for further study.

### Integrative machine learning algorithms to generate signatures

To further exploit the prognostic significance of TIME-related lncRNA, we leveraged 10 machine learning algorithms, composing CoxBoost, elastic network (Enet), generalized boosted regression modeling (GBM), LASSO, partial least squares regression for Cox (plsRcox), random survival forest (RSF), Ridge, stepwise Cox, supervised principal components (SuperPC), and survival support vector machine (survival-SVM). Subsequently, these algorithms were integrated into 96 combinations to develop a robust TIME-related lncRNA signature [38, 39]. The final signature was generated following the pipeline from our previous studies [38, 39]: (i) Identified prognostic associated lncRNAs through Cox regression in 6 LUAD cohorts. A specific lncRNA was considered prognostic significant only if *P* < 0.05 of Cox regression in ≥ 5 cohorts; (ii) Used 96 algorithm combinations to apply prognostic lncRNAs to fit prediction models through tenfold cross-validation in TCGA-LUAD cohort; (iii) Tested all models in 5 verification cohorts (GSE72904, GSE50081, GSE31210, GSE30219, and GSE3141); (iv) For each model, calculated the Harrell concordance index (C-index) of all cohorts; (v) Across all cohorts, the model with the highest mean C-index was regarded as the optimal and excellent one.

### Cell culture and transfection

The human LUAD cell lines A549 and Calu3 were maintained in RMPI 1640 medium (Hyclone, USA) supplemented with 10% fetal bovine serum (FBS, Hyclone, USA). In a 5% CO_2_ humidified incubator, we maintained LUAD cell lines A549 and Calu3 in RMPI 1640 medium (Hyclone, USA) containing 10% fetal bovine serum (FBS), 100 units/ml of penicillin, and 100 g/ml of streptomycin at 37 °C. According to the manufacturer’s protocol, the siRNAs of Lnc00857 and negative control siRNA were transfected using Lipofectamine 3000 (Life Technologies, USA). The sequence of siLnc00857 was as follows: sense GAGACUGAUUUGAGUGAUA (dT)(dT), antisense UAUCACUCAAAUCAGUCUC (dT)(dT). Quantitative real-time PCR was performed to validate the knockdown efficiency and the following primer sequences were used: forward primer: (GCATGAAAGAATTGGCCGCA), reverse primer: (CCCAGGATGCCTGTTGTTCA).

### Cell viability and EdU incorporation assay

The cell viability of A549 and Calu3 cells was assessed using the Cell Counting Kit-8 (CCK-8; Dojindo, Japan) assay. LUAD cells were seeded in 96-well plates and cultured under proper conditions 48 h post-transfection. After culturing for 24 h, 48 h, 72 h, and 96 h, each well was incubated for 2 h with 10 mL CCK-8 reagent. At 450 nm wavelength, the absorbance was measured by a multiscan spectrum (BioTek, Winooski, VT, USA) for cells in each well.

After seeding Lnc00857 knockdown or control LUAD cells in 96-well plates for 24 h, they were incubated for 2 h with 50 μM EdU (Beyotime, Shanghai, China) at a cell incubator. In accordance with the instructions, the following steps were executed after being fixed with 4% paraformaldehyde. EdU-positive cells (red fluorescence) and Hoechst-positive cells (blue fluorescence) were captured with a microscope (Olympus, Tokyo, Japan).

### Wounding healing and Transwell assay

Transfected A549 and Calu3 cells were inoculated into 6-well culture plates. A sterile 200-L pipette tip was used to scratch cells in the serum-free medium when cells grew into 100% confluence. At 0 and 48 h after scratching, the wound width was imaged.

Transwell assays were conducted through a Transwell system, which has pore sizes of 8.0 mm and a 24-well insert. For invasion assays, Matrigel (BD Biosciences) was plated onto the wells. The upper chambers were filled with LUAD cells, and the lower chambers with medium (600 μL per well) were supplemented with 10% FBS. Afterward, a migration assay or invasion assay was conducted following incubation in standard culture conditions for 24 h or 48 h, respectively. After incubation, a 15-min fixation in 4% paraformaldehyde was followed by a 5-min staining in 0.1% crystal violet.

### Immune infiltration and tumor immunogenicity assessment

LUAD samples were separated into high TRLS and low TRLS groups after the optimal cutoff value was determined using the survminer package. For the two groups, ESTIMATE and ssGSEA algorithms were implemented to evaluate the immune infiltration. Immunogenicity-related phenotypes were collected from previous research to understand the different immune escape processes between the two groups [40].

### Exploring functional differences

After analyzing the gene expression differences between the two groups, we arranged the genes in log2-transformed fold change (log2FC) decreasing order. Subsequently, gene sets from Gene Ontology (GO), Kyoto Encyclopedia of Genes and Genomes (KEGG), and Hallmark were introduced to perform gene set enrichment analysis (GSEA) using the clusterProfiler package.

### Immunotherapeutic response prediction

The cancer-immune cycle and immunotherapy signatures were retrieved from previous studies [41, 42]. Besides, immune checkpoint gene expression, PD-L1 protein expression, and tumor immune dysfunction and exclusion (TIDE) score [43] were evaluated to reflect the performance of TRLS in predicting immunotherapy. Meanwhile, nine immunotherapy cohorts were recruited to further validate the immunotherapeutic response assessment through an unsupervised subclass mapping (SubMap) algorithm [44].

### Statistical analysis

All data processing, plotting, and statistical analysis were conducted in R (version 4.1.2). The CompareC package was applied to conduct C-indices comparisons of different variables. To compare the quantitative variables between the two groups, the Wilcoxon rank-sum or *T* test was applied. The survival package provided a platform to perform Cox regression and Kaplan–Meier analyses. Furthermore, correlation analysis of continuous variables was accomplished through the Spearman tests. For all statistical tests, a two-sided *P* < 0.05 was deemed statistically significant.

### Ethics section

Not applicable. This study did not include any human or animal experiments. The datasets presented in this study can be found in online repositories. The names of the repository/repositories and accession number(s) can be found in the article. The authors are accountable for all aspects of the work in ensuring that questions related to the accuracy or integrity of any part of the work are appropriately investigated and resolved.

## Results

### scRNA‑seq analysis revealed immune landscape and TIME-related genes in LUAD

**Fig. 1:**
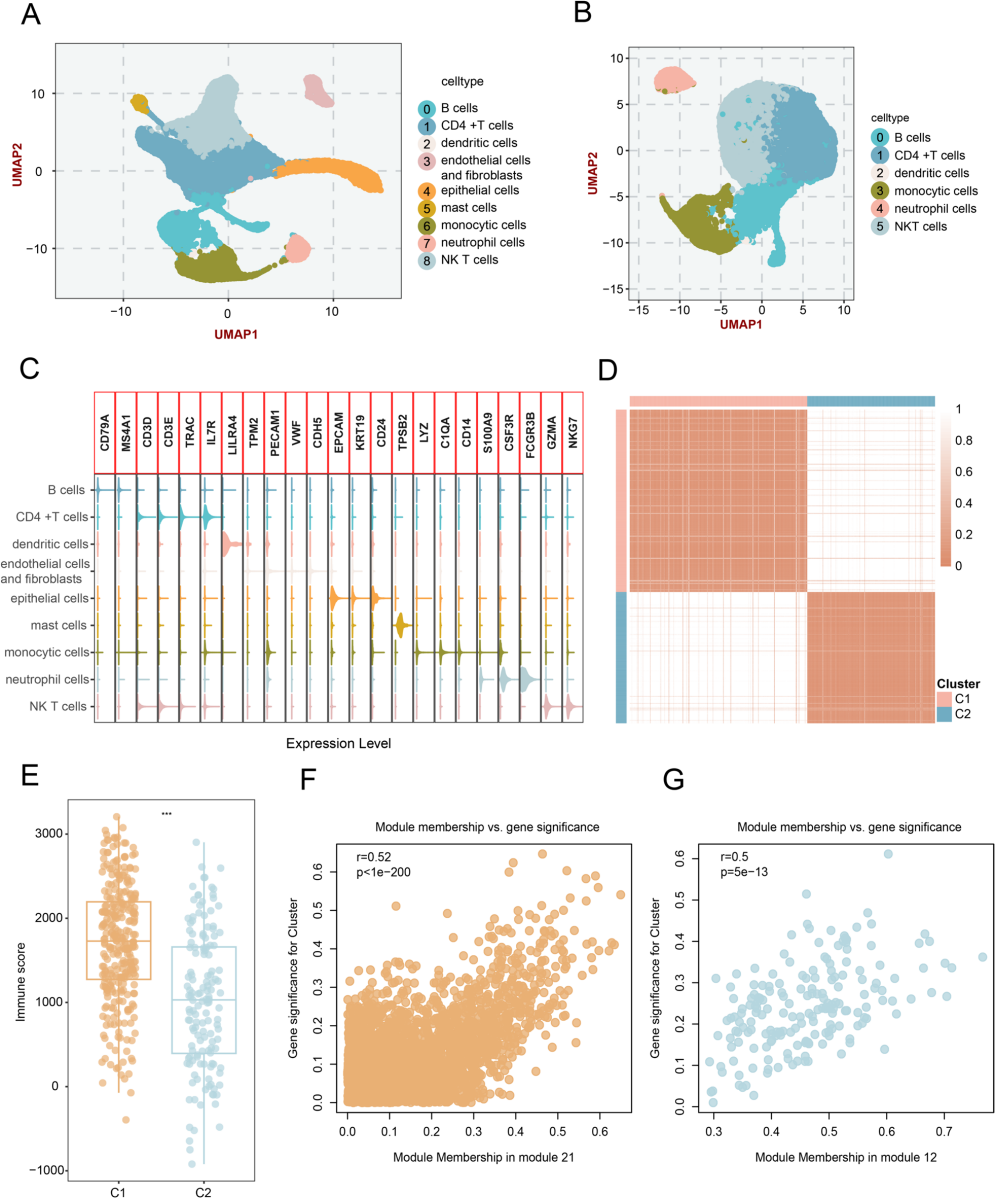
Identification of TIME-related lncRNAs. **A** Cell landscapes of 40,799 cells from 9 types (B cells, CD4 + T cells, dendritic cells, endothelial cells/fibroblasts, epithelial cells, mast cells, monocytic cells, neutrophil cells, NKT cells). **B** UMAP plot of TIME-related cells. **C** Expression levels of representative cell type markers on the violin plot. **D** The consensus score matrix of TCGA samples when *k* = 2. **E** Immune score for two clusters inferred via the ESTIMATE algorithm. **F**, **G** The correlation between module membership and gene significance in module 21 and module 12

A total of 40,799 cells from nine LUAD samples were performed via 10X scRNA-seq [16]. Data quality control ultimately retained 30,011 cells (Additional file 1). Subsequently, UMAP analysis revealed 11 clusters, which were further annotated into the following cell types: immune cells (B cells, CD4 + T cells, dendritic cells, monocytic cells, neutrophil cells, and NKT cells), endothelial cells, fibroblasts, epithelial cells, and mast cells (Fig. 1A, B). To characterize the TIME in LUAD bulk tissues, we extracted immune cells to identify 1088 TIME-related genes (Fig. 1C and Additional file 8). Univariate Cox regression revealed a total of 330 genes with prognostic significance (*P* < 0.05) in TCGA-LUAD, which were selected for subsequent investigation (Additional file 9).

### Discovery of immune subtypes and TIME-related lncRNAs

Using expression profiles of 330 TIME-related genes, consensus cluster analysis was conducted in TCGA-LUAD bulk RNA-seq data (*n* = 497). According to the PAC score, CDF curve, and consensus score matrix (Fig. 1D and Additional file 2A, B), two immune subtypes were regarded as the optimal selection [39]. Subsequently, seven different algorithms were applied for characterizing the immune environment of LUAD bulk samples and revealed that C1, an “immune-hot” subtype, was endowed with a higher immune score and abundant infiltration of multifarious immune cells, such as macrophages, T cells, and dendritic cells (Fig. 1E and Additional file 3). Conversely, C2 was featured by sparse immune constituents and termed the “immune-cold” subtype (Additional file 2C). To further identify potential lncRNA modulators of immune infiltration patterns, we employed the WGCNA algorithm [45] to decipher 21 lncRNA modules, in which 5 modules with a Pearson correlation > 0.3 or <  − 0.3 (Fig. 1F, G and Additional file 2D-G). Ultimately, a total of 850 lncRNAs from these modules were considered TIME-related lncRNAs for subsequent analysis (Additional file 10).

### Development of TRLS from the integrative machine-learning framework and internal and external validation of its prognostic predicting value

**Fig. 2:**
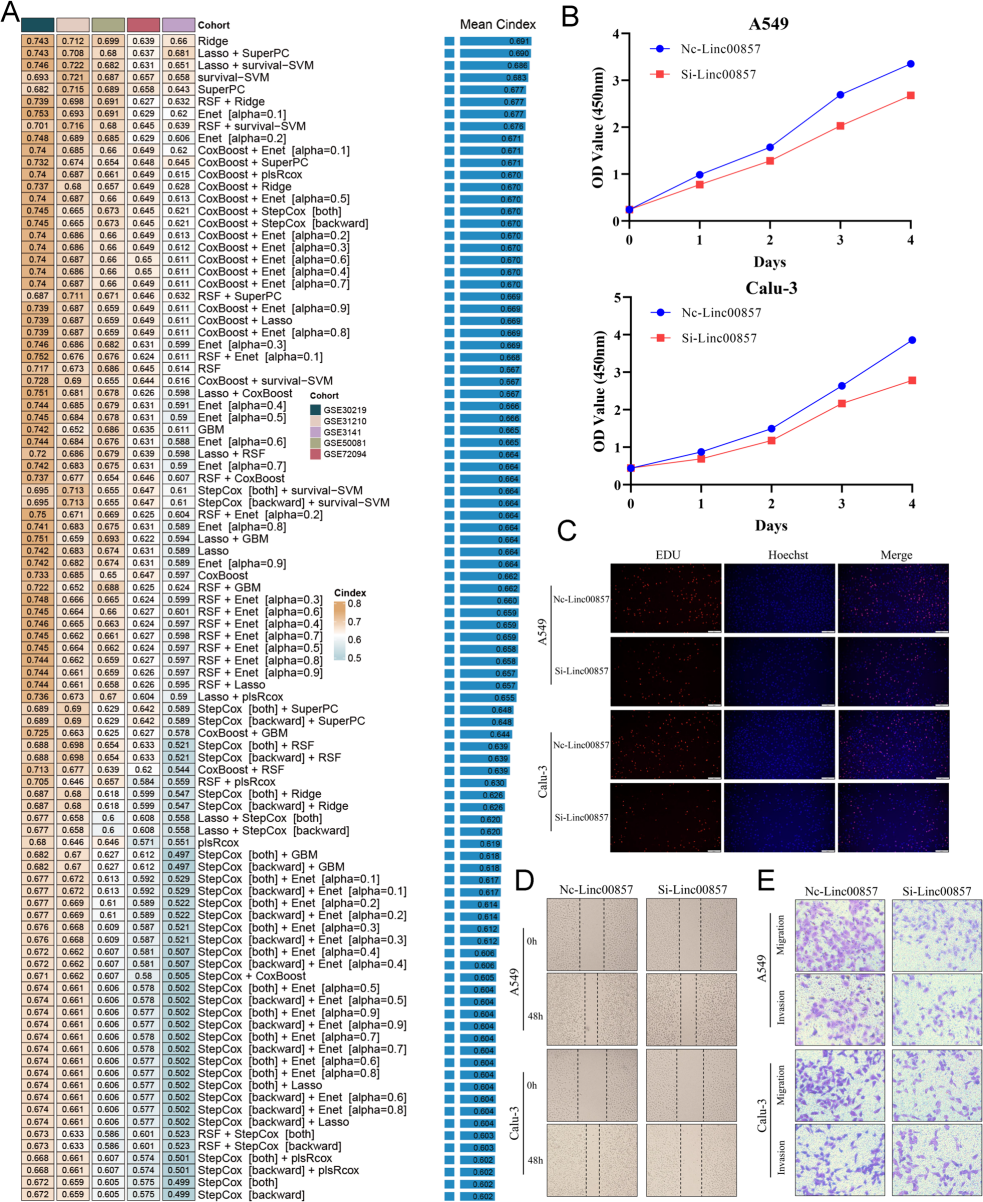
Integrative construction and validation of a consensus TRLS. **A** C-indices of 96 kinds of machine learning-based prediction models in 5 validation cohorts. **B** The cell viability was assessed through CCK-8 proliferation assay in A549 and Calu3 cell lines. **C** EdU incorporation assay of A549 and Calu3 cell lines apply to detect the cell proliferation ability. **D** Wound healing assay was performed to evaluate the cell migration rate. **E** Transwell assay was applied to detect the migration and invasion ability of A549 and Calu3 cells

To further explore the prognostic significance of 850 TIME-related lncRNA, we introduced an integrative machine learning framework as previously reported [38, 39]. Initially, univariate Cox analysis demonstrated that 33 lncRNAs were prognostic significant with *P* < 0.05 of Cox regression in ≥ 5 cohorts (Additional file 4A and Additional file 11). Afterward, these 33 lncRNAs were incorporated into our machine learning-based integrative framework to generate a consensus TRLS. In the TCGA-LUAD training cohort, we fitted 96 types of prediction models based on tenfold cross-validation. Due to the latent overfitting in the training dataset might dramatically overstate model performance, we utilized the mean C-index of 5 validation cohorts (excluded TCGA-LUAD training cohort) to evaluate the predictive ability of all models, which also displayed the real generalization ability of each model [38, 39]. As illustrated in Fig. 2A, with the highest mean C-index (0.691), the Ridge model was regarded as the optimal one. Following that (TRLS = $\sum_{i=1}^{33}{\text{gene}}(i)\times {\text{coef}}(i)$, where gene(*i*) and coef(*i*) represented the gene expression level and the coefficient index, respectively), each patient’s risk score was computed by the supplementary formula (Additional files 12 and 13). Kaplan–Meier analysis demonstrated that the high TRLS group showed a worse overall survival (OS) than the low TRLS group (TCGA-LUAD: *P* < 0.0001; GSE72094: *P* < 0.0001; GSE50081: *P* < 0.0001; GSE31210: *P* < 0.0001; GSE30219: *P* < 0.0001; GSE3141: *P* = 0.0004; Additional file 4B). Meanwhile, TRLS preserved statistical significance when exploitable clinical features were adjusted in multivariate Cox regression (all *P* < 0.05), which indicated that TRLS was an independent risk factor for OS (Additional file 14). We further selected Lnc00857 for knockdown and performed cell function experiments to decode the role of TRLS in LUAD cell function regulation. In PCR assay, Lnc00857 was successfully knocked down in A549 and Calu3 cells after siLnc00857 transfection (Additional file 4C). The CCK-8 and EdU assay were conducted to provide cell viability (Fig. 2B) and proliferation (Fig. 2C) evaluation. In the siLnc00857 group, decreased cell viability and proliferation were revealed compared to the NC group due to depressed Lnc00857 expression. Besides, the wound healing and Transwell assay were carried out, which exhibited migration and invasion ability alteration of LUAD cells. In two LUAD cell lines, lower migrant capacity was revealed from the wound healing assay after Lnc00857 knockdown (Fig. 2D). Transwell assay indicated that Lnc00857 silencing inhibited migration and invasion (Fig. 2E). These results demonstrated that Lnc00857 was a lncRNA with cancer-promoting effects, including migration, invasion, and proliferation.

### Robust performance of TRLS

**Fig. 3:**
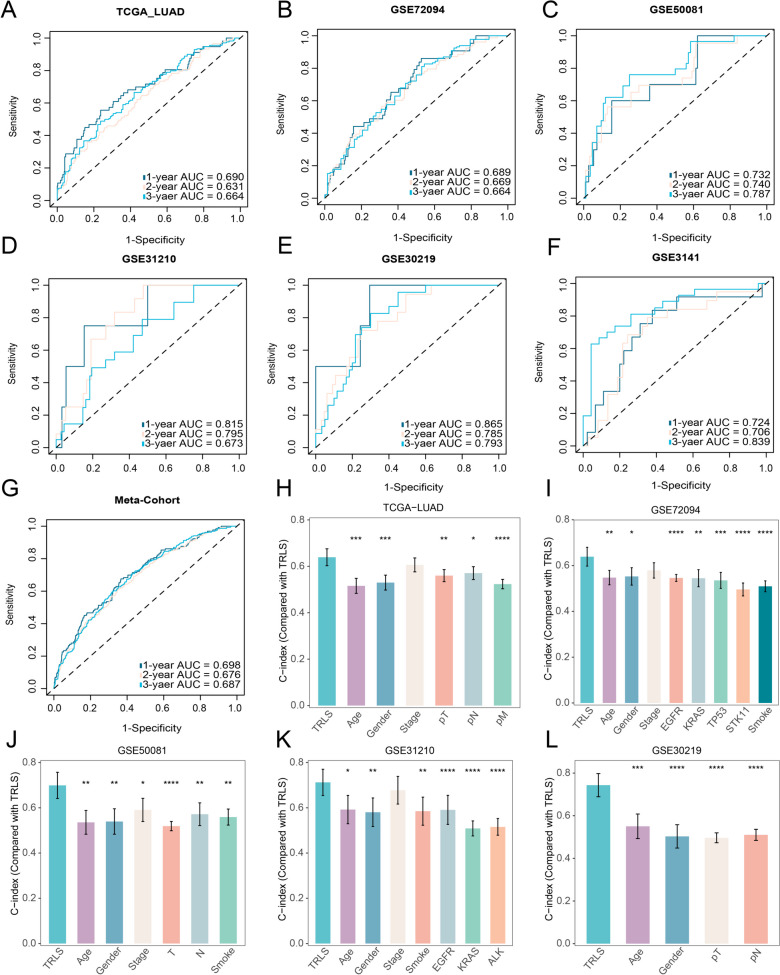
The performance assessment of TRLS. **A**–**G** Time-dependent ROC analysis for predicting OS at 1, 2, and 3 years across all cohorts. **H**–**L** The prognostic prediction performance of TRLS was compared with general clinical and molecular characters across TCGA-LUAD, GSE72094, GSE50081, GSE31210, and GSE30219. *P* values are shown as *P < 0.05, **P < 0.01, and ***P < 0.001

Discrimination of TRLS was assessed using time-dependent ROC curves in six independent cohorts, with 1/2/3 years AUCs of 0.690/0.631/0.664 in TCGA-LUAD, 0.689/0.669/0.680 in GSE72094, 0.732/0.740/0.787 in GSE50081, 0.815/0.795/0.673 in GSE31210, 0.865/0.785/0.793 in GSE30219, 0.724/0.706/0.839 in GSE3141, and 0.698/0.676/0.687 in Meta-Cohort (Fig. 3A–G). In clinical practice, classical clinicopathological parameters (e.g., AJCC/TNM stage) and novel molecular alterations (e.g., EGFR/KRAS mutations) have been demonstrated to be effective prognostic indicators for LUAD patients. Hence, the prediction performance of TRLS was compared with general clinical features, including age, gender, TNM stage, smoking, and mutation of EGFR, KRAS, TP53, or ALK to illustrate the value of TRLS in clinical management. Relatively speaking, in five cohorts with complete clinical information, TRLS possessed a superior performance in predicting prognosis compared with these clinicopathological traits (Fig. 3H–L). Therefore, our TRLS model displayed excellent stability and accuracy for prognosis assessment, which might serve as a promising tool for identifying “high-risk” patients in clinical settings.

### Comparison between TRLS and 79 published signatures

**Fig. 4:**
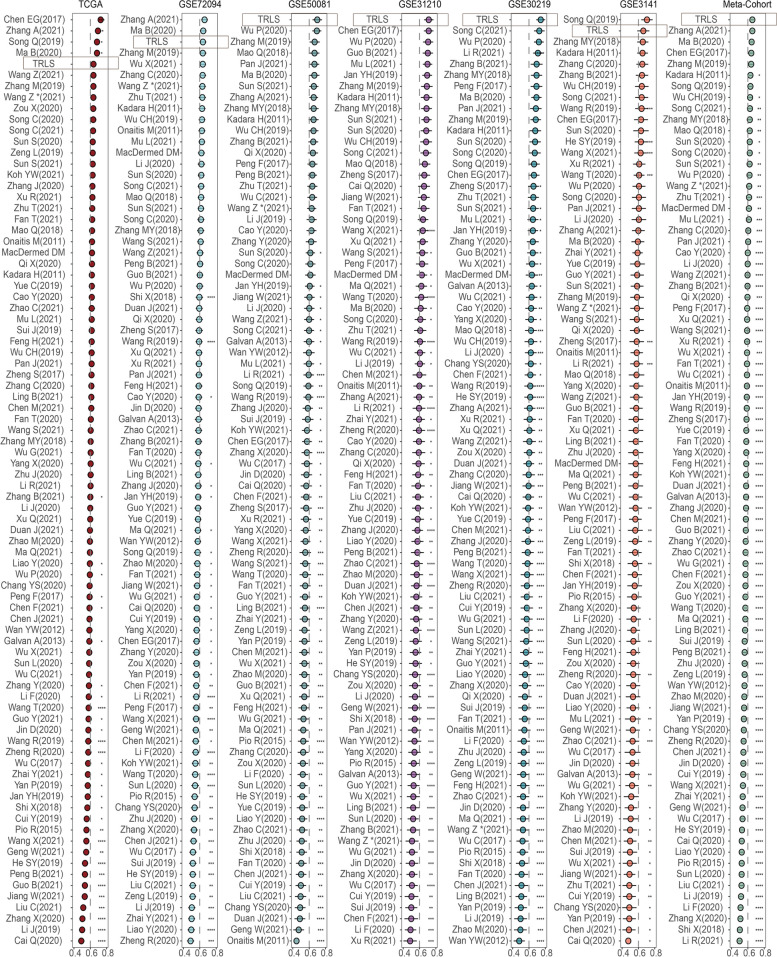
Comparisons between TRLS and signatures based on gene expression profile. Univariate Cox regression analysis of TRLS and 79 published signatures in TCGA-LUAD, GSE72094, GSE50081, GSE31210, GSE30219, GSE3141, and Meta-Cohort. *P* values are shown as *P < 0.05, **P < 0.01, and ***P < 0.001

With advances in next-generation sequencing (NGS) technology and the vigorous development of machine learning algorithms, an increasing number of researchers prefer to apply machine learning algorithms to develop survival prediction signatures [46, 47]. Based on distinct biological characteristics and molecular types, copious developed signatures have displayed prognostic value in LUAD from diversified perspectives. Thus, we systematically collected 79 published signatures to compare with our TRLS model. After calculating the C-index of TRLS and other signatures across all cohorts, we noticed that many models performed significantly lower on other datasets than on their training dataset (e.g., Chen EG, Zhang A) (ref), which was generally considered overfitting (Fig. 4). Deficient sample sizes and misuse of machine-learning methods were important causes of overfitting. In contrast, TRLS was conferred superior generalizability from our integrative machine learning-based program so that it maintained the leading predictive performance in each cohort (Fig. 4). Overall, TRLS developed from our integrative machine-learning framework might be more suitable for clinical translation.

### Latent biological processes associated with TRLS

We further performed enrichment analysis through GO, KEGG, and HALLMARK to decode specific biological functions of LUAD patients with distinct TRLS. Patients with high TRLS enriched various biological processes linked to tumor progression (e.g., cell cycle, DNA replication, DNA damage, DNA repair), concordant with their dismal outcomes. Conversely, low TRLS patients were primarily associated with immune pathways and lipid metabolism processes (Additional file 5A). GSEA analysis also revealed that cell cycle, DNA replication, mismatch repair (MMR), homologous recombination (HR), and nucleotide excision repair (NER) pathways were markedly activated in the high TRLS group (Additional file 5B). A previous study reported that characteristics correlated with TMB including cell cycle, DNA replication, and DDR were potential signatures to predict response to PD-L1 blockade [42]. Overall, low-risk patients were characterized as tumor inhibition, while high-risk patients had a higher correlation with tumor promotion-associated biological processes.

### TRLS correlated with immune infiltration and tumor immunogenicity

**Fig. 5:**
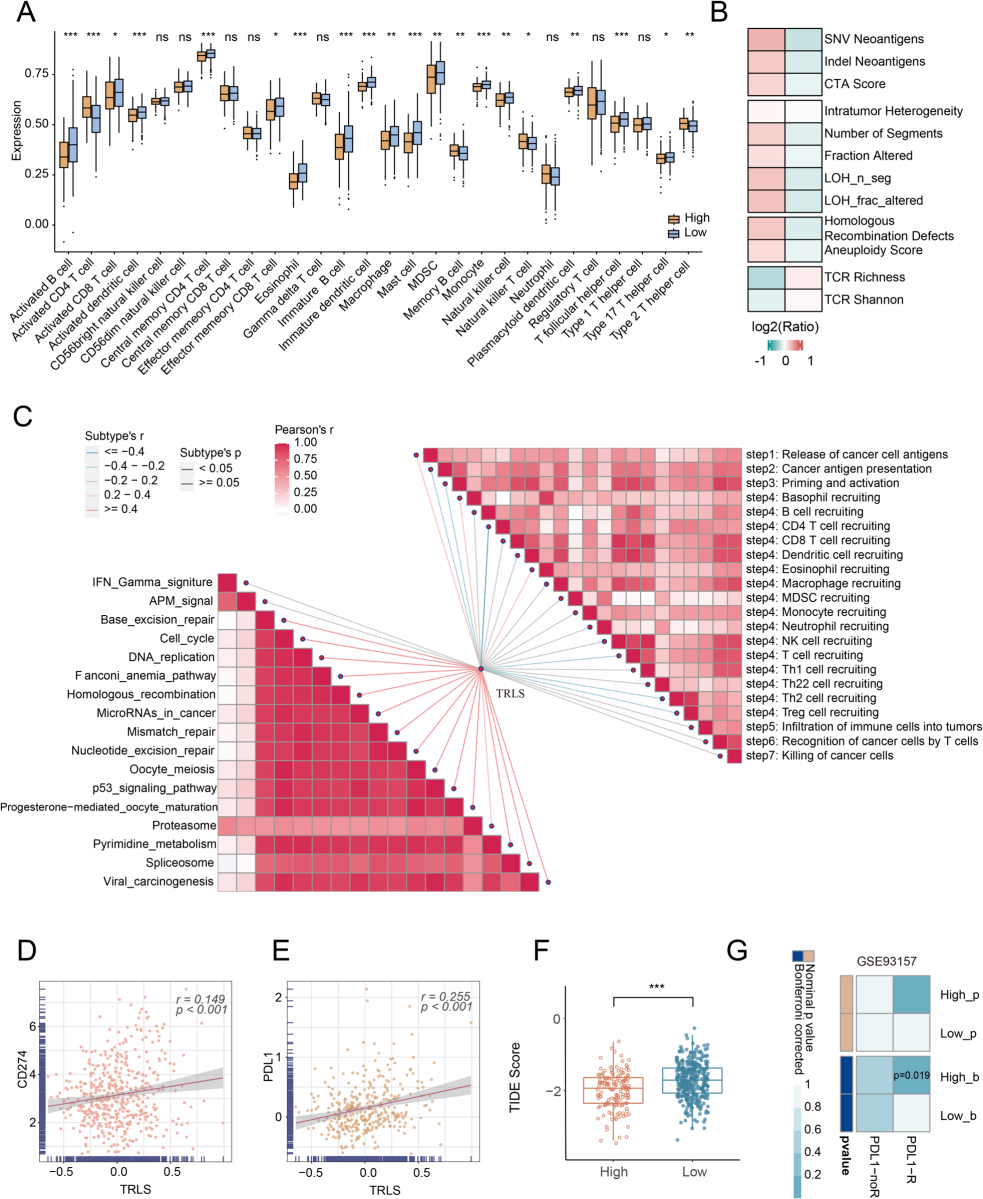
The distinct immune landscape and immunotherapy responses underlying TRLS. **A** The relative infiltration levels of 28 immune cell types. **B** Tumor immunogenicity evaluation between the high- and low-risk groups. **C** Correlation analysis of TRLS with cancer immune circulation (CIC) and immunotherapeutic signatures. **D**, **E** Correlations of TRLS with CD274 (**D**) and PD-L1 protein (**E**). **F** The distribution of TIDE scores between the two risk groups. **G** The SubMap analysis assessed the similarity of expression profile between the independent immunotherapy and the two risk groups. *P* values are shown as *P < 0.05, **P < 0.01, and ***P < 0.001

To further explore the potential TIME characteristics, we performed an immune microenvironment assessment for LUAD patients stratified by TRLS. Compared to patients with high TRLS, those with low TRLS possessed a higher stromal score, immune score, and ESTIMATE score (Additional file 6A). To further evaluate the immune infiltration, we quantified the relative infiltration levels of 28 immune cell types in the two groups. Higher infiltration levels of immune cells were exhibited in patients with high TRLS, especially activation of tumor-killing-related immune cells, such as CD8 + T cells, macrophages, and NKT cells (Fig. 5A), indicating patients with low TRLS tended to be the “immune-hot” tumors. Besides, the low TRLS group was dominant in the expressing HLA molecules representing cancer antigen presentation capacity (Additional file 6B). Moreover, patients with low TRLS also showed higher TCR richness and Shannon entropy of TCR diversity, whereas patients with high TRLS were remarkably featured by high levels of single-nucleotide variant (SNV) neoantigens, indel neoantigens, cancer-testis antigens (CTA) score, intratumor heterogeneity (ITH), fraction altered, number of segments, number of segments with loss of heterozygosity (LOH) events (LOH_n_sig), bases fraction with LOH events (LOH_frac_altered), homologous recombination defects (HRD), and aneuploidy score (Fig. 5B). Taken together, patients with low TRLS displayed higher infiltration of immune cells, while patients with high TRLS exhibited lower immune cells infiltration and significant genome instability.

### Implications of TRLS for immunotherapeutic response

To explore the association between TRLS and immunotherapeutic response, we evaluated the correlation of TRLS with immunotherapy signature and cancer immune circulation (CIC). In CIC, TRLS was positively associated with cancer cell antigen release and negatively correlated with CD4 + T cell recruitment and cancer antigen presentation, consistent with the assessment of immune status and genomic alterations. Interestingly, TRLS showed a strong positive correlation with a majority of immunotherapeutic signatures, such as cell cycle, DNA replication, MMR, HR, and NER (Fig. 5C). Further research demonstrated that the expression of the immune checkpoints, including CD70, TNFSF4, TNFSF9, CD274, and LAG3, was raised in the high TRLS group (Additional file 6C, D). Following elevated immune checkpoint expression, TRLS was positively correlated with CD274 and PD-L1 protein, hinting at improved effectiveness in immunotherapy (Fig. 5D, E). To generate deep insight into immunotherapy, we introduced TIDE and SubMap methods to estimate the clinical benefit for patients with diverse TRLS. As things turn out, lower expression of TIDE score was detected in the high-risk group, implying lower T cell dysfunction levels and elevated response to immunotherapy (*P* < 0.0001) (Fig. 5F). The results of SubMap analysis suggested high TRLS patients’ expression profiles were more similar to that of immunotherapy responders in nine independent immunotherapy cohorts (Fig. 5G, Additional file 6E). Overall, patients with high TRLS were fitter for immunotherapy.

### Potential genomic alterations of TRLS

**Fig. 6:**
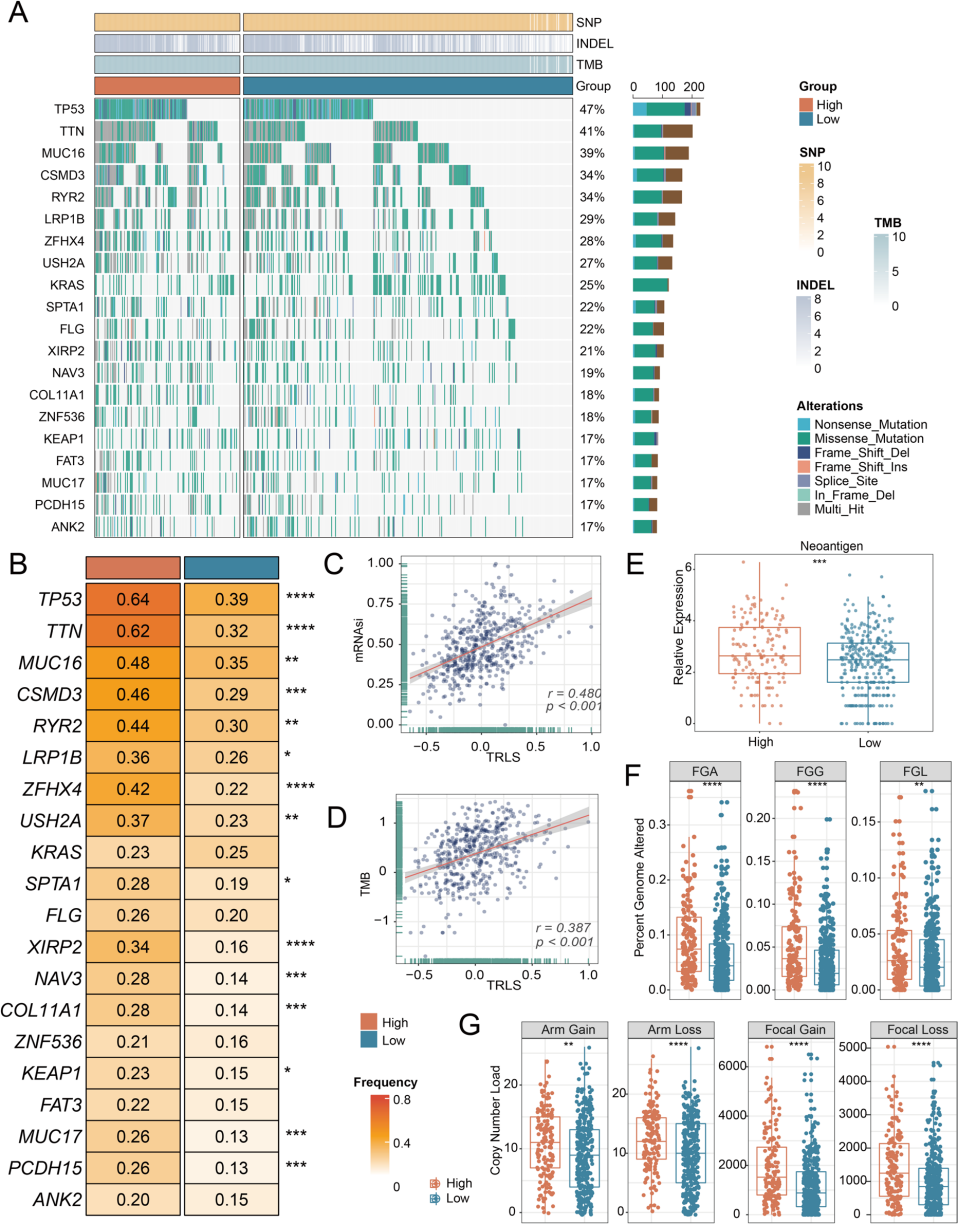
The genomics alteration underlying TRLS. **A** The waterfall plot depicted the differences in frequently mutated genes (FMGs) of lung adenocarcinoma between the two groups. **B** Top 20 FMGs at somatic mutations between the high- and low-risk groups. **C**, **D** Correlations analysis of TRLS with mRNAsi (**C**) and TMB (**D**). **E**–**G** Distribution of tumor neoantigen number (**E**); FGA, FGG, and FGL (**F**); and copy number load (**G**) in the high- and low-risk groups. *P* values are shown as *P < 0.05, **P < 0.01, and ***P < 0.001

As the above findings indicated, high TRLS displayed a higher correlation with genome instability. Thus, somatic mutation and CNV information were further explored to analyze TRLS-related genomic alterations. By exhibiting and further comparing the top 20 mutant genes’ mutation incidence between the 2 groups, it was revealed that the high TRLS group harbored an overall higher mutated frequency (Fig. 6A, B), especially significantly elevated TP53 and TTN mutations. As a general mutation in LUAD, TP53 mutation gives rise to genomic instability and results in high TMB, contributing to more malignant and worse outcomes in LUAD [48–50]. In line with somatic mutation frequency, correlation analysis further exhibited that TRLS was positively correlated with mRNA-si and TMB (Fig. 6C, D). The number of neoantigens was profoundly increased in the high TRLS group (Fig. 6E). Compared to patients with low TRLS, patients with high TRLS possessed an elevated mutation burden. To delve further into the genomic variations, the CNV was compared and estimated from multiple perspectives encompassing bases, fragments, and chromosome arms (Fig. 6F, G). As illustrated in Fig. 6F, FGA, FGG, and FGL in the high TRLS group were higher than those in the low TRLS group (Fig. 6F), which suggested a higher likelihood of cell proliferation and immune escape [51]. Meanwhile, conspicuous amplification and deletion were detected in the high TRLS group at both focal and arm levels (Fig. 6G). In summary, prominent genomic alterations were revealed for patients with high TRLS, representing higher genomic instability, while patients with low TRLS were regarded as a stable genome subtype.

## Discussion

In this study, we comprehensively identified TIME-related lncRNAs via integrating scRNA-seq and bulk RNA-seq data. Subsequently, an integrative machine-learning framework was introduced to generate an optimal model (termed TRLS) from 96 algorithm combinations. With the cross-platform validations and model comparisons, we believe that TRLS could serve as a promising platform for LUAD prognosis prediction. Additionally, to gain deep insights into TRLS, we further explore the potential biological functions and molecular alterations underlying the TRLS model, which laid the foundation for the decipherable investigation of TRLS.

Tumor, node, and metastasis (TNM) classification are essential indicators for evaluating the LUAD patients’ prognosis and making clinical decisions [52]. In recent years, molecular biomarkers such as TP53, EGFR, KRAS, and ALK mutation have played an increasingly important role in assessing prognosis and clinical strategies in LUAD [3, 16, 53]. Remarkably, our TRLS exhibited a more outstanding performance in predicting prognosis than these factors and other general clinical features including age, gender, and smoking. These findings indicated that TRLS was a promising signature in estimating prognosis for LUAD patients. Meanwhile, in 2 LUAD cell lines, we discovered that the knockdown of Lnc00857 pronouncedly inhibited cell proliferation, migration, and invasion, which further confirmed the reliability of our research. Furthermore, 79 published signatures based on the gene expression profiles from different biological processes were retrieved. Few of these signatures were rarely thoroughly validated or incorporated into clinical practice. Influenced by overfitting, most of these signatures performed robustly in the training cohort and insufficient validation cohorts, while the performance in other cohorts was significantly unsatisfactory. On the contrary, TRLS was conferred superior generalizability from our integrative machine learning-based program and revealed stable competence.

Beyond evaluating the robustness of TRLS, it was essential to decode TRLS from multiple perspectives to promote its clinical application. Firstly, our study delineated the underlying biological processes of patients with distinct TRLS. Functional enrichment analyses revealed low TRLS patients were dominantly connected with immune-related and lipid metabolic pathways, while high TRLS patients harbored conspicuous activation of genomic variation-related tumor promotion-associated pathways. In malignant tumors, production, intake, and storage were significantly upregulated to promote cancer cell proliferation and survival [54, 55]. Hence, significantly activated lipid metabolism-related pathways in low TRLS patients suggested tumor suppression. Notably, DDR-associated pathways were activated in high TRLS patients, which suggested high genome instability and copious tumor immunogenicity. Consistent with that, distinct genomic characteristics of LUAD patients with different TRLS were displayed. Specifically speaking, high TRLS patients possessed notably elevated somatic mutation frequency and CNV, indicating genome instability. Previous research revealed that TP53 mutations were associated with active DNA damage repair (DDR) and cell proliferation levels [56], which was congruent with tumor stemness evaluation in our study. Besides, an increased risk of immune escape and dismal prognosis appeared with additional TP53 mutation [57]. Correspondingly, with higher TP53 mutation, high TRLS patients displayed more active DDR and inferior clinical outcomes. Subsequently, for patients with divergent TRLS, we further probed the underlying signature of immune infiltration and tumor immunogenicity. Patients with low TRLS were elucidated by abundant immune infiltration and higher antigen presentation-related molecule expression, which presented an “immune-hot” phenotype. In contrast with them, our research exhibited that patients with high TRLS, with insufficient immune infiltration and tight connection with the genomic alteration-associated immune escape, signaled salient genome instability and immunogenicity.

Development and clinical application of tumor immunotherapy have revolutionized treatment patterns in LUAD. As is well known, immunogenicity was a crucial factor in the immunotherapy effects of LUAD patients. Therefore, based on the above findings, we turned our attention to the implications of TRLS for immunotherapeutic response. Recently, TMB, neoantigens, and PD-L1 expression were recognized as predictive indicators of responsiveness to PD-1/PD-L1 inhibitors [58]. In our study, high TRLS patients exhibited higher TMB and neoantigens, which was consistent with apparent genome instability as described above. For LUAD patients, increased TMB and neoantigens signal strengthened cytotoxic T lymphocyte proliferation and activation [59]. Besides, a close relationship between high TRLS patients and DDR was demonstrated in our study. DDR has been a promising pharmacological immune checkpoint inhibitor (ICI) target for its unique influence on multiple respects of tumor immunogenicity, such as autonomous tumor cell responses and tumor cell microenvironment interactions [60]. Furthermore, CD274 and PD-L1 protein raised with the increase in TRLS, which was associated with immune escape and played a negative regulatory role. As two common assessment strategies for immunotherapy, TIDE and SubMap demonstrated patients with high TRLS benefit more from immunotherapy. Taken together, TRLS was a latent signature for immunotherapy effect evaluation. Patients with high TRLS were recommended to take more consideration for immunotherapy. Overall, low TRLS patients were characterized by stable genomic features, superior immune activity, and good clinical outcomes. Notably, low TRLS harbored copious immune infiltration but displayed insensitivity to immunotherapy. Absent immunogenicity held a major contribution to that, performing as lower TMB and neoantigens. In contrast, high TRLS patients displayed significant genome instability, elevated tumor immunogenicity, insufficient immune infiltration, and dismal prognosis. Our study demonstrated the benefits of immunotherapy of high TRLS patients, which was an effective approach to improving their prognoses.

Although TRLS was an attractive platform for evaluating the prognosis of LUAD. Nevertheless, several limitations should be recognized. First, all samples were retrospective data in our current research, prospective study should be performed for further validation. Second, incomplete data in some cohorts may hinder the investigation of the relationship between TRLS and some features. Next, appropriate immunotherapy patients from a multicenter and large sample cohort were required to enforce the clinical efficacy evaluation. Last but not least, additional experiments in vivo and in vitro were still required to explore the underlying biological functions of TRLS.

In general, this study incorporated single-cell and bulk data to develop and validate a robust signature (termed TRLS) in 6 independent cohorts according to the integrative machine-learning framework. The superior performance of TRLS was further confirmed by comparing clinical features, molecular characters, and 79 published signatures. Specifically, patients with low TRLS were distinguished by favorable prognosis and abundant immune cell infiltration, whereas high TRLS patients were endowed with a dismal prognosis, high levels of genome instability and immunogenicity, and potential sensitivity to immunotherapy. Overall, this study could facilitate the prognostic management and precision treatment of LUAD patients.

## Conclusions

Our study developed a promising tool based on TIME-related lncRNAs, which identified LUAD patients with different risk scores and might contribute to the tailored treatment and prognosis management of LUAD patients.

### Supplementary Information

**Additional file 1:** **Fig. S1.** The distribution of gene number of cells after quality control.

**Additional file 2:** **Fig. S2.** (A-B) PAC score and CDF curve of consensus matrix for each k (from 2 to 9). A low value of PAC implies a flat middle segment, allowing conjecture of the optimal k (k = 2) by the lowest PAC. (C) Immune infiltration assessment for two clusters via multiple algorithms. (D) Correlation analysis between module eigengenes and different clusters based on WGCNA. (E-G) The high correlation between module membership and gene significance in the module 4 (E), module 13 (F), and module 5 (G).

**Additional file 3:** **Fig. S3.** The relative immune cell distribution through multiple algorithms for two immune subtypes.

**Additional file 4:** **Fig. S4.** (A) Thirty-three prognosis-related lncRNAs associated with prognosis were identified through univariate Cox analysis. (B) Kaplan-Meier survival analysis of OS according to TRLS in six independent cohorts, including TCGA-LUAD, GSE72094, GSE50081, GSE31210, GSE30219, and GSE3141. (C) LncRNA knockdown inefficiency figures of two cell lines.

**Additional file 5: Fig. S5.** Latent biological processes of two risk groups.

**Additional file 6: Fig. S6.** (A) Immune infiltration evaluation based on ESTIMATE algorithm in the high- and low-risk groups. (B) Boxplot of antigen presentation molecules expression levels between two groups. (C-D) Boxplot of expression profiles for co-stimulatory (C) and co-inhibitory (D) immune checkpoint genes between two groups. (E) SubMap plots evaluated the similarity of gene expression profiles between two groups and eight immunotherapy cohorts.

**Additional file 7: Table S1.** Details of baseline information in 15 public datasets.

**Additional file 8: Table S2.** TIME-related genes identified from scRNA-seq analysis.

**Additional file 9: Table S3.** TIME-related genes with prognostic value.

**Additional file 10: Table S4.** LncRNAs from associated modules between distinct immune infiltration clusters.

**Additional file 11: Table S5.** Prognostic value of 33 lncRNAs in six cohorts.

**Additional file 12: Table S6.** Formula for the calculation of TRLS.

**Additional file 13: Table S7.** Individual TRLS for patients in six cohorts.

**Additional file 14: Table S8.** Multivariate Cox regression of TRLS regarding to OS.

## Data Availability

Publicly available datasets analyzed during the current study are available in the TCGA database and GEO database under accession codes. TCGA (https://www.cancer.gov/tcga), GSE171145 (https://www.ncbi.nlm.nih.gov/geo/query/acc.cgi?acc=GSE171145) [16], GSE72094 (https://www.ncbi.nlm.nih.gov/geo/query/acc.cgi?acc=GSE72094) [17], GSE50081 (https://www.ncbi.nlm.nih.gov/geo/query/acc.cgi?acc=GSE50081) [18], GSE31210 (https://www.ncbi.nlm.nih.gov/geo/query/acc.cgi?acc=GSE31210) [19], GSE30219 (https://www.ncbi.nlm.nih.gov/geo/query/acc.cgi?acc=GSE30219) [20], GSE3141 (https://www.ncbi.nlm.nih.gov/geo/query/acc.cgi?acc=GSE3141) [21], GSE35640 (https://www.ncbi.nlm.nih.gov/geo/query/acc.cgi?acc=GSE35640) [22], GSE78220 (https://www.ncbi.nlm.nih.gov/geo/query/acc.cgi?acc=GSE78220) [23], GSE91061 (https://www.ncbi.nlm.nih.gov/geo/query/acc.cgi?acc=GSE91061) [24], GSE93157 (https://www.ncbi.nlm.nih.gov/geo/query/acc.cgi?acc=GSE93157) [25], GSE100797 (https://www.ncbi.nlm.nih.gov/geo/query/acc.cgi?acc=GSE100797) [26], GSE115821 (https://www.ncbi.nlm.nih.gov/geo/query/acc.cgi?acc=GSE115821) [27], GSE126044 (https://www.ncbi.nlm.nih.gov/geo/query/acc.cgi?acc=GSE126044) [28], GSE136961 (https://www.ncbi.nlm.nih.gov/geo/query/acc.cgi?acc=GSE136961) [29], and GSE145996 (https://www.ncbi.nlm.nih.gov/geo/query/acc.cgi?acc=GSE145996) [30]. The core scripts to process and analyze the data are available at https://github.com/zzuliul/TRLS.

## Abbreviations

TIME: Tumor immune microenvironment
LUAD: Lung adenocarcinoma
TRLS: TIME-related lncRNA signature
DDR: DNA damage repair
scRNA-seq: Single-cell RNA sequencing
PCA: Principal component analysis
UMAP: Uniform Manifold Approximation and Projection
RMA: Robust multi-array average
WGCNA: Weighted correlation network analysis
TOM: Topological overlap matrix
GO: Gene Ontology
KEGG: Kyoto Encyclopedia of Genes and Genomes
GSEA: Gene set enrichment analysis
SNV: Single-nucleotide variant
CTA: Cancer-testis antigens
ITH: Intratumor heterogeneity
LOH: Loss of heterozygosity
HRD: Homologous recombination defects
CIC: Cancer immune circulation
CNV: Copy number variation
TMB: Tumor mutation burden
