# Supplementary figures and images for: Single-cell RNA sequencing integrated with bulk RNA sequencing analysis identifies a tumor immune microenvironment-related lncRNA signature in lung adenocarcinoma
Source: BMC Biol. 2024 Mar 22;22:69. doi: 10.1186/s12915-024-01866-5 (PMC10960411; doi:10.1186/s12915-024-01866-5)

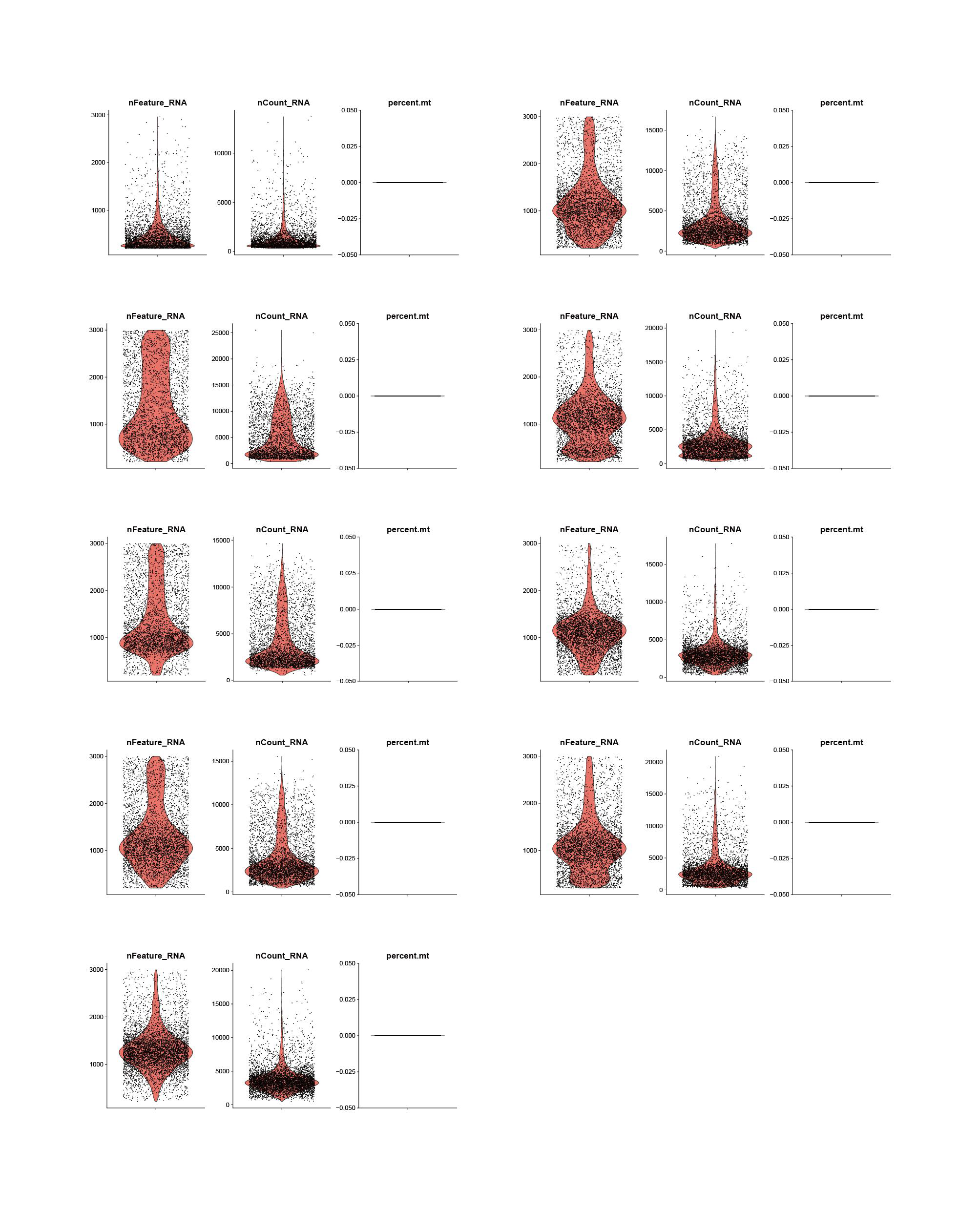

Supplement: Supplementary file 1 — Additional file 1: Fig. S1. The distribution of gene number of cells after quality control. [file 12915_2024_1866_MOESM1_ESM.png]

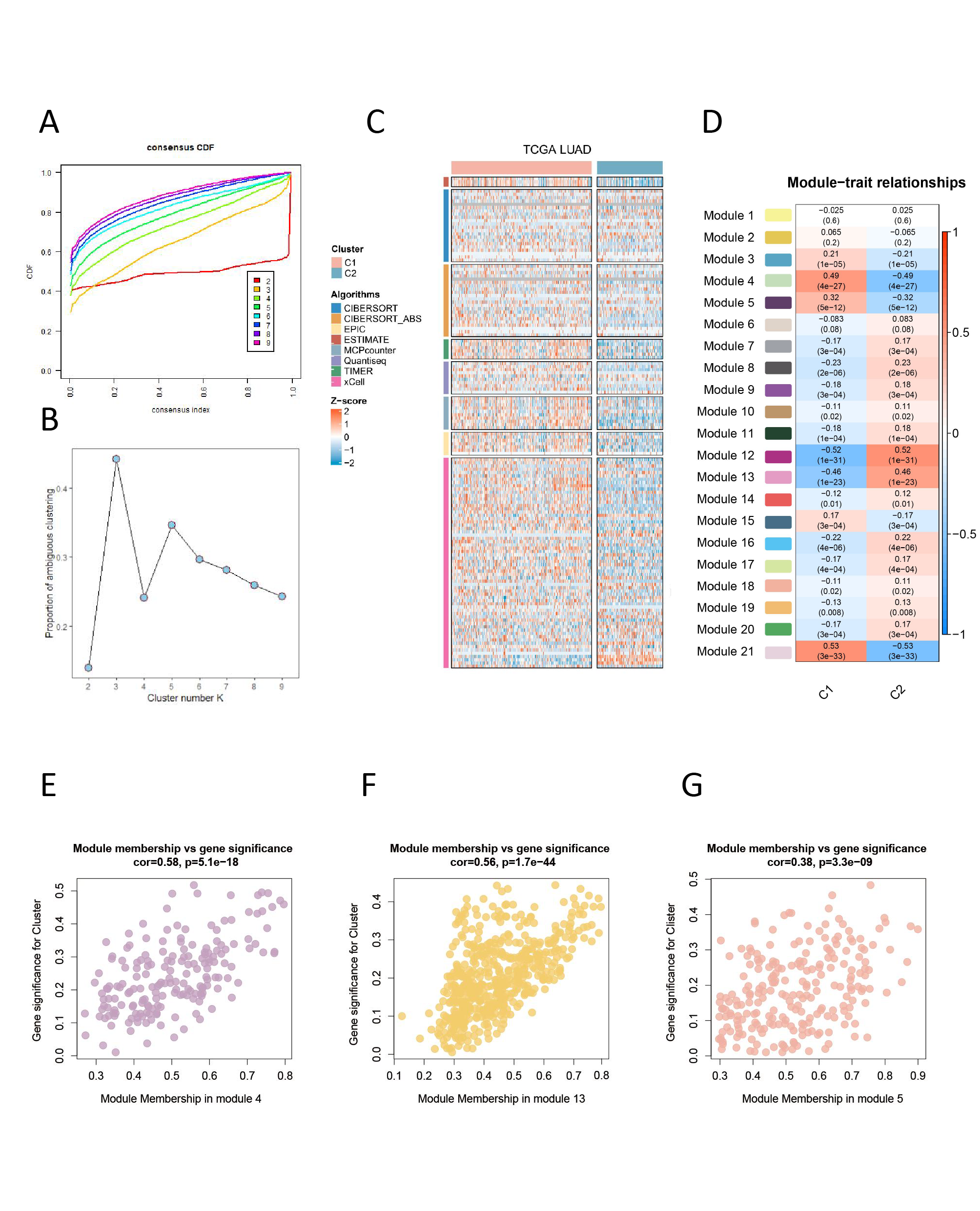

Supplement: Supplementary file 2 — Additional file 2: Fig. S2. (A-B) PAC score and CDF curve of consensus matrix for each k (from 2 to 9). A low value of PAC implies a flat middle segment, allowing conjecture of the optimal k (k = 2) by the lowest PAC. (C) Immune infiltration assessment for two clusters via multiple algorithms. (D) Correlation analysis between module eigengenes and different clusters based on WGCNA. (E-G) The high correlation between module membership and gene significance in the module 4 (E), module 13 (F), and module 5 (G). [file 12915_2024_1866_MOESM2_ESM.png]

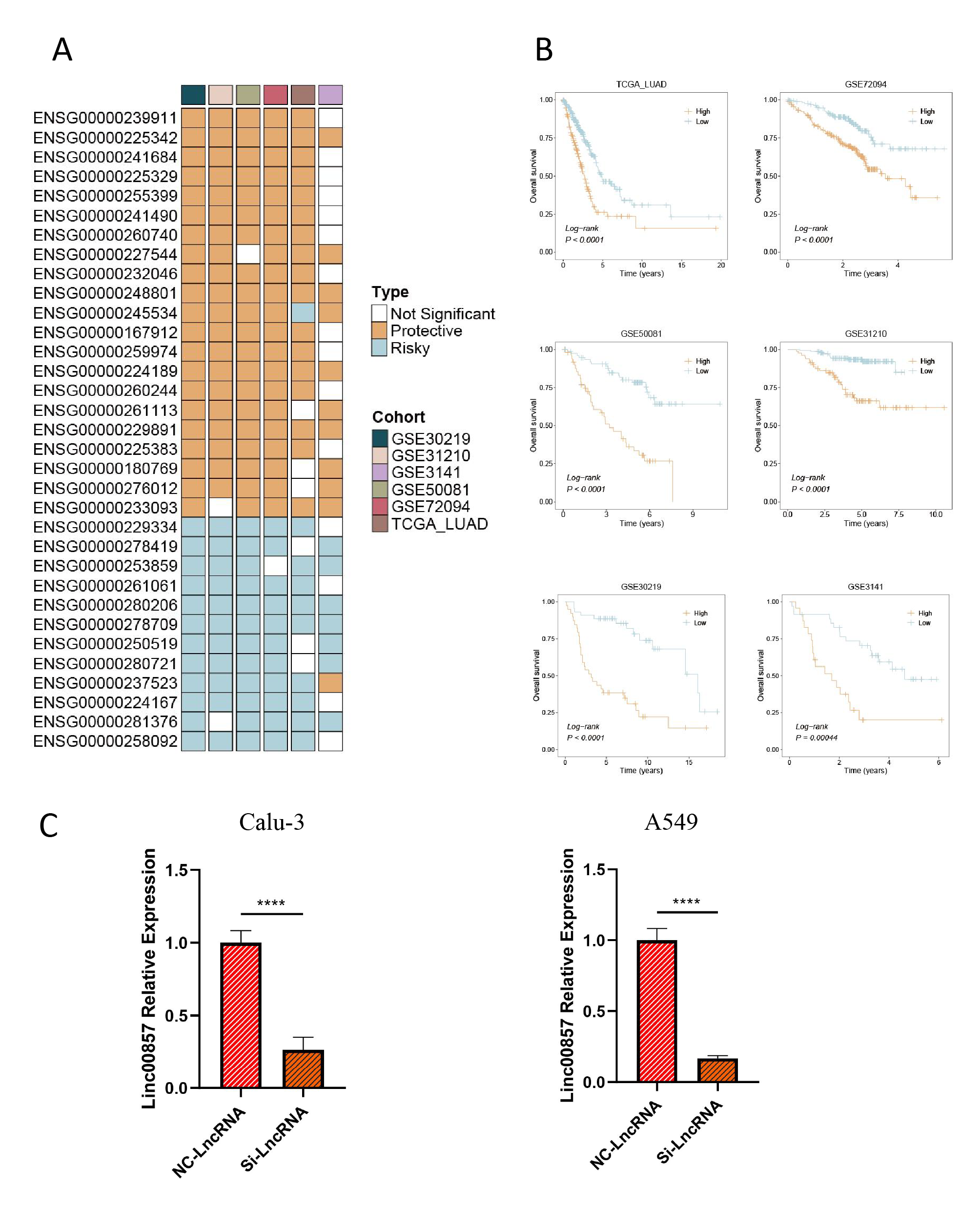

Supplement: Supplementary file 3 — Additional file 3: Fig. S3. The relative immune cell distribution through multiple algorithms for two immune subtypes. [file 12915_2024_1866_MOESM3_ESM.png]

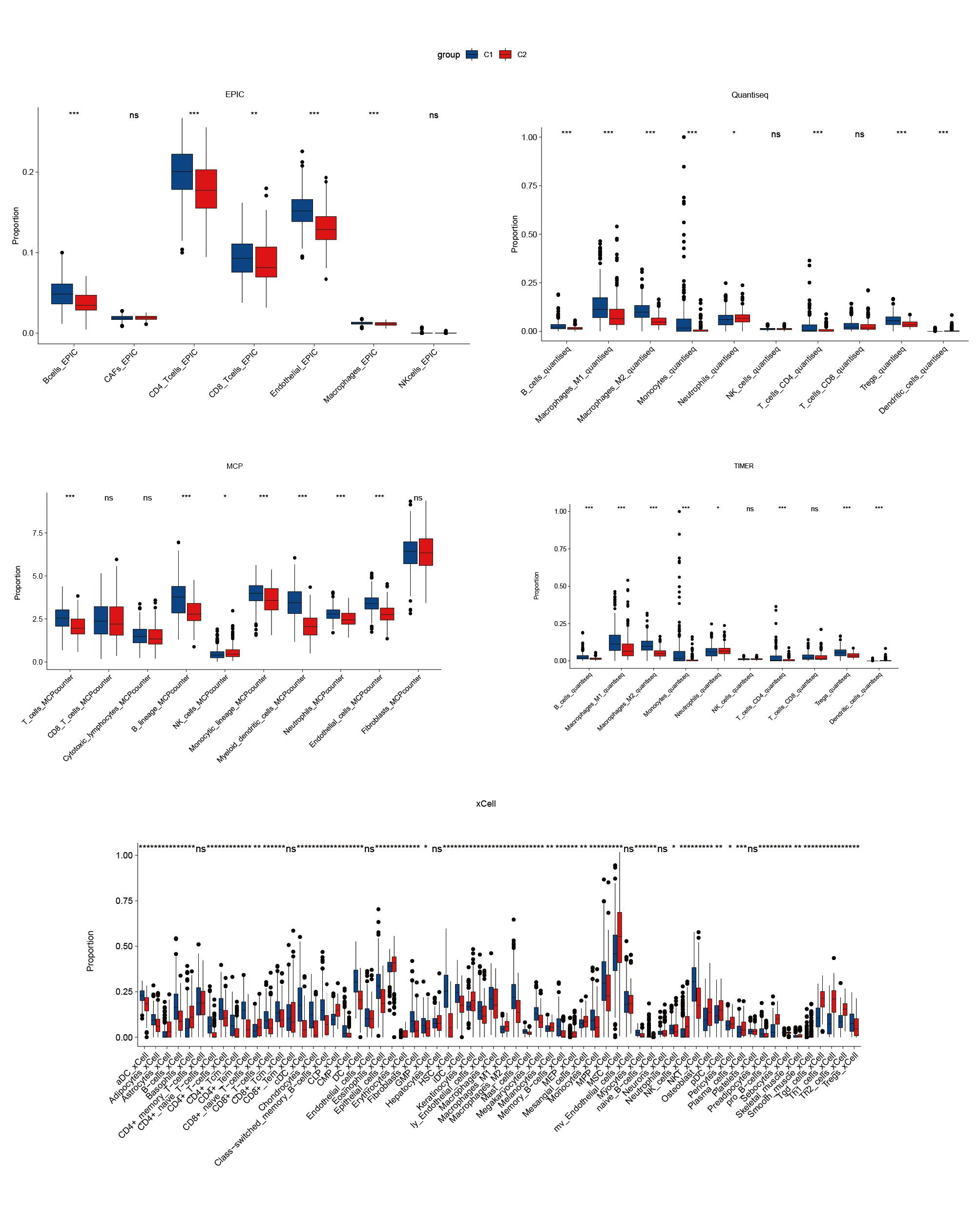

Supplement: Supplementary file 4 — Additional file 4: Fig. S4. (A) Thirty-three prognosis-related lncRNAs associated with prognosis were identified through univariate Cox analysis. (B) Kaplan-Meier survival analysis of OS according to TRLS in six independent cohorts, including TCGA-LUAD, GSE72094, GSE50081, GSE31210, GSE30219, and GSE3141. (C) LncRNA knockdown inefficiency figures of two cell lines. [file 12915_2024_1866_MOESM4_ESM.png]

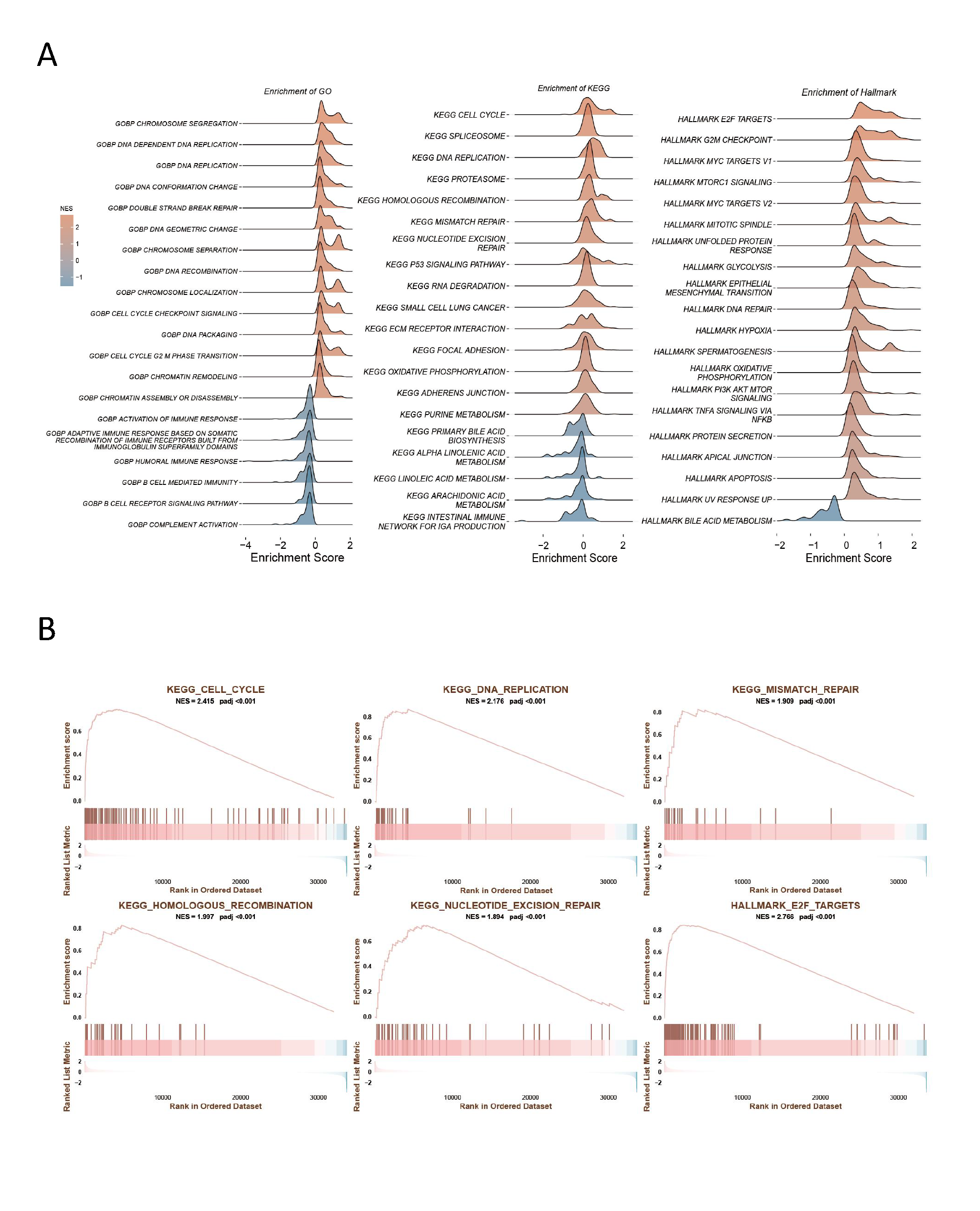

Supplement: Supplementary file 5 — Additional file 5: Fig. S5. Latent biological processes of two risk groups. [file 12915_2024_1866_MOESM5_ESM.png]

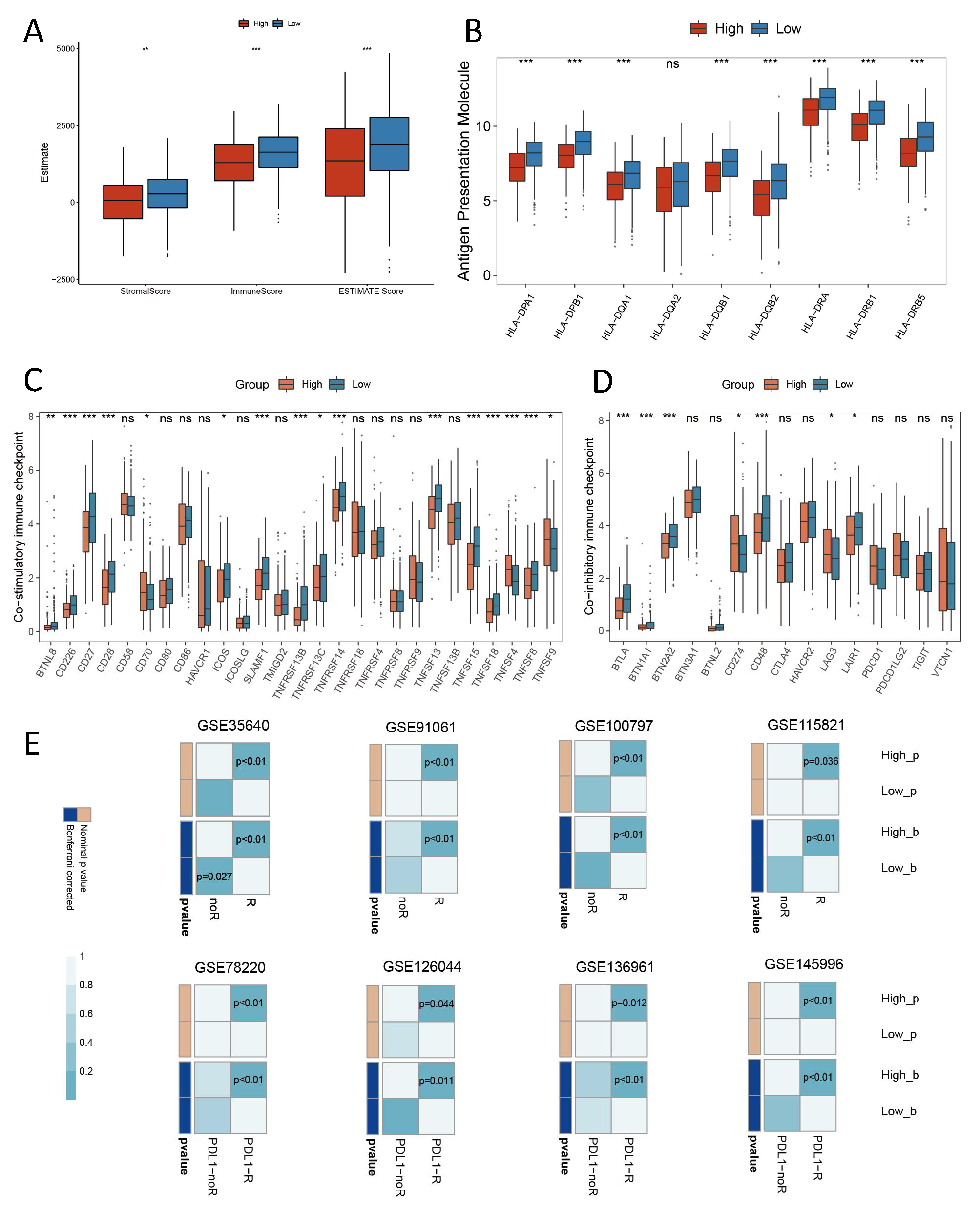

Supplement: Supplementary file 6 — Additional file 6: Fig. S6. (A) Immune infiltration evaluation based on ESTIMATE algorithm in the high- and low-risk groups. (B) Boxplot of antigen presentation molecules expression levels between two groups. (C-D) Boxplot of expression profiles for co-stimulatory (C) and co-inhibitory (D) immune checkpoint genes between two groups. (E) SubMap plots evaluated the similarity of gene expression profiles between two groups and eight immunotherapy cohorts. [file 12915_2024_1866_MOESM6_ESM.png]
