# Supplementary material for: Single-cell RNA sequencing integrated with bulk RNA sequencing analysis identifies a tumor immune microenvironment-related lncRNA signature in lung adenocarcinoma
Source: BMC Biol. 2024 Mar 22;22:69. doi: 10.1186/s12915-024-01866-5 (PMC10960411; doi:10.1186/s12915-024-01866-5)
Supplement: Supplementary file 7 — Additional file 7: Table S1. Details of baseline information in 15 public datasets. [file 12915_2024_1866_MOESM7_ESM.pdf]

Table S1. Details of baseline information in 15 public datasets

Accession	TCGA-LUAD	GSE30219	GSE31210	GSE3141	GSE50081	GSE72094
Number of Patients (%)	497 (100%)	85 (100%)	246 (100%)	58 (100%)	127 (100%)	442 (100%)
Event						
Alive	317 (63.8%)	40 (47.1%)	191 (77.6%)	26 (44.8%)	76 (59.8%)	298 (67.4%)
Dead	180 (36.2%)	45 (52.9%)	35 (14.2%)	32 (55.2%)	51 (40.2%)	122 (27.6%)
Not available	--	--	20 (8.1%)	--	--	22 (5.0%)
Age						
>65	251 (50.5%)	25 (29.4%)	52 (21.1%)	--	87 (68.5%)	294 (66.5%)
≤65	236 (47.5%)	60 (70.6%)	189 (76.8%)	--	40 (31.5%)	127 (28.7%)
Not available	10 (2.0%)	--	5 (2.0%)		--	21 (4.8%)
Gender						
Female	269 (54.1%)	19 (22.4%)	130 (52.8%)	--	62 (48.8%)	240 (54.3%)
Male	228 (45.9%)	66 (77.6%)	116 (47.2%)	--	65 (51.2%)	202 (45.7%)
Stage						
I	267 (53.7%)	--	168 (68.3%)	--	92 (72.4%)	265 (60.0%)
II	118 (23.7%)	--	58 (23.6%)	--	35 (27.6%)	69 (15.6%)
III	80 (16.1%)	--	--	--	--	63 (14.3%)
IV	25 (5.0%)	--	--	--	--	17 (3.8%)
Not available	7 (1.4%)	--	20 (8.1%)	--	--	28 (6.3%)
T1	166 (33.4%)	71 (83.5%)	--	--	43 (33.9%)	--
T2	267 (53.7%)	12 (14.1%)	--	--	82 (64.6%)	--
T3	43 (8.7%)	2 (2.4%)	--	--	2 (1.6%)	--
T4	18 (3.6%)	--	--	--	--	--
Not available	3 (0.6%)	--	--	--	--	--
N						
N0	321 (64.6%)	82 (96.5%)	--	--	94 (74.0%)	--
N1	94 (18.9%)	3 (3.5%)	--	--	33 (26.0%)	--
N2	69 (13.9%)	--	--	--	--	--
N3	2 (0.4%)	--	--	--	--	--
Not available	11 (2.2%)	--	--	--	--	--
M						
M0	331 (66.6%)	85 (100.0%)	--	--	127 (100.0%)	--
M1	24 (4.8%)	--	--	--	--	--
Not available	142 (28.6%)	--	--	--	--	--
Smoke						
No	--	--	123 (50.0%)	--	23 (18.1%)	33 (7.5%)
Yes	--	--	123 (50.0%)	--	92 (72.4%)	335 (75.8%)
Not available	--	--	--	--	12 (9.4%)	74 (16.7%)
EGFR						
Mut	--	--	127 (51.6%)	--	--	47 (10.6%)
WT	--	--	99 (40.2%)	--	--	395 (89.4%)
Not available	--	--	20 (8.1%)	--	--	
KRAS						
Mut	--	--	20 (8.1%)	--	--	154 (34.8%)
WT	--	--	206 (83.7%)	--	--	288 (65.2%)
Not available	--	--	20 (8.1%)	--	--	--
ALK						
Mut	--	--	11 (4.5%)	--	--	--
WT	--	--	215 (87.4%)	--	--	--
Not available	--	--	20 (8.1%)	--	--	--
TP53						
Mut	--	--	--	--	--	111 (25.1%)

WT	--	--	--	--	--	331 (74.9%)
STK11						
Mut	--	--	--	--	--	68 (15.4%)
WT	--	--	--	--	--	374 (84.6%)
Race						
Asian	--	--	--	--	--	3 (0.7%)
Black	--	--	--	--	--	13 (2.9%)
White	--	--	--	--	--	399 (90.3%)
Not available	--	--	--	--	--	27 (6.1%)

**Accession**	**GSE35640**	**GSE78220**	**GSE91061**	**GSE93157**	**GSE100797**	**GSE115821**	**GSE126044**	**GSE136961**	**GSE145996**
Number of Patients (%)	65 (100%)	28 (100%)	109 (100%)	65 (100%)	21 (100%)	37 (100%)	16 (100%)	21 (100%)	14 (100%)
Response									
NR	34 (52.4%)	13 (46.4%)	82 (75.2%)	45 (69.2%)	13 (61.9%)	34 (91.9%)	11 (68.8%)	12 (57.1%)	6 (42.9%)
R	22 (33.8%)	15 (53.6%)	23 (21.1%)	20 (30.8%)	8 (38.1%)	3 (8.1%)	5 (31.2%)	9 (42.9%)	8 (57.1%)
Not available	9 (13.8%)	--	4 (3.7%)	--	--	--	--	--	--
