# Supplementary material for: Single-cell RNA sequencing integrated with bulk RNA sequencing analysis identifies a tumor immune microenvironment-related lncRNA signature in lung adenocarcinoma
Source: BMC Biol. 2024 Mar 22;22:69. doi: 10.1186/s12915-024-01866-5 (PMC10960411; doi:10.1186/s12915-024-01866-5)
Supplement: Supplementary file 8 — Additional file 8: Table S2. TIME-related genes identified from scRNA-seq analysis. [file 12915_2024_1866_MOESM8_ESM.pdf]

Table S2. TIME-related genes identified from scRNA-seq analysis.

gene	p_val	avg_log2FC	pct.1	pct.2	p_val_adj	cell type
ENSG00000102245	0	19.2879761	0.811	0.708	0	CD4+ T cells
ENSG00000140044	0	-2.164383111	0.707	0.774	0	CD4+ T cells
ENSG00000163600	0	-4.503687774	0.88	0.773	0	CD4+ T cells
ENSG00000012779	0	-5.894337544	0.743	0.818	0	CD4+ T cells
ENSG00000125810	0	-6.290119249	0.698	0.797	0	CD4+ T cells
ENSG00000167703	0	-6.320873722	0.738	0.78	0	CD4+ T cells
ENSG00000101336	0	-8.101627907	0.794	0.848	0	CD4+ T cells
ENSG00000100311	0	-9.905480126	0.465	0.667	0	CD4+ T cells
ENSG00000066336	0	-11.12365068	0.801	0.852	0	CD4+ T cells
ENSG00000000938	0	-11.62833932	0.78	0.836	0	CD4+ T cells
ENSG00000140678	0	-12.84246258	0.745	0.787	0	CD4+ T cells
ENSG00000140749	0	-14.53868322	0.715	0.8	0	CD4+ T cells
ENSG00000035862	0	-14.95263041	0.612	0.757	0	CD4+ T cells
ENSG00000163563	0	-15.83510292	0.745	0.799	0	CD4+ T cells
ENSG00000168461	0	-16.20003347	0.768	0.815	0	CD4+ T cells
ENSG00000254087	0	-16.61753701	0.711	0.812	0	CD4+ T cells
ENSG00000161921	0	-18.38652182	0.693	0.796	0	CD4+ T cells
ENSG00000132334	0	-18.9771332	0.547	0.711	0	CD4+ T cells
ENSG00000168329	0	-21.04105976	0.599	0.746	0	CD4+ T cells
ENSG00000221869	0	-21.90064518	0.707	0.81	0	CD4+ T cells
ENSG00000120594	0	-21.9437182	0.604	0.726	0	CD4+ T cells
ENSG00000204472	0	-22.36928077	0.806	0.875	0	CD4+ T cells
ENSG00000143226	0	-25.41229776	0.76	0.823	0	CD4+ T cells
ENSG00000131401	0	-28.91892597	0.765	0.819	0	CD4+ T cells
ENSG00000182578	0	-29.79355566	0.721	0.812	0	CD4+ T cells
ENSG00000103811	0	-30.62652453	0.873	0.922	0	CD4+ T cells
ENSG00000121552	0	-31.86207607	0.741	0.794	0	CD4+ T cells
ENSG00000229391	0	-32.51557478	0.836	0.886	0	CD4+ T cells
ENSG00000112715	0	-44.5138931	0.823	0.856	0	CD4+ T cells
ENSG00000148180	0	-46.45131776	0.796	0.859	0	CD4+ T cells
ENSG00000189067	0	-51.96493595	0.833	0.891	0	CD4+ T cells
ENSG00000237541	0	-53.63076276	0.77	0.829	0	CD4+ T cells
ENSG00000176788	0	-55.914785	0.659	0.761	0	CD4+ T cells
ENSG00000163347	0	-56.65460067	0.837	0.857	0	CD4+ T cells
ENSG00000179639	0	-56.99944585	0.78	0.83	0	CD4+ T cells
ENSG00000130066	0	-60.98084118	0.824	0.866	0	CD4+ T cells
ENSG00000260314	0	-69.23647002	0.766	0.845	0	CD4+ T cells
ENSG00000125730	0	-76.84473024	0.78	0.846	0	CD4+ T cells
ENSG00000170458	0	-78.46348243	0.744	0.808	0	CD4+ T cells
ENSG00000108179	0	-80.18270712	0.805	0.856	0	CD4+ T cells
ENSG00000158869	0	-83.10074444	0.811	0.895	0	CD4+ T cells
ENSG00000073756	0	-86.85485859	0.687	0.768	0	CD4+ T cells
ENSG00000112149	0	-89.7843054	0.871	0.89	0	CD4+ T cells

ENSG00000011600	0	-93.82292143	0.852	0.928	0	CD4+ T cells
ENSG00000140968	0	-97.72862176	0.8	0.826	0	CD4+ T cells
ENSG00000178726	0	-114.0983664	0.798	0.861	0	CD4+ T cells
ENSG00000197405	0	-126.9427632	0.801	0.87	0	CD4+ T cells
ENSG00000123689	0	-135.810876	0.854	0.89	0	CD4+ T cells
ENSG00000136689	0	-136.1418351	0.836	0.886	0	CD4+ T cells
ENSG00000173391	0	-163.4475126	0.876	0.923	0	CD4+ T cells
ENSG00000116741	0	-164.3276063	0.837	0.873	0	CD4+ T cells
ENSG00000115956	0	-169.8344726	0.646	0.751	0	CD4+ T cells
ENSG00000011422	0	-170.8530483	0.912	0.94	0	CD4+ T cells
ENSG00000223865	0	-211.0482601	0.85	0.894	0	CD4+ T cells
ENSG00000231389	0	-219.0000135	0.942	0.959	0	CD4+ T cells
ENSG00000196126	0	-235.3775397	0.949	0.958	0	CD4+ T cells
ENSG00000169429	0	-253.6278377	0.84	0.887	0	CD4+ T cells
ENSG00000276070	0	-254.0402139	0.92	0.952	0	CD4+ T cells
ENSG00000101439	0	-306.6603211	0.934	0.956	0	CD4+ T cells
ENSG00000276085	0	-312.0445286	0.898	0.912	0	CD4+ T cells
ENSG00000197746	0	-354.4162573	0.946	0.97	0	CD4+ T cells
ENSG00000075624	0	-384.5537001	0.978	0.984	0	CD4+ T cells
ENSG00000125538	0	-462.1213627	0.833	0.897	0	CD4+ T cells
ENSG00000204287	0	-574.0582274	0.995	0.997	0	CD4+ T cells
ENSG00000087086	0	-716.5021653	0.977	0.985	0	CD4+ T cells
ENSG00000019582	0	#NAME?	0.996	0.993	0	CD4+ T cells
ENSG00000135821	2.38E-304	-54.58809962	0.814	0.872	4.76E-301	CD4+ T cells
ENSG00000197249	1.04E-303	-50.98987007	0.821	0.886	2.08E-300	CD4+ T cells
ENSG00000204482	2.21E-303	-13.18012265	0.757	0.825	4.41E-300	CD4+ T cells
ENSG00000187116	1.29E-302	-3.556125296	0.412	0.617	2.59E-299	CD4+ T cells
ENSG00000135077	5.89E-302	-6.901945792	0.762	0.782	1.18E-298	CD4+ T cells
ENSG00000168878	2.36E-301	-99.3057134	0.979	0.987	4.72E-298	CD4+ T cells
ENSG00000196735	3.26E-298	-135.2176384	0.744	0.788	6.51E-295	CD4+ T cells
ENSG00000137462	1.70E-296	-5.914469557	0.756	0.814	3.39E-293	CD4+ T cells
ENSG00000143546	3.95E-294	-459.1975853	0.761	0.836	7.89E-291	CD4+ T cells
ENSG00000030582	3.51E-293	-184.0458004	0.845	0.89	7.03E-290	CD4+ T cells
ENSG00000162734	8.68E-293	-40.06658657	0.711	0.791	1.74E-289	CD4+ T cells
ENSG00000104763	2.24E-291	-21.34578086	0.633	0.748	4.48E-288	CD4+ T cells
ENSG00000143162	7.56E-288	-15.74244247	0.625	0.733	1.51E-284	CD4+ T cells
ENSG00000119535	7.55E-287	-32.38429491	0.746	0.796	1.51E-283	CD4+ T cells
ENSG00000101160	1.19E-283	-45.75079116	0.731	0.801	2.37E-280	CD4+ T cells
ENSG00000136826	6.91E-282	-61.99149327	0.743	0.809	1.38E-278	CD4+ T cells
ENSG00000126709	2.80E-281	-248.7354434	0.965	0.973	5.59E-278	CD4+ T cells
ENSG00000158481	2.77E-279	-59.53905375	0.812	0.845	5.55E-276	CD4+ T cells
ENSG00000090382	4.32E-278	-97.97827997	0.744	0.818	8.65E-275	CD4+ T cells
ENSG00000135404	4.58E-276	-50.45222981	0.76	0.831	9.16E-273	CD4+ T cells
ENSG00000176597	7.60E-276	-4.669963675	0.702	0.754	1.52E-272	CD4+ T cells
ENSG00000104870	1.21E-273	-22.3461716	0.693	0.777	2.42E-270	CD4+ T cells

ENSG00000198223	7.65E-273	-3.563613983	0.737	0.793	1.53E-269	CD4+ T cells
ENSG00000120262	3.31E-272	-2.030915884	0.653	0.734	6.62E-269	CD4+ T cells
ENSG00000170909	1.78E-269	-5.729869484	0.518	0.662	3.56E-266	CD4+ T cells
ENSG00000002933	3.55E-269	-26.88141406	0.534	0.678	7.11E-266	CD4+ T cells
ENSG00000116962	2.88E-268	-10.03539385	0.245	0.472	5.76E-265	CD4+ T cells
ENSG00000169896	3.66E-268	-8.361452237	0.774	0.782	7.33E-265	CD4+ T cells
ENSG00000165025	2.03E-267	-3.748150236	0.654	0.708	4.06E-264	CD4+ T cells
ENSG00000198053	6.54E-267	-7.293415992	0.694	0.75	1.31E-263	CD4+ T cells
ENSG00000180871	6.18E-266	-4.590327298	0.256	0.478	1.24E-262	CD4+ T cells
ENSG00000204388	1.71E-265	-174.0035162	0.981	0.985	3.42E-262	CD4+ T cells
ENSG00000171051	3.58E-264	-16.29947571	0.719	0.766	7.16E-261	CD4+ T cells
ENSG00000261040	5.20E-264	-44.27735744	0.839	0.854	1.04E-260	CD4+ T cells
ENSG00000277443	8.12E-264	-18.40325497	0.644	0.711	1.62E-260	CD4+ T cells
ENSG00000111275	3.01E-263	-48.31139227	0.8	0.84	6.01E-260	CD4+ T cells
ENSG00000159674	1.16E-262	-15.68354356	0.606	0.728	2.32E-259	CD4+ T cells
ENSG00000232629	1.76E-262	-191.7332161	0.861	0.883	3.51E-259	CD4+ T cells
ENSG00000140450	1.15E-260	-1.213843286	0.418	0.592	2.29E-257	CD4+ T cells
ENSG00000116701	3.91E-260	-9.807896023	0.752	0.805	7.83E-257	CD4+ T cells
ENSG00000110077	4.33E-258	-36.72551047	0.792	0.844	8.67E-255	CD4+ T cells
ENSG00000158477	3.56E-256	-75.30229427	0.767	0.797	7.11E-253	CD4+ T cells
ENSG00000242574	6.03E-256	-31.86150188	0.777	0.815	1.21E-252	CD4+ T cells
ENSG00000167642	2.34E-255	-2.132680052	0.696	0.797	4.69E-252	CD4+ T cells
ENSG00000173110	2.04E-252	-97.13870786	0.905	0.92	4.08E-249	CD4+ T cells
ENSG00000160307	1.24E-251	-56.72080883	0.822	0.828	2.48E-248	CD4+ T cells
ENSG00000084234	1.56E-251	-47.03523186	0.745	0.839	3.12E-248	CD4+ T cells
ENSG00000163191	5.75E-251	-80.47046084	0.929	0.957	1.15E-247	CD4+ T cells
ENSG00000204389	1.85E-250	-264.9391539	0.986	0.993	3.70E-247	CD4+ T cells
ENSG00000120129	3.71E-250	-142.9478336	0.904	0.935	7.42E-247	CD4+ T cells
ENSG00000166165	9.73E-249	-12.34043759	0.762	0.835	1.95E-245	CD4+ T cells
ENSG00000166428	6.13E-248	-25.74829815	0.697	0.754	1.23E-244	CD4+ T cells
ENSG00000134028	3.45E-245	-18.23459308	0.591	0.373	6.90E-242	CD4+ T cells
ENSG00000168386	3.66E-244	-14.39837111	0.687	0.759	7.31E-241	CD4+ T cells
ENSG00000165795	1.28E-243	-9.838784413	0.764	0.829	2.57E-240	CD4+ T cells
ENSG00000140379	1.29E-243	-29.38624139	0.659	0.739	2.57E-240	CD4+ T cells
ENSG00000146592	8.77E-241	-2.103535671	0.786	0.811	1.75E-237	CD4+ T cells
ENSG00000010671	8.15E-240	-1.239390479	0.65	0.729	1.63E-236	CD4+ T cells
ENSG00000084207	1.04E-238	-14.39719099	0.842	0.866	2.08E-235	CD4+ T cells
ENSG00000162772	2.63E-237	-45.74181524	0.833	0.867	5.25E-234	CD4+ T cells
ENSG00000173369	2.17E-236	-91.22196658	0.718	0.802	4.34E-233	CD4+ T cells
ENSG00000122861	3.14E-236	-44.07992404	0.804	0.84	6.28E-233	CD4+ T cells
ENSG00000124491	8.78E-236	-28.2031964	0.803	0.824	1.76E-232	CD4+ T cells
ENSG00000184292	7.60E-235	-16.32948326	0.779	0.844	1.52E-231	CD4+ T cells
ENSG00000227507	6.99E-233	-33.7874236	0.906	0.858	1.40E-229	CD4+ T cells
ENSG00000125844	6.80E-232	-18.21733616	0.602	0.707	1.36E-228	CD4+ T cells
ENSG00000258227	8.73E-232	-13.20143212	0.829	0.85	1.75E-228	CD4+ T cells

ENSG00000081041	1.16E-231	-100.9077085	0.787	0.831	2.32E-228	CD4+ T cells
ENSG00000153395	2.07E-230	-1.123433854	0.742	0.796	4.14E-227	CD4+ T cells
ENSG00000119508	1.17E-229	-61.12117774	0.869	0.902	2.35E-226	CD4+ T cells
ENSG00000163220	2.59E-229	-325.8531226	0.792	0.833	5.18E-226	CD4+ T cells
ENSG00000139572	2.97E-229	-11.03255152	0.705	0.743	5.95E-226	CD4+ T cells
ENSG00000128245	3.22E-229	-11.33792535	0.659	0.751	6.43E-226	CD4+ T cells
ENSG00000109861	1.30E-228	-66.0114863	0.715	0.783	2.61E-225	CD4+ T cells
ENSG00000124731	1.41E-227	-8.388235354	0.725	0.779	2.82E-224	CD4+ T cells
ENSG00000119655	1.95E-227	-48.98717009	0.919	0.949	3.90E-224	CD4+ T cells
ENSG00000135838	1.62E-226	-2.317932349	0.394	0.564	3.23E-223	CD4+ T cells
ENSG00000163131	6.57E-226	-150.2039527	0.552	0.677	1.31E-222	CD4+ T cells
ENSG00000100906	3.36E-224	-81.28468171	0.872	0.9	6.71E-221	CD4+ T cells
ENSG00000165168	4.07E-223	-52.08827076	0.687	0.739	8.14E-220	CD4+ T cells
ENSG00000164733	1.88E-218	-404.2176664	0.893	0.928	3.76E-215	CD4+ T cells
ENSG00000211772	1.81E-217	-41.89112498	0.863	0.816	3.62E-214	CD4+ T cells
ENSG00000163661	5.11E-217	-15.61729062	0.537	0.679	1.02E-213	CD4+ T cells
ENSG00000135549	7.30E-217	-2.967745367	0.782	0.812	1.46E-213	CD4+ T cells
ENSG00000182782	1.72E-212	-14.14770844	0.609	0.705	3.44E-209	CD4+ T cells
ENSG00000104312	9.41E-212	-8.440856713	0.695	0.753	1.88E-208	CD4+ T cells
ENSG00000233276	2.19E-211	-34.35090401	0.779	0.834	4.38E-208	CD4+ T cells
ENSG00000118292	4.68E-211	-22.18706642	0.69	0.741	9.36E-208	CD4+ T cells
ENSG00000169504	8.30E-210	-3.067498524	0.644	0.693	1.66E-206	CD4+ T cells
ENSG00000157557	1.31E-209	-13.11915082	0.718	0.776	2.61E-206	CD4+ T cells
ENSG00000172216	2.67E-208	-57.39849013	0.81	0.86	5.34E-205	CD4+ T cells
ENSG00000133789	1.34E-207	-4.249194867	0.671	0.722	2.68E-204	CD4+ T cells
ENSG00000128383	3.26E-207	-47.13536354	0.547	0.673	6.51E-204	CD4+ T cells
ENSG00000164111	2.48E-206	-29.60451469	0.737	0.802	4.96E-203	CD4+ T cells
ENSG00000275302	1.19E-204	-90.8315851	0.828	0.863	2.38E-201	CD4+ T cells
ENSG00000262406	2.01E-204	-115.9766945	0.486	0.575	4.03E-201	CD4+ T cells
ENSG00000087253	3.31E-204	-2.448109388	0.562	0.667	6.62E-201	CD4+ T cells
ENSG00000082397	4.99E-203	-1.720695328	0.673	0.728	9.99E-200	CD4+ T cells
ENSG00000137491	6.76E-203	-19.7428015	0.68	0.749	1.35E-199	CD4+ T cells
ENSG00000142089	1.77E-202	-41.02040646	0.912	0.931	3.55E-199	CD4+ T cells
ENSG00000110079	2.00E-202	-16.87908958	0.655	0.733	4.01E-199	CD4+ T cells
ENSG00000139832	2.56E-202	-7.958002971	0.778	0.824	5.12E-199	CD4+ T cells
ENSG00000166920	6.84E-202	-43.67250299	0.82	0.834	1.37E-198	CD4+ T cells
ENSG00000245532	1.20E-201	-28.51087481	0.673	0.76	2.40E-198	CD4+ T cells
ENSG00000261371	9.01E-201	-9.836105997	0.499	0.614	1.80E-197	CD4+ T cells
ENSG00000170542	2.78E-199	-47.57159317	0.78	0.818	5.57E-196	CD4+ T cells
ENSG00000121316	2.89E-199	-10.14333205	0.667	0.727	5.77E-196	CD4+ T cells
ENSG00000185215	1.91E-196	-44.71011745	0.694	0.751	3.82E-193	CD4+ T cells
ENSG00000169403	3.85E-196	-4.755513758	0.604	0.667	7.69E-193	CD4+ T cells
ENSG00000120738	6.81E-195	-107.4009903	0.838	0.853	1.36E-191	CD4+ T cells
ENSG00000105835	8.84E-195	-49.19737036	0.808	0.83	1.77E-191	CD4+ T cells
ENSG00000110446	3.16E-193	-2.343344098	0.76	0.77	6.33E-190	CD4+ T cells

ENSG00000178719	7.52E-193	-14.62023962	0.709	0.75	1.50E-189	CD4+ T cells
ENSG00000108639	2.87E-191	-27.26213243	0.742	0.792	5.75E-188	CD4+ T cells
ENSG00000203747	4.26E-191	-41.99487155	0.689	0.738	8.52E-188	CD4+ T cells
ENSG00000095303	1.16E-190	-3.110464257	0.624	0.687	2.31E-187	CD4+ T cells
ENSG00000099250	1.30E-189	-1.060489943	0.475	0.628	2.59E-186	CD4+ T cells
ENSG00000182287	3.21E-189	-19.07691935	0.649	0.739	6.41E-186	CD4+ T cells
ENSG00000038427	3.57E-188	-66.18469465	0.636	0.722	7.14E-185	CD4+ T cells
ENSG00000120708	4.58E-188	-49.53496606	0.717	0.795	9.15E-185	CD4+ T cells
ENSG00000172243	9.58E-188	-10.86423004	0.788	0.805	1.92E-184	CD4+ T cells
ENSG00000185022	2.85E-187	-23.88294191	0.617	0.699	5.71E-184	CD4+ T cells
ENSG00000081189	3.02E-187	-4.268662654	0.645	0.679	6.03E-184	CD4+ T cells
ENSG00000168994	8.91E-187	-39.19718058	0.808	0.853	1.78E-183	CD4+ T cells
ENSG00000051108	4.07E-186	-146.85476	0.705	0.773	8.14E-183	CD4+ T cells
ENSG00000130513	1.39E-185	-23.65414563	0.858	0.897	2.78E-182	CD4+ T cells
ENSG00000197253	3.30E-185	-152.7813045	0.866	0.897	6.60E-182	CD4+ T cells
ENSG00000137331	1.06E-184	-16.51644136	0.671	0.715	2.12E-181	CD4+ T cells
ENSG00000131238	1.53E-184	-18.4128626	0.612	0.696	3.06E-181	CD4+ T cells
ENSG00000132965	1.69E-184	-50.19610151	0.787	0.841	3.39E-181	CD4+ T cells
ENSG00000159335	1.75E-184	-3.223173375	0.618	0.702	3.51E-181	CD4+ T cells
ENSG00000132514	4.04E-184	-18.00407836	0.645	0.735	8.08E-181	CD4+ T cells
ENSG00000196923	5.19E-184	-2.049791101	0.563	0.649	1.04E-180	CD4+ T cells
ENSG00000140932	1.44E-183	-4.366855925	0.332	0.51	2.89E-180	CD4+ T cells
ENSG00000162551	5.30E-183	-1.830182417	0.598	0.689	1.06E-179	CD4+ T cells
ENSG00000100368	6.86E-183	-22.93444887	0.785	0.777	1.37E-179	CD4+ T cells
ENSG00000059728	7.53E-180	-24.17466543	0.705	0.761	1.51E-176	CD4+ T cells
ENSG00000078596	1.33E-179	-20.47216885	0.792	0.681	2.66E-176	CD4+ T cells
ENSG00000115355	2.66E-176	-15.06077435	0.59	0.661	5.33E-173	CD4+ T cells
ENSG00000129521	4.49E-176	-26.49322733	0.423	0.584	8.99E-173	CD4+ T cells
ENSG00000115008	8.09E-176	-39.63467565	0.658	0.712	1.62E-172	CD4+ T cells
ENSG00000204257	4.41E-175	-21.83015482	0.659	0.712	8.82E-172	CD4+ T cells
ENSG00000143878	7.83E-175	-223.2027033	0.871	0.886	1.57E-171	CD4+ T cells
ENSG00000251562	2.29E-174	-122.1306053	0.948	0.954	4.58E-171	CD4+ T cells
ENSG00000121797	2.41E-173	-14.74184425	0.683	0.718	4.82E-170	CD4+ T cells
ENSG00000137393	5.32E-173	-1.788847298	0.372	0.55	1.06E-169	CD4+ T cells
ENSG00000180447	5.06E-172	-5.940347507	0.372	0.514	1.01E-168	CD4+ T cells
ENSG00000117154	6.73E-169	-4.114114825	0.495	0.644	1.35E-165	CD4+ T cells
ENSG00000173334	2.09E-168	-33.12915579	0.684	0.741	4.18E-165	CD4+ T cells
ENSG00000018280	2.13E-168	-12.77741449	0.648	0.713	4.25E-165	CD4+ T cells
ENSG00000114013	9.62E-168	-36.16464468	0.781	0.786	1.92E-164	CD4+ T cells
ENSG00000112799	7.21E-167	-3.026260695	0.777	0.793	1.44E-163	CD4+ T cells
ENSG00000162896	3.08E-166	-10.61837391	0.854	0.898	6.16E-163	CD4+ T cells
ENSG00000164400	2.71E-165	-4.036062498	0.619	0.496	5.42E-162	CD4+ T cells
ENSG00000113269	3.11E-165	-8.591679408	0.683	0.731	6.23E-162	CD4+ T cells
ENSG00000118855	1.68E-164	-12.53274368	0.587	0.678	3.37E-161	CD4+ T cells
ENSG00000197956	1.75E-164	-59.68906011	0.93	0.952	3.50E-161	CD4+ T cells

ENSG00000204252	3.27E-164	-12.03463168	0.677	0.72	6.54E-161	CD4+ T cells
ENSG00000135604	5.90E-164	-10.48082947	0.762	0.792	1.18E-160	CD4+ T cells
ENSG00000163683	6.47E-163	-8.950816161	0.493	0.617	1.29E-159	CD4+ T cells
ENSG00000072110	8.72E-163	-6.466539847	0.7	0.765	1.74E-159	CD4+ T cells
ENSG00000125812	2.51E-162	-2.512106855	0.655	0.698	5.02E-159	CD4+ T cells
ENSG00000130775	8.09E-162	-9.457874434	0.611	0.65	1.62E-158	CD4+ T cells
ENSG00000163535	1.13E-160	-4.257574793	0.232	0.405	2.26E-157	CD4+ T cells
ENSG00000088832	4.07E-160	-6.812684544	0.665	0.746	8.14E-157	CD4+ T cells
ENSG00000151726	6.63E-160	-10.20180427	0.604	0.677	1.33E-156	CD4+ T cells
ENSG00000117984	1.94E-159	-108.8675049	0.783	0.826	3.88E-156	CD4+ T cells
ENSG00000182718	1.54E-158	-55.01583807	0.859	0.897	3.07E-155	CD4+ T cells
ENSG00000167601	6.14E-158	-16.98484093	0.761	0.817	1.23E-154	CD4+ T cells
ENSG00000100292	6.49E-158	-162.4000332	0.721	0.74	1.30E-154	CD4+ T cells
ENSG00000127507	9.56E-158	-1.677867329	0.622	0.682	1.91E-154	CD4+ T cells
ENSG00000124882	2.08E-157	-143.2882459	0.741	0.775	4.17E-154	CD4+ T cells
ENSG00000162783	2.80E-157	-49.81631088	0.824	0.86	5.60E-154	CD4+ T cells
ENSG00000131400	2.31E-156	-11.12546825	0.895	0.916	4.63E-153	CD4+ T cells
ENSG00000099860	4.01E-156	-25.14640961	0.837	0.863	8.01E-153	CD4+ T cells
ENSG00000147416	1.19E-155	-22.56805132	0.659	0.727	2.37E-152	CD4+ T cells
ENSG00000148926	2.35E-155	-49.67159009	0.694	0.724	4.69E-152	CD4+ T cells
ENSG00000198435	6.92E-155	-5.534051516	0.711	0.734	1.38E-151	CD4+ T cells
ENSG00000158488	2.43E-154	-18.27687699	0.778	0.787	4.87E-151	CD4+ T cells
ENSG00000100600	5.73E-154	-62.59000708	0.634	0.703	1.15E-150	CD4+ T cells
ENSG00000165140	9.12E-154	-51.37285056	0.715	0.783	1.82E-150	CD4+ T cells
ENSG00000181449	5.35E-153	-16.2723522	0.299	0.434	1.07E-149	CD4+ T cells
ENSG00000118515	1.93E-152	-54.51291759	0.771	0.806	3.86E-149	CD4+ T cells
ENSG00000166825	4.54E-150	-7.577696813	0.747	0.788	9.09E-147	CD4+ T cells
ENSG00000117724	8.50E-150	-18.8038629	0.667	0.513	1.70E-146	CD4+ T cells
ENSG00000160213	1.14E-149	-146.3803929	0.854	0.883	2.28E-146	CD4+ T cells
ENSG00000211445	2.20E-149	-9.8398049	0.76	0.786	4.39E-146	CD4+ T cells
ENSG00000087074	1.03E-147	-58.4834354	0.881	0.892	2.06E-144	CD4+ T cells
ENSG00000111341	3.00E-147	-44.27127975	0.745	0.82	6.01E-144	CD4+ T cells
ENSG00000178789	1.99E-146	-4.338221447	0.26	0.48	3.98E-143	CD4+ T cells
ENSG00000106565	8.34E-146	-27.05181609	0.732	0.78	1.67E-142	CD4+ T cells
ENSG00000111817	3.31E-145	-6.976192399	0.645	0.729	6.63E-142	CD4+ T cells
ENSG00000112096	4.54E-144	-102.2121552	0.852	0.877	9.09E-141	CD4+ T cells
ENSG00000106066	2.28E-142	-66.33065781	0.777	0.791	4.56E-139	CD4+ T cells
ENSG00000105223	6.89E-142	-42.36554601	0.67	0.757	1.38E-138	CD4+ T cells
ENSG00000179348	2.51E-141	-6.181732686	0.656	0.716	5.01E-138	CD4+ T cells
ENSG00000170017	3.05E-141	-9.959198617	0.573	0.663	6.10E-138	CD4+ T cells
ENSG00000167604	3.21E-141	-12.12181503	0.785	0.797	6.41E-138	CD4+ T cells
ENSG00000159399	2.21E-140	-16.7247112	0.757	0.795	4.41E-137	CD4+ T cells
ENSG00000198829	3.92E-140	-1.163144635	0.673	0.711	7.84E-137	CD4+ T cells
ENSG00000163739	4.48E-140	-50.81071472	0.773	0.797	8.96E-137	CD4+ T cells
ENSG00000165997	7.55E-140	-32.11549261	0.696	0.762	1.51E-136	CD4+ T cells

ENSG00000125740	1.86E-139	-127.2096194	0.922	0.935	3.71E-136	CD4+ T cells
ENSG00000161642	3.02E-138	-6.637418182	0.742	0.755	6.05E-135	CD4+ T cells
ENSG00000117318	7.36E-138	-25.39799656	0.684	0.727	1.47E-134	CD4+ T cells
ENSG00000102265	3.69E-135	-43.78442482	0.761	0.793	7.37E-132	CD4+ T cells
ENSG00000114270	5.79E-135	-1.255060017	0.137	0.307	1.16E-131	CD4+ T cells
ENSG00000169136	6.95E-135	-18.80010063	0.635	0.701	1.39E-131	CD4+ T cells
ENSG00000197361	8.60E-135	-1.593307199	0.695	0.556	1.72E-131	CD4+ T cells
ENSG00000181631	5.25E-134	-8.090121286	0.455	0.572	1.05E-130	CD4+ T cells
ENSG00000187164	1.63E-132	-4.475571329	0.684	0.746	3.27E-129	CD4+ T cells
ENSG00000155659	1.09E-131	-14.88269495	0.719	0.751	2.18E-128	CD4+ T cells
ENSG00000101017	4.99E-131	-18.36855804	0.695	0.713	9.98E-128	CD4+ T cells
ENSG00000167613	7.41E-131	-9.110179601	0.739	0.76	1.48E-127	CD4+ T cells
ENSG00000136244	2.71E-130	-44.45206234	0.359	0.49	5.41E-127	CD4+ T cells
ENSG00000107551	1.82E-129	-19.83261563	0.721	0.743	3.64E-126	CD4+ T cells
ENSG00000139370	2.42E-129	-9.391223914	0.65	0.69	4.84E-126	CD4+ T cells
ENSG00000133874	7.69E-129	-1.94754453	0.601	0.658	1.54E-125	CD4+ T cells
ENSG00000122641	2.46E-128	-122.6796384	0.697	0.718	4.92E-125	CD4+ T cells
ENSG00000183486	6.77E-127	-9.156519443	0.676	0.742	1.35E-123	CD4+ T cells
ENSG00000179344	8.70E-127	-33.92716694	0.707	0.729	1.74E-123	CD4+ T cells
ENSG00000160593	9.56E-127	-8.131315773	0.611	0.708	1.91E-123	CD4+ T cells
ENSG00000138623	2.96E-126	-11.29335235	0.688	0.685	5.91E-123	CD4+ T cells
ENSG00000124107	3.77E-126	-29.53395517	0.748	0.817	7.53E-123	CD4+ T cells
ENSG00000130203	9.57E-126	-45.82905481	0.59	0.652	1.91E-122	CD4+ T cells
ENSG00000104951	6.35E-125	-7.535116831	0.633	0.716	1.27E-121	CD4+ T cells
ENSG00000113070	5.27E-124	-186.4592672	0.775	0.825	1.05E-120	CD4+ T cells
ENSG00000075618	7.12E-124	-194.7866456	0.701	0.741	1.42E-120	CD4+ T cells
ENSG00000198682	4.65E-123	-27.34178604	0.751	0.773	9.30E-120	CD4+ T cells
ENSG00000196628	5.51E-122	-13.02224712	0.549	0.607	1.10E-118	CD4+ T cells
ENSG00000114450	5.90E-122	-4.748917953	0.601	0.642	1.18E-118	CD4+ T cells
ENSG00000085063	1.58E-121	-7.289042454	0.704	0.771	3.16E-118	CD4+ T cells
ENSG00000026751	1.70E-121	-34.3862915	0.555	0.629	3.39E-118	CD4+ T cells
ENSG00000102393	2.75E-121	-43.2166589	0.637	0.679	5.50E-118	CD4+ T cells
ENSG00000123975	3.97E-121	-29.46169679	0.741	0.772	7.94E-118	CD4+ T cells
ENSG00000149131	6.07E-121	-37.54568671	0.608	0.669	1.21E-117	CD4+ T cells
ENSG00000134755	1.31E-119	-2.094591938	0.744	0.763	2.62E-116	CD4+ T cells
ENSG00000164265	1.52E-119	-62.99292552	0.869	0.893	3.03E-116	CD4+ T cells
ENSG00000163435	2.74E-119	-3.960511084	0.816	0.863	5.49E-116	CD4+ T cells
ENSG00000138760	2.55E-118	-7.090390549	0.538	0.625	5.09E-115	CD4+ T cells
ENSG00000159189	3.48E-118	-79.06653564	0.827	0.84	6.96E-115	CD4+ T cells
ENSG00000185303	4.34E-118	-17.48966368	0.92	0.944	8.69E-115	CD4+ T cells
ENSG00000181634	3.00E-117	-7.763014202	0.454	0.549	5.99E-114	CD4+ T cells
ENSG00000139970	4.97E-117	-2.208658	0.761	0.762	9.94E-114	CD4+ T cells
ENSG00000087245	7.32E-117	-11.05659807	0.605	0.691	1.46E-113	CD4+ T cells
ENSG00000143322	3.50E-116	-19.95484011	0.658	0.718	7.00E-113	CD4+ T cells
ENSG00000100558	9.70E-115	-1.051172943	0.706	0.765	1.94E-111	CD4+ T cells

ENSG00000148346	4.02E-113	-105.9729919	0.774	0.792	8.05E-110	CD4+ T cells
ENSG00000135047	4.84E-113	-96.57527894	0.714	0.768	9.67E-110	CD4+ T cells
ENSG00000155962	1.23E-112	-4.737102302	0.757	0.758	2.46E-109	CD4+ T cells
ENSG00000042493	1.68E-112	-54.86029452	0.777	0.795	3.36E-109	CD4+ T cells
ENSG00000138166	2.96E-112	-99.16046274	0.783	0.826	5.92E-109	CD4+ T cells
ENSG00000173372	7.63E-112	-49.88885034	0.805	0.826	1.53E-108	CD4+ T cells
ENSG00000131042	1.25E-111	-11.47689378	0.728	0.732	2.50E-108	CD4+ T cells
ENSG00000158050	1.51E-109	-78.22973377	0.856	0.877	3.03E-106	CD4+ T cells
ENSG00000131669	9.88E-109	-16.70366825	0.698	0.747	1.98E-105	CD4+ T cells
ENSG00000087258	1.09E-108	-12.89265099	0.73	0.767	2.18E-105	CD4+ T cells
ENSG00000211751	2.00E-108	-40.88407097	0.739	0.706	4.00E-105	CD4+ T cells
ENSG00000124762	3.46E-108	-71.1146342	0.834	0.86	6.92E-105	CD4+ T cells
ENSG00000189377	5.56E-108	-27.73479612	0.753	0.823	1.11E-104	CD4+ T cells
ENSG00000078081	2.04E-107	-38.55226409	0.708	0.713	4.08E-104	CD4+ T cells
ENSG00000186480	2.63E-107	-168.0351848	0.74	0.768	5.25E-104	CD4+ T cells
ENSG00000092964	3.38E-107	-6.871194852	0.701	0.736	6.75E-104	CD4+ T cells
ENSG00000100097	7.54E-107	-40.34511259	0.84	0.846	1.51E-103	CD4+ T cells
ENSG00000166068	8.72E-107	-3.503516174	0.662	0.701	1.74E-103	CD4+ T cells
ENSG00000179388	4.06E-106	-25.96960369	0.695	0.704	8.12E-103	CD4+ T cells
ENSG00000135678	1.43E-105	-26.0492041	0.568	0.643	2.87E-102	CD4+ T cells
ENSG00000273604	1.72E-105	-22.33914892	0.745	0.723	3.45E-102	CD4+ T cells
ENSG00000060138	1.03E-104	-8.663715925	0.564	0.631	2.06E-101	CD4+ T cells
ENSG00000186074	1.62E-104	-3.165838036	0.743	0.752	3.25E-101	CD4+ T cells
ENSG00000157827	6.01E-104	-1.132911205	0.554	0.61	1.20E-100	CD4+ T cells
ENSG00000275385	9.65E-104	-565.2062512	0.711	0.779	1.93E-100	CD4+ T cells
ENSG00000108924	9.69E-104	-2.375533895	0.694	0.657	1.94E-100	CD4+ T cells
ENSG00000166927	8.23E-103	-32.90917678	0.703	0.736	1.65E-99	CD4+ T cells
ENSG00000272398	8.25E-103	-17.94310999	0.752	0.781	1.65E-99	CD4+ T cells
ENSG00000107798	2.55E-102	-39.9967164	0.52	0.607	5.10E-99	CD4+ T cells
ENSG00000116285	8.46E-102	-16.80571215	0.644	0.712	1.69E-98	CD4+ T cells
ENSG00000112137	1.23E-101	-3.905785741	0.695	0.704	2.47E-98	CD4+ T cells
ENSG00000021355	1.50E-101	-30.23398983	0.671	0.716	2.99E-98	CD4+ T cells
ENSG00000103257	7.74E-101	-53.89695951	0.789	0.819	1.55E-97	CD4+ T cells
ENSG00000068366	1.52E-100	-3.085830711	0.526	0.618	3.04E-97	CD4+ T cells
ENSG00000155966	1.89E-100	-3.450996301	0.544	0.406	3.77E-97	CD4+ T cells
ENSG00000102962	3.52E-100	-86.71735989	0.777	0.818	7.05E-97	CD4+ T cells
ENSG00000129538	2.79E-99	-157.0488637	0.875	0.911	5.59E-96	CD4+ T cells
ENSG00000071246	4.22E-99	-2.316600258	0.663	0.716	8.44E-96	CD4+ T cells
ENSG00000107485	5.15E-99	-12.11617841	0.749	0.717	1.03E-95	CD4+ T cells
ENSG00000125868	2.08E-98	-1.591700585	0.638	0.731	4.16E-95	CD4+ T cells
ENSG00000187474	5.36E-98	-22.08672918	0.693	0.719	1.07E-94	CD4+ T cells
ENSG00000137509	9.06E-98	-2.491221506	0.598	0.65	1.81E-94	CD4+ T cells
ENSG00000136810	2.75E-97	-289.6988061	0.715	0.745	5.50E-94	CD4+ T cells
ENSG00000204866	1.86E-96	-1.44753814	0.576	0.455	3.71E-93	CD4+ T cells
ENSG00000012223	4.51E-96	-487.3729174	0.676	0.731	9.03E-93	CD4+ T cells

ENSG00000144802	5.32E-96	-29.69135984	0.638	0.705	1.06E-92	CD4+ T cells
ENSG00000019169	6.32E-96	-34.66604778	0.716	0.737	1.26E-92	CD4+ T cells
ENSG00000005513	7.64E-95	-6.868502242	0.703	0.576	1.53E-91	CD4+ T cells
ENSG00000255112	1.08E-93	-9.470500663	0.525	0.616	2.16E-90	CD4+ T cells
ENSG00000181649	1.35E-93	-9.137973229	0.727	0.763	2.70E-90	CD4+ T cells
ENSG00000156966	1.65E-93	-3.30038176	0.565	0.647	3.31E-90	CD4+ T cells
ENSG00000062716	1.72E-93	-39.16080902	0.614	0.675	3.43E-90	CD4+ T cells
ENSG00000169245	2.56E-93	-229.4949182	0.756	0.703	5.12E-90	CD4+ T cells
ENSG00000085265	5.63E-93	-48.34756922	0.72	0.741	1.13E-89	CD4+ T cells
ENSG00000106211	6.46E-93	-135.2363318	0.944	0.937	1.29E-89	CD4+ T cells
ENSG00000111144	1.29E-92	-17.60034774	0.686	0.759	2.59E-89	CD4+ T cells
ENSG00000135218	5.97E-92	-6.540952096	0.402	0.538	1.19E-88	CD4+ T cells
ENSG00000165312	6.18E-92	-38.01726379	0.661	0.706	1.24E-88	CD4+ T cells
ENSG00000135916	1.15E-90	-35.72603839	0.438	0.55	2.29E-87	CD4+ T cells
ENSG00000158714	5.47E-90	-4.621853305	0.524	0.593	1.09E-86	CD4+ T cells
ENSG00000079819	9.47E-90	-1.51349777	0.617	0.653	1.89E-86	CD4+ T cells
ENSG00000143416	9.51E-90	-1.004437247	0.831	0.857	1.90E-86	CD4+ T cells
ENSG00000105374	2.03E-89	-21.81294192	0.668	0.74	4.05E-86	CD4+ T cells
ENSG00000178860	4.46E-88	-1.016147753	0.503	0.377	8.92E-85	CD4+ T cells
ENSG00000111912	8.73E-88	-7.457309125	0.721	0.797	1.75E-84	CD4+ T cells
ENSG00000175899	1.03E-87	-9.370179986	0.615	0.68	2.06E-84	CD4+ T cells
ENSG00000116774	3.31E-87	-23.14423662	0.682	0.74	6.62E-84	CD4+ T cells
ENSG00000196562	4.56E-87	-1.908668033	0.648	0.677	9.12E-84	CD4+ T cells
ENSG00000116016	4.62E-87	-3.517876884	0.596	0.658	9.24E-84	CD4+ T cells
ENSG00000134531	1.05E-86	-30.96062021	0.656	0.738	2.11E-83	CD4+ T cells
ENSG00000158270	2.17E-86	-11.20863907	0.682	0.747	4.34E-83	CD4+ T cells
ENSG00000108688	2.66E-85	-56.17974164	0.667	0.546	5.32E-82	CD4+ T cells
ENSG00000134460	4.31E-85	-22.08054646	0.698	0.574	8.61E-82	CD4+ T cells
ENSG00000165029	5.31E-85	-7.019528671	0.472	0.558	1.06E-81	CD4+ T cells
ENSG00000120885	9.51E-85	29.90583804	0.695	0.773	1.90E-81	CD4+ T cells
ENSG00000143153	1.76E-84	-10.97739098	0.791	0.817	3.51E-81	CD4+ T cells
ENSG00000038945	2.17E-84	-29.47126577	0.677	0.703	4.34E-81	CD4+ T cells
ENSG00000117115	2.62E-84	-52.27366975	0.846	0.836	5.25E-81	CD4+ T cells
ENSG00000157404	4.97E-84	3.161327336	0.608	0.674	9.93E-81	CD4+ T cells
ENSG00000134539	6.39E-84	-5.51383467	0.574	0.655	1.28E-80	CD4+ T cells
ENSG00000115414	1.70E-83	-79.81017493	0.62	0.664	3.40E-80	CD4+ T cells
ENSG00000165949	1.84E-83	-83.92450527	0.891	0.895	3.68E-80	CD4+ T cells
ENSG00000229644	1.97E-83	-13.61920248	0.478	0.562	3.94E-80	CD4+ T cells
ENSG00000123095	1.24E-82	-5.226786386	0.425	0.5	2.48E-79	CD4+ T cells
ENSG00000183023	1.59E-82	-2.063792152	0.806	0.79	3.18E-79	CD4+ T cells
ENSG00000112394	2.52E-82	-15.93009993	0.731	0.759	5.04E-79	CD4+ T cells
ENSG00000183779	5.91E-82	-5.197268507	0.602	0.663	1.18E-78	CD4+ T cells
ENSG00000122852	7.88E-82	-17.88794699	0.929	0.947	1.58E-78	CD4+ T cells
ENSG00000115919	2.27E-81	-5.522050882	0.667	0.671	4.55E-78	CD4+ T cells
ENSG00000116191	2.42E-81	-2.497735155	0.549	0.613	4.84E-78	CD4+ T cells

ENSG00000169413	3.17E-81	-12.23651678	0.677	0.67	6.34E-78	CD4+ T cells
ENSG00000275395	6.82E-81	-79.1117329	0.65	0.698	1.36E-77	CD4+ T cells
ENSG00000115590	9.20E-81	-8.334998271	0.594	0.636	1.84E-77	CD4+ T cells
ENSG00000169908	1.19E-80	-6.183417931	0.82	0.843	2.37E-77	CD4+ T cells
ENSG00000069849	2.39E-80	-55.25663019	0.756	0.78	4.78E-77	CD4+ T cells
ENSG00000112759	1.01E-79	-8.96539299	0.661	0.693	2.03E-76	CD4+ T cells
ENSG00000265972	1.39E-79	-57.34715676	0.753	0.772	2.78E-76	CD4+ T cells
ENSG00000178381	4.21E-79	-52.17810983	0.755	0.77	8.42E-76	CD4+ T cells
ENSG00000130830	5.69E-79	-1.697723078	0.66	0.689	1.14E-75	CD4+ T cells
ENSG00000198959	6.04E-79	-14.31749904	0.604	0.662	1.21E-75	CD4+ T cells
ENSG00000197093	1.14E-78	-1.05725371	0.509	0.598	2.28E-75	CD4+ T cells
ENSG00000183019	2.77E-78	-33.06725469	0.725	0.741	5.54E-75	CD4+ T cells
ENSG00000171236	3.34E-78	-15.77460453	0.718	0.735	6.68E-75	CD4+ T cells
ENSG00000130208	1.66E-77	-175.1127878	0.702	0.752	3.32E-74	CD4+ T cells
ENSG00000150687	1.76E-77	-1.183992885	0.52	0.581	3.53E-74	CD4+ T cells
ENSG00000099985	1.82E-77	-41.72921914	0.801	0.81	3.63E-74	CD4+ T cells
ENSG00000196230	1.96E-77	-34.35933242	0.805	0.829	3.91E-74	CD4+ T cells
ENSG00000149534	4.68E-77	-4.857105009	0.664	0.704	9.36E-74	CD4+ T cells
ENSG00000260428	2.51E-76	-12.74604963	0.692	0.582	5.02E-73	CD4+ T cells
ENSG00000100985	2.28E-75	-362.4078683	0.599	0.642	4.57E-72	CD4+ T cells
ENSG00000211897	2.95E-75	#NAME?	0.574	0.633	5.91E-72	CD4+ T cells
ENSG00000109805	3.30E-75	-2.660052085	0.391	0.484	6.60E-72	CD4+ T cells
ENSG00000117036	4.30E-74	-18.29764404	0.66	0.738	8.60E-71	CD4+ T cells
ENSG00000157227	5.71E-74	-11.27693342	0.67	0.716	1.14E-70	CD4+ T cells
ENSG00000124216	6.14E-74	-3.166391841	0.793	0.776	1.23E-70	CD4+ T cells
ENSG00000148773	7.31E-74	-32.07398458	0.535	0.619	1.46E-70	CD4+ T cells
ENSG00000172236	1.36E-73	-47.94437287	0.868	0.864	2.72E-70	CD4+ T cells
ENSG00000175040	1.47E-73	-9.351404216	0.619	0.628	2.94E-70	CD4+ T cells
ENSG00000109099	2.74E-73	-14.84725489	0.783	0.779	5.48E-70	CD4+ T cells
ENSG00000072694	4.61E-73	-34.90519441	0.636	0.652	9.23E-70	CD4+ T cells
ENSG00000090104	1.07E-72	-88.74197959	0.706	0.778	2.14E-69	CD4+ T cells
ENSG00000071205	1.20E-72	-1.393499922	0.585	0.645	2.40E-69	CD4+ T cells
ENSG00000125657	1.23E-72	-17.74838117	0.625	0.671	2.46E-69	CD4+ T cells
ENSG00000172215	1.01E-71	-3.788238827	0.674	0.618	2.03E-68	CD4+ T cells
ENSG00000129757	3.33E-71	-10.0809157	0.577	0.628	6.65E-68	CD4+ T cells
ENSG00000064989	4.29E-71	-1.004297611	0.365	0.435	8.59E-68	CD4+ T cells
ENSG00000042980	5.04E-70	-3.620694046	0.586	0.595	1.01E-66	CD4+ T cells
ENSG00000122966	3.60E-69	-1.826361905	0.718	0.763	7.20E-66	CD4+ T cells
ENSG00000108691	1.10E-68	-174.6177085	0.793	0.8	2.20E-65	CD4+ T cells
ENSG00000010278	3.56E-68	-13.26143551	0.679	0.708	7.11E-65	CD4+ T cells
ENSG00000159388	1.37E-67	-121.0105514	0.906	0.897	2.74E-64	CD4+ T cells
ENSG00000111252	3.51E-66	-8.794068728	0.705	0.722	7.03E-63	CD4+ T cells
ENSG00000074416	3.74E-66	-5.699544085	0.799	0.696	7.48E-63	CD4+ T cells
ENSG00000163599	6.69E-66	-44.75489558	0.691	0.612	1.34E-62	CD4+ T cells
ENSG00000152492	8.51E-66	-10.32311399	0.509	0.581	1.70E-62	CD4+ T cells

ENSG00000141543	2.28E-65	-46.01925277	0.77	0.788	4.56E-62	CD4+ T cells
ENSG00000167618	2.82E-65	-1.666337557	0.808	0.728	5.64E-62	CD4+ T cells
ENSG00000204103	6.59E-65	-41.19446218	0.756	0.755	1.32E-61	CD4+ T cells
ENSG00000103569	3.16E-64	-16.49323024	0.741	0.71	6.32E-61	CD4+ T cells
ENSG00000151929	3.90E-64	-74.75786166	0.774	0.783	7.80E-61	CD4+ T cells
ENSG00000169756	9.40E-64	-23.74277621	0.684	0.729	1.88E-60	CD4+ T cells
ENSG00000138678	2.15E-63	-20.79037169	0.645	0.676	4.30E-60	CD4+ T cells
ENSG00000226979	3.88E-63	-3.608686242	0.66	0.575	7.75E-60	CD4+ T cells
ENSG00000151012	4.46E-63	-5.819790889	0.624	0.609	8.92E-60	CD4+ T cells
ENSG00000114541	6.19E-63	-3.012034331	0.801	0.744	1.24E-59	CD4+ T cells
ENSG00000180879	7.09E-63	-108.4440991	0.723	0.769	1.42E-59	CD4+ T cells
ENSG00000175130	5.43E-62	-44.70824942	0.609	0.663	1.09E-58	CD4+ T cells
ENSG00000171617	6.23E-62	-13.26495801	0.601	0.638	1.25E-58	CD4+ T cells
ENSG00000177575	4.28E-61	-17.6556745	0.6	0.644	8.57E-58	CD4+ T cells
ENSG00000108679	4.46E-61	-16.59534633	0.824	0.845	8.91E-58	CD4+ T cells
ENSG00000142192	1.21E-60	-26.74832599	0.755	0.805	2.42E-57	CD4+ T cells
ENSG00000196743	1.26E-60	-19.72547405	0.654	0.652	2.52E-57	CD4+ T cells
ENSG00000153064	1.65E-60	-2.63377945	0.3	0.437	3.31E-57	CD4+ T cells
ENSG00000162711	1.80E-60	-15.96820199	0.655	0.693	3.59E-57	CD4+ T cells
ENSG00000123342	2.10E-60	-58.57024888	0.801	0.806	4.20E-57	CD4+ T cells
ENSG00000102524	3.11E-60	-2.191970882	0.604	0.652	6.23E-57	CD4+ T cells
ENSG00000145414	8.34E-60	-29.47160445	0.603	0.66	1.67E-56	CD4+ T cells
ENSG00000180758	2.35E-59	-25.24133764	0.641	0.656	4.70E-56	CD4+ T cells
ENSG00000180644	3.87E-59	-19.41027286	0.596	0.69	7.75E-56	CD4+ T cells
ENSG00000276600	4.07E-59	-2.059720005	0.665	0.717	8.14E-56	CD4+ T cells
ENSG00000143110	4.09E-59	-6.018194905	0.772	0.79	8.18E-56	CD4+ T cells
ENSG00000176014	5.78E-59	-4.39968911	0.758	0.747	1.16E-55	CD4+ T cells
ENSG00000152766	1.02E-58	-12.4487281	0.781	0.741	2.05E-55	CD4+ T cells
ENSG00000149485	2.02E-58	-2.852909081	0.321	0.446	4.04E-55	CD4+ T cells
ENSG00000026025	3.00E-58	-192.8703862	0.951	0.948	6.01E-55	CD4+ T cells
ENSG00000187479	6.25E-58	-6.825137767	0.626	0.663	1.25E-54	CD4+ T cells
ENSG00000211679	6.47E-58	#NAME?	0.587	0.634	1.29E-54	CD4+ T cells
ENSG00000126353	2.96E-57	-90.77259729	0.66	0.618	5.93E-54	CD4+ T cells
ENSG00000159200	9.07E-57	-21.96987002	0.473	0.552	1.81E-53	CD4+ T cells
ENSG00000138135	9.45E-57	-18.31651756	0.663	0.69	1.89E-53	CD4+ T cells
ENSG00000150045	1.22E-56	-2.910437711	0.723	0.74	2.45E-53	CD4+ T cells
ENSG00000118785	1.69E-56	-810.3102017	0.406	0.487	3.38E-53	CD4+ T cells
ENSG00000197629	7.45E-56	-10.9695595	0.655	0.657	1.49E-52	CD4+ T cells
ENSG00000105825	8.80E-56	-23.85631054	0.466	0.515	1.76E-52	CD4+ T cells
ENSG00000141682	5.13E-55	-16.30859713	0.631	0.653	1.03E-51	CD4+ T cells
ENSG00000132205	2.33E-54	-2.572446922	0.648	0.666	4.66E-51	CD4+ T cells
ENSG00000124145	4.09E-54	-13.5049199	0.818	0.838	8.17E-51	CD4+ T cells
ENSG00000167748	4.62E-54	-25.84524276	0.263	0.345	9.24E-51	CD4+ T cells
ENSG00000109321	4.80E-54	-52.10337153	0.66	0.709	9.60E-51	CD4+ T cells
ENSG00000178573	1.10E-53	-19.14255211	0.765	0.745	2.21E-50	CD4+ T cells

ENSG00000131724	1.94E-53	-8.792570669	0.75	0.74	3.88E-50	CD4+ T cells
ENSG00000153071	2.25E-53	-9.69514834	0.723	0.72	4.50E-50	CD4+ T cells
ENSG00000197766	2.27E-53	-26.23144193	0.769	0.775	4.54E-50	CD4+ T cells
ENSG00000198719	5.61E-53	-2.677962509	0.698	0.556	1.12E-49	CD4+ T cells
ENSG00000113742	5.94E-53	-22.76734014	0.543	0.589	1.19E-49	CD4+ T cells
ENSG00000106541	6.58E-53	-13.58007503	0.578	0.628	1.32E-49	CD4+ T cells
ENSG00000164692	6.62E-53	31.79625589	0.513	0.563	1.32E-49	CD4+ T cells
ENSG00000147454	8.89E-53	-17.36440313	0.658	0.675	1.78E-49	CD4+ T cells
ENSG00000127954	1.07E-52	-7.097956487	0.793	0.793	2.14E-49	CD4+ T cells
ENSG00000163359	1.11E-52	-1.255020751	0.392	0.493	2.23E-49	CD4+ T cells
ENSG00000085719	1.57E-52	-13.99262915	0.556	0.618	3.13E-49	CD4+ T cells
ENSG00000129993	3.11E-52	-1.643893776	0.646	0.638	6.22E-49	CD4+ T cells
ENSG00000176907	4.84E-52	-5.658071528	0.687	0.698	9.69E-49	CD4+ T cells
ENSG00000198502	1.07E-51	-63.3506741	0.378	0.478	2.15E-48	CD4+ T cells
ENSG00000211598	1.36E-51	-696.3319417	0.225	0.329	2.71E-48	CD4+ T cells
ENSG00000169242	1.89E-51	-2.743139891	0.859	0.871	3.78E-48	CD4+ T cells
ENSG00000117322	2.05E-51	5.070290793	0.383	0.285	4.10E-48	CD4+ T cells
ENSG00000125843	3.77E-51	1.304699602	0.567	0.59	7.55E-48	CD4+ T cells
ENSG00000145632	4.66E-51	-15.60709103	0.713	0.735	9.33E-48	CD4+ T cells
ENSG00000138744	6.99E-51	-11.97715166	0.614	0.656	1.40E-47	CD4+ T cells
ENSG00000173114	1.98E-50	-5.284572598	0.561	0.504	3.95E-47	CD4+ T cells
ENSG00000184060	3.38E-50	-1.677879727	0.706	0.666	6.75E-47	CD4+ T cells
ENSG00000104267	4.69E-50	-1.564433954	0.73	0.728	9.39E-47	CD4+ T cells
ENSG00000119922	6.22E-50	-57.54924288	0.647	0.64	1.24E-46	CD4+ T cells
ENSG00000101347	1.17E-49	-28.54255395	0.736	0.767	2.33E-46	CD4+ T cells
ENSG00000166523	1.55E-49	-6.234246616	0.258	0.404	3.11E-46	CD4+ T cells
ENSG00000184371	2.72E-49	-19.23662767	0.82	0.768	5.44E-46	CD4+ T cells
ENSG00000129244	9.21E-49	-15.77430418	0.589	0.668	1.84E-45	CD4+ T cells
ENSG00000173848	1.18E-48	-18.18784579	0.683	0.698	2.36E-45	CD4+ T cells
ENSG00000136383	3.12E-48	-1.727438389	0.727	0.694	6.24E-45	CD4+ T cells
ENSG00000173451	4.15E-47	-3.610028193	0.624	0.641	8.30E-44	CD4+ T cells
ENSG00000163734	6.93E-47	-97.12473522	0.768	0.765	1.39E-43	CD4+ T cells
ENSG00000102575	2.00E-46	-167.8358038	0.65	0.666	4.01E-43	CD4+ T cells
ENSG00000116717	3.52E-46	8.287675698	0.669	0.728	7.05E-43	CD4+ T cells
ENSG00000176890	4.04E-46	-7.00593926	0.594	0.626	8.07E-43	CD4+ T cells
ENSG00000178695	8.09E-46	-17.20307815	0.682	0.677	1.62E-42	CD4+ T cells
ENSG00000234883	1.07E-45	-1.767747004	0.66	0.653	2.13E-42	CD4+ T cells
ENSG00000116133	1.17E-45	-2.896450818	0.719	0.745	2.33E-42	CD4+ T cells
ENSG00000174460	1.28E-45	8.238958035	0.348	0.434	2.55E-42	CD4+ T cells
ENSG00000148053	1.56E-45	-2.201351968	0.681	0.6	3.11E-42	CD4+ T cells
ENSG00000131981	2.01E-45	-118.5435624	0.734	0.761	4.01E-42	CD4+ T cells
ENSG00000126432	2.11E-45	-2.194841016	0.784	0.799	4.22E-42	CD4+ T cells
ENSG00000135046	2.73E-45	-60.26400175	0.834	0.84	5.45E-42	CD4+ T cells
ENSG00000104738	4.86E-45	-5.496308817	0.657	0.651	9.71E-42	CD4+ T cells
ENSG00000179841	1.02E-44	-2.318786107	0.634	0.511	2.03E-41	CD4+ T cells

ENSG00000060982	1.30E-44	-1.919677374	0.645	0.669	2.59E-41	CD4+ T cells
ENSG00000204305	1.79E-44	-14.81933719	0.522	0.591	3.57E-41	CD4+ T cells
ENSG00000117632	1.90E-44	-30.98969069	0.693	0.707	3.81E-41	CD4+ T cells
ENSG00000153823	2.52E-44	-1.587166214	0.793	0.766	5.03E-41	CD4+ T cells
ENSG00000135929	7.27E-44	-1.978401775	0.616	0.646	1.45E-40	CD4+ T cells
ENSG00000168675	1.06E-43	-20.31398504	0.553	0.602	2.11E-40	CD4+ T cells
ENSG00000145287	3.07E-43	-30.90745052	0.672	0.72	6.14E-40	CD4+ T cells
ENSG00000137959	7.44E-43	-9.799656027	0.708	0.697	1.49E-39	CD4+ T cells
ENSG00000067082	1.04E-42	-109.2558929	0.88	0.894	2.09E-39	CD4+ T cells
ENSG00000163751	1.27E-42	-12.33394862	0.706	0.719	2.53E-39	CD4+ T cells
ENSG00000114251	1.57E-42	-3.649321347	0.661	0.679	3.14E-39	CD4+ T cells
ENSG00000135480	1.74E-42	-2.135949735	0.779	0.793	3.48E-39	CD4+ T cells
ENSG00000111537	3.75E-42	-80.49532333	0.496	0.566	7.51E-39	CD4+ T cells
ENSG00000102804	3.82E-42	-14.53252333	0.535	0.58	7.64E-39	CD4+ T cells
ENSG00000147614	4.51E-42	-5.366499406	0.211	0.344	9.02E-39	CD4+ T cells
ENSG00000102755	4.75E-42	-10.96371069	0.709	0.702	9.50E-39	CD4+ T cells
ENSG00000230006	6.07E-42	-1.745471142	0.382	0.345	1.21E-38	CD4+ T cells
ENSG00000019991	7.41E-42	-1.958678475	0.7	0.682	1.48E-38	CD4+ T cells
ENSG00000111424	8.29E-42	-8.401004377	0.725	0.718	1.66E-38	CD4+ T cells
ENSG00000139318	9.68E-42	-15.11345552	0.634	0.685	1.94E-38	CD4+ T cells
ENSG00000186283	1.02E-41	-10.00438191	0.653	0.65	2.04E-38	CD4+ T cells
ENSG00000187608	1.04E-41	-11.88246025	0.728	0.707	2.08E-38	CD4+ T cells
ENSG00000163508	1.11E-41	-6.442906228	0.548	0.584	2.21E-38	CD4+ T cells
ENSG00000182985	1.25E-41	-2.397792366	0.679	0.709	2.51E-38	CD4+ T cells
ENSG00000143603	1.41E-41	-5.170277968	0.37	0.448	2.82E-38	CD4+ T cells
ENSG00000157601	1.48E-41	-10.11700942	0.802	0.777	2.96E-38	CD4+ T cells
ENSG00000121769	2.15E-41	-3.502023987	0.384	0.478	4.29E-38	CD4+ T cells
ENSG00000185291	2.29E-41	-6.460985925	0.574	0.599	4.57E-38	CD4+ T cells
ENSG00000088827	3.05E-41	-12.63416909	0.803	0.794	6.10E-38	CD4+ T cells
ENSG00000137502	3.82E-41	-27.84500998	0.58	0.49	7.64E-38	CD4+ T cells
ENSG00000153201	1.02E-40	-35.09147493	0.676	0.72	2.04E-37	CD4+ T cells
ENSG00000141655	1.15E-40	-1.226118898	0.696	0.688	2.29E-37	CD4+ T cells
ENSG00000168243	2.23E-40	-20.68460149	0.473	0.394	4.47E-37	CD4+ T cells
ENSG00000115738	2.93E-40	-50.97817359	0.784	0.804	5.86E-37	CD4+ T cells
ENSG00000099958	5.77E-40	-90.99896749	0.375	0.467	1.15E-36	CD4+ T cells
ENSG00000013588	9.35E-40	-3.320396906	0.776	0.808	1.87E-36	CD4+ T cells
ENSG00000164949	1.06E-39	-2.581134455	0.708	0.722	2.13E-36	CD4+ T cells
ENSG00000162692	1.55E-39	-1.449209929	0.665	0.535	3.10E-36	CD4+ T cells
ENSG00000109320	1.89E-39	-22.12239516	0.75	0.771	3.78E-36	CD4+ T cells
ENSG00000182010	5.71E-39	-1.440298952	0.552	0.494	1.14E-35	CD4+ T cells
ENSG00000211890	8.77E-38	#NAME?	0.382	0.458	1.75E-34	CD4+ T cells
ENSG00000105967	9.80E-38	-6.378783692	0.533	0.566	1.96E-34	CD4+ T cells
ENSG00000091972	1.77E-37	-4.086031284	0.587	0.474	3.55E-34	CD4+ T cells
ENSG00000188820	2.71E-37	-2.245778136	0.612	0.597	5.42E-34	CD4+ T cells
ENSG00000100234	5.40E-37	-3.433769007	0.704	0.723	1.08E-33	CD4+ T cells

ENSG00000138185	7.28E-37	-5.311838356	0.738	0.695	1.46E-33	CD4+ T cells
ENSG00000145824	7.61E-37	-1.360054103	0.727	0.732	1.52E-33	CD4+ T cells
ENSG00000156127	6.65E-36	-7.781946703	0.77	0.714	1.33E-32	CD4+ T cells
ENSG00000185507	6.90E-36	-21.18730274	0.716	0.715	1.38E-32	CD4+ T cells
ENSG00000114315	8.53E-36	-24.72947809	0.686	0.685	1.71E-32	CD4+ T cells
ENSG00000154237	1.75E-35	-2.223928428	0.687	0.686	3.49E-32	CD4+ T cells
ENSG00000166598	1.78E-35	-297.8571634	0.697	0.712	3.57E-32	CD4+ T cells
ENSG00000093072	2.55E-35	-16.68330826	0.643	0.654	5.11E-32	CD4+ T cells
ENSG00000166562	2.84E-35	-33.75194424	0.701	0.721	5.68E-32	CD4+ T cells
ENSG00000132386	5.10E-35	-12.65391976	0.688	0.684	1.02E-31	CD4+ T cells
ENSG00000106236	1.81E-34	-9.920327048	0.19	0.283	3.62E-31	CD4+ T cells
ENSG00000170113	2.62E-34	-5.103069417	0.679	0.581	5.24E-31	CD4+ T cells
ENSG00000188404	3.35E-34	-8.644583819	0.804	0.725	6.69E-31	CD4+ T cells
ENSG00000186827	3.57E-34	-3.946406912	0.691	0.634	7.14E-31	CD4+ T cells
ENSG00000135625	5.65E-34	-18.35604782	0.492	0.53	1.13E-30	CD4+ T cells
ENSG00000044574	8.66E-34	-171.4852908	0.801	0.827	1.73E-30	CD4+ T cells
ENSG00000143184	1.78E-33	15.77000215	0.649	0.55	3.57E-30	CD4+ T cells
ENSG00000243466	2.00E-33	#NAME?	0.33	0.428	3.99E-30	CD4+ T cells
ENSG00000099194	3.39E-33	-20.43424499	0.567	0.591	6.77E-30	CD4+ T cells
ENSG00000072274	3.80E-33	-62.07980359	0.673	0.691	7.60E-30	CD4+ T cells
ENSG00000165457	5.94E-33	-11.41943046	0.763	0.759	1.19E-29	CD4+ T cells
ENSG00000185112	6.26E-33	-12.1664331	0.632	0.653	1.25E-29	CD4+ T cells
ENSG00000115523	1.06E-32	-81.51198941	0.73	0.742	2.12E-29	CD4+ T cells
ENSG00000089041	1.32E-32	-4.722584665	0.624	0.616	2.65E-29	CD4+ T cells
ENSG00000165434	2.23E-32	-19.10784802	0.664	0.615	4.47E-29	CD4+ T cells
ENSG00000184489	6.44E-32	-55.33850294	0.486	0.543	1.29E-28	CD4+ T cells
ENSG00000170265	1.01E-31	-3.919320361	0.661	0.605	2.02E-28	CD4+ T cells
ENSG00000149212	4.36E-31	-16.01828021	0.632	0.57	8.72E-28	CD4+ T cells
ENSG00000104093	8.26E-31	-1.367845704	0.38	0.45	1.65E-27	CD4+ T cells
ENSG00000138061	1.13E-30	-8.832010278	0.587	0.6	2.26E-27	CD4+ T cells
ENSG00000119900	1.13E-30	-8.196217068	0.671	0.648	2.26E-27	CD4+ T cells
ENSG00000151914	2.52E-30	-2.15485053	0.706	0.719	5.05E-27	CD4+ T cells
ENSG00000064042	3.03E-30	-1.844188702	0.641	0.577	6.07E-27	CD4+ T cells
ENSG00000155657	1.77E-29	-5.885911011	0.521	0.466	3.55E-26	CD4+ T cells
ENSG00000176083	1.92E-29	-7.131950489	0.451	0.357	3.84E-26	CD4+ T cells
ENSG00000160856	2.29E-29	-5.562264334	0.649	0.662	4.58E-26	CD4+ T cells
ENSG00000213949	2.47E-29	3.461595131	0.36	0.427	4.93E-26	CD4+ T cells
ENSG00000100979	2.93E-29	-8.0226577	0.584	0.586	5.86E-26	CD4+ T cells
ENSG00000240403	4.37E-29	-1.307368185	0.4	0.426	8.74E-26	CD4+ T cells
ENSG00000211899	8.16E-29	#NAME?	0.452	0.508	1.63E-25	CD4+ T cells
ENSG00000100453	8.49E-29	-161.290836	0.635	0.641	1.70E-25	CD4+ T cells
ENSG00000198574	2.25E-28	-4.54998482	0.669	0.685	4.50E-25	CD4+ T cells
ENSG00000270164	5.49E-28	-3.475644443	0.34	0.312	1.10E-24	CD4+ T cells
ENSG00000105369	5.95E-28	-90.46746171	0.49	0.514	1.19E-24	CD4+ T cells
ENSG00000138433	3.32E-27	-113.0111315	0.453	0.516	6.65E-24	CD4+ T cells

ENSG00000150782	3.56E-27	-6.570613902	0.68	0.644	7.13E-24	CD4+ T cells
ENSG00000071282	4.07E-27	-1.001502143	0.631	0.654	8.13E-24	CD4+ T cells
ENSG00000137441	8.35E-27	-14.94220151	0.75	0.736	1.67E-23	CD4+ T cells
ENSG00000128918	9.17E-27	-7.257832208	0.377	0.37	1.83E-23	CD4+ T cells
ENSG00000166592	9.78E-27	-23.33753825	0.837	0.839	1.96E-23	CD4+ T cells
ENSG00000132906	1.31E-26	-4.380718374	0.656	0.658	2.62E-23	CD4+ T cells
ENSG00000136235	1.35E-26	-95.60965728	0.53	0.552	2.69E-23	CD4+ T cells
ENSG00000123384	1.41E-26	-2.909984252	0.738	0.714	2.82E-23	CD4+ T cells
ENSG00000164825	1.65E-26	-12.87925423	0.679	0.669	3.30E-23	CD4+ T cells
ENSG00000211896	7.40E-26	#NAME?	0.59	0.602	1.48E-22	CD4+ T cells
ENSG00000124772	9.72E-26	-4.771433197	0.29	0.378	1.94E-22	CD4+ T cells
ENSG00000102760	1.34E-25	-39.05106311	0.842	0.805	2.69E-22	CD4+ T cells
ENSG00000146070	1.39E-25	-6.45215324	0.492	0.517	2.78E-22	CD4+ T cells
ENSG00000122877	2.04E-25	-29.23623237	0.778	0.74	4.08E-22	CD4+ T cells
ENSG00000023445	3.52E-25	-127.9246606	0.8	0.763	7.05E-22	CD4+ T cells
ENSG00000048462	8.79E-25	-7.916472064	0.171	0.255	1.76E-21	CD4+ T cells
ENSG00000123131	1.84E-24	-14.93908158	0.478	0.518	3.67E-21	CD4+ T cells
ENSG00000100450	2.96E-24	-9.716236118	0.55	0.577	5.93E-21	CD4+ T cells
ENSG00000119862	3.37E-24	-5.902138107	0.713	0.633	6.75E-21	CD4+ T cells
ENSG00000124766	7.10E-24	-63.6939544	0.784	0.772	1.42E-20	CD4+ T cells
ENSG00000115884	7.35E-24	-14.66766207	0.754	0.766	1.47E-20	CD4+ T cells
ENSG00000172322	2.45E-23	-2.582721212	0.761	0.724	4.90E-20	CD4+ T cells
ENSG00000197461	2.49E-23	-1.002790821	0.728	0.673	4.98E-20	CD4+ T cells
ENSG00000088826	3.90E-23	-2.015731532	0.605	0.544	7.79E-20	CD4+ T cells
ENSG00000163687	4.47E-23	-1.047951398	0.204	0.322	8.95E-20	CD4+ T cells
ENSG00000082438	4.91E-23	-1.537007235	0.486	0.518	9.82E-20	CD4+ T cells
ENSG00000181374	1.69E-22	-69.91195807	0.781	0.774	3.39E-19	CD4+ T cells
ENSG00000165092	2.85E-22	-30.38082754	0.636	0.627	5.70E-19	CD4+ T cells
ENSG00000184557	4.17E-22	-124.0036325	0.873	0.866	8.35E-19	CD4+ T cells
ENSG00000205189	4.84E-22	-23.20833242	0.731	0.65	9.69E-19	CD4+ T cells
ENSG00000127951	5.72E-22	-24.44908979	0.796	0.763	1.14E-18	CD4+ T cells
ENSG00000108700	6.00E-22	-117.103712	0.388	0.436	1.20E-18	CD4+ T cells
ENSG00000108821	6.16E-22	55.10514436	0.712	0.733	1.23E-18	CD4+ T cells
ENSG00000095970	6.71E-22	-3.416707906	0.708	0.678	1.34E-18	CD4+ T cells
ENSG00000125148	1.05E-21	-145.0524149	0.86	0.855	2.10E-18	CD4+ T cells
ENSG00000154277	1.37E-21	-3.401160292	0.507	0.425	2.74E-18	CD4+ T cells
ENSG00000141753	1.40E-21	-3.563483656	0.682	0.646	2.80E-18	CD4+ T cells
ENSG00000155850	1.44E-21	-1.220335523	0.526	0.553	2.88E-18	CD4+ T cells
ENSG00000169194	3.00E-21	-23.7584156	0.419	0.487	5.99E-18	CD4+ T cells
ENSG00000131747	3.71E-21	-30.4730697	0.618	0.524	7.43E-18	CD4+ T cells
ENSG00000175445	5.62E-21	-43.11520141	0.68	0.671	1.12E-17	CD4+ T cells
ENSG00000066294	7.18E-21	-18.45702495	0.565	0.614	1.44E-17	CD4+ T cells
ENSG00000163823	1.11E-20	-5.727768348	0.714	0.659	2.23E-17	CD4+ T cells
ENSG00000133048	1.79E-20	-191.536049	0.449	0.476	3.57E-17	CD4+ T cells
ENSG00000125735	2.05E-20	-3.020874583	0.78	0.715	4.11E-17	CD4+ T cells

ENSG00000144476	2.10E-20	-3.246593007	0.455	0.48	4.20E-17	CD4+ T cells
ENSG00000066279	2.79E-20	-28.80184755	0.262	0.318	5.59E-17	CD4+ T cells
ENSG00000135451	4.13E-20	-1.413071052	0.3	0.303	8.25E-17	CD4+ T cells
ENSG00000143013	4.59E-20	-2.517495674	0.671	0.676	9.17E-17	CD4+ T cells
ENSG00000140287	6.77E-20	-1.700604688	0.758	0.659	1.35E-16	CD4+ T cells
ENSG00000128965	9.98E-20	-11.80630998	0.723	0.651	2.00E-16	CD4+ T cells
ENSG00000166963	2.70E-19	-7.573875709	0.69	0.616	5.40E-16	CD4+ T cells
ENSG00000087085	3.59E-19	-2.426427113	0.597	0.593	7.18E-16	CD4+ T cells
ENSG00000123700	3.63E-19	-1.281098676	0.485	0.433	7.25E-16	CD4+ T cells
ENSG00000170476	7.60E-19	-94.68561102	0.4	0.436	1.52E-15	CD4+ T cells
ENSG00000105697	1.65E-18	-13.15664657	0.613	0.547	3.31E-15	CD4+ T cells
ENSG00000111796	1.85E-18	-2.291216235	0.733	0.773	3.69E-15	CD4+ T cells
ENSG00000013374	2.24E-18	-19.05373299	0.523	0.557	4.48E-15	CD4+ T cells
ENSG00000142583	2.60E-18	-4.566627774	0.551	0.54	5.21E-15	CD4+ T cells
ENSG00000137807	1.20E-17	-2.009931887	0.482	0.525	2.40E-14	CD4+ T cells
ENSG00000118193	1.37E-17	-4.807213087	0.525	0.537	2.75E-14	CD4+ T cells
ENSG00000143297	1.80E-17	-6.991735223	0.294	0.396	3.60E-14	CD4+ T cells
ENSG00000010610	2.37E-17	-21.76992423	0.816	0.857	4.74E-14	CD4+ T cells
ENSG00000054219	4.27E-17	-9.511301338	0.657	0.666	8.53E-14	CD4+ T cells
ENSG00000211753	5.45E-17	-2.601007229	0.439	0.326	1.09E-13	CD4+ T cells
ENSG00000112303	7.79E-17	-1.9164738	0.603	0.567	1.56E-13	CD4+ T cells
ENSG00000124191	1.14E-16	1.277189595	0.486	0.552	2.29E-13	CD4+ T cells
ENSG00000136111	1.23E-16	-40.30933242	0.65	0.631	2.45E-13	CD4+ T cells
ENSG00000179163	1.29E-16	-10.32887491	0.592	0.61	2.57E-13	CD4+ T cells
ENSG00000117215	3.06E-16	-5.301551528	0.337	0.269	6.11E-13	CD4+ T cells
ENSG00000145012	3.86E-16	-24.07888352	0.585	0.603	7.71E-13	CD4+ T cells
ENSG00000138755	4.24E-16	-223.4622018	0.486	0.417	8.48E-13	CD4+ T cells
ENSG00000140403	8.58E-16	-31.06093026	0.659	0.661	1.72E-12	CD4+ T cells
ENSG00000129116	1.18E-15	-2.801807433	0.69	0.643	2.36E-12	CD4+ T cells
ENSG00000186417	1.44E-15	-1.804936007	0.402	0.449	2.87E-12	CD4+ T cells
ENSG00000170323	2.51E-15	-36.06306958	0.382	0.44	5.02E-12	CD4+ T cells
ENSG00000164047	3.40E-15	-83.83230707	0.77	0.74	6.79E-12	CD4+ T cells
ENSG00000030419	3.71E-15	-5.811412975	0.625	0.576	7.43E-12	CD4+ T cells
ENSG00000171848	1.60E-14	-2.090912739	0.504	0.435	3.20E-11	CD4+ T cells
ENSG00000163993	1.77E-14	-2.554153469	0.453	0.493	3.55E-11	CD4+ T cells
ENSG00000269404	2.04E-14	-8.049821302	0.769	0.698	4.08E-11	CD4+ T cells
ENSG00000169715	4.84E-14	1.510630624	0.694	0.62	9.68E-11	CD4+ T cells
ENSG00000122254	5.17E-14	-10.54570731	0.278	0.353	1.03E-10	CD4+ T cells
ENSG00000142156	8.17E-14	-10.23739628	0.451	0.477	1.63E-10	CD4+ T cells
ENSG00000109943	1.11E-13	-70.13393962	0.391	0.444	2.21E-10	CD4+ T cells
ENSG00000106537	2.74E-13	-6.534704082	0.677	0.664	5.47E-10	CD4+ T cells
ENSG00000088325	2.89E-13	-5.743891153	0.646	0.653	5.79E-10	CD4+ T cells
ENSG00000169508	2.94E-13	-168.7867526	0.777	0.823	5.88E-10	CD4+ T cells
ENSG00000155660	3.05E-13	-15.87164641	0.576	0.594	6.09E-10	CD4+ T cells
ENSG00000113140	3.77E-13	14.31934882	0.569	0.601	7.54E-10	CD4+ T cells

ENSG00000158825	5.50E-13	-1.366367735	0.713	0.671	1.10E-09	CD4+ T cells
ENSG00000169385	5.56E-13	-15.78407644	0.706	0.705	1.11E-09	CD4+ T cells
ENSG00000162998	7.17E-13	-1.021189125	0.56	0.468	1.43E-09	CD4+ T cells
ENSG00000115963	7.19E-13	-45.17860979	0.677	0.657	1.44E-09	CD4+ T cells
ENSG00000118257	1.01E-12	-1.64798327	0.638	0.571	2.02E-09	CD4+ T cells
ENSG00000089692	3.74E-12	-13.25256946	0.584	0.532	7.48E-09	CD4+ T cells
ENSG00000162512	4.00E-12	-2.597701669	0.691	0.667	8.00E-09	CD4+ T cells
ENSG00000049249	2.09E-11	-22.91232918	0.493	0.529	4.19E-08	CD4+ T cells
ENSG00000170442	3.48E-11	-8.95403566	0.571	0.494	6.96E-08	CD4+ T cells
ENSG00000123411	4.16E-11	-17.22225228	0.688	0.656	8.32E-08	CD4+ T cells
ENSG00000050405	4.21E-11	-10.17384109	0.554	0.604	8.43E-08	CD4+ T cells
ENSG00000007312	5.13E-11	-7.073088217	0.462	0.484	1.03E-07	CD4+ T cells
ENSG00000242265	6.24E-11	-26.80323415	0.465	0.487	1.25E-07	CD4+ T cells
ENSG00000110092	6.31E-11	-10.4895035	0.629	0.608	1.26E-07	CD4+ T cells
ENSG00000006451	1.29E-10	-13.81481036	0.67	0.68	2.58E-07	CD4+ T cells
ENSG00000003147	1.50E-10	-4.179640032	0.621	0.644	2.99E-07	CD4+ T cells
ENSG00000170684	1.58E-10	-2.891886265	0.647	0.603	3.16E-07	CD4+ T cells
ENSG00000156738	1.84E-10	-59.07683091	0.328	0.366	3.67E-07	CD4+ T cells
ENSG00000128218	2.08E-10	-5.064574118	0.29	0.375	4.16E-07	CD4+ T cells
ENSG00000105246	2.91E-10	-7.492084599	0.656	0.611	5.82E-07	CD4+ T cells
ENSG00000183688	6.79E-10	-1.308039256	0.523	0.533	1.36E-06	CD4+ T cells
ENSG00000256235	6.90E-10	-2.862754388	0.788	0.769	1.38E-06	CD4+ T cells
ENSG00000198833	7.98E-10	-18.15487701	0.625	0.618	1.60E-06	CD4+ T cells
ENSG00000186081	1.18E-09	-99.22323188	0.198	0.263	2.36E-06	CD4+ T cells
ENSG00000122035	1.39E-09	-1.815335799	0.606	0.569	2.79E-06	CD4+ T cells
ENSG00000115009	1.43E-09	-213.4250596	0.788	0.784	2.85E-06	CD4+ T cells
ENSG00000068796	1.55E-09	-12.62793588	0.471	0.505	3.10E-06	CD4+ T cells
ENSG00000144959	1.67E-09	-2.59216041	0.594	0.585	3.34E-06	CD4+ T cells
ENSG00000145685	2.00E-09	-3.830853275	0.747	0.713	4.00E-06	CD4+ T cells
ENSG00000097021	2.12E-09	-5.378094904	0.62	0.618	4.24E-06	CD4+ T cells
ENSG00000211898	2.36E-09	#NAME?	0.296	0.341	4.73E-06	CD4+ T cells
ENSG00000196092	2.39E-09	-3.789231679	0.331	0.292	4.79E-06	CD4+ T cells
ENSG00000057657	4.93E-09	-36.325899	0.723	0.713	9.86E-06	CD4+ T cells
ENSG00000176046	5.44E-09	-7.06725266	0.591	0.61	1.09E-05	CD4+ T cells
ENSG00000211593	7.37E-09	-1.705643126	0.169	0.272	1.47E-05	CD4+ T cells
ENSG00000166033	1.15E-08	-8.326098215	0.498	0.504	2.30E-05	CD4+ T cells
ENSG00000121858	1.22E-08	-7.689360917	0.542	0.556	2.44E-05	CD4+ T cells
ENSG00000101773	1.28E-08	-3.914969452	0.596	0.575	2.56E-05	CD4+ T cells
ENSG00000159166	1.39E-08	-4.806500735	0.729	0.729	2.78E-05	CD4+ T cells
ENSG00000196154	1.95E-08	-42.70913521	0.927	0.926	3.90E-05	CD4+ T cells
ENSG00000211893	2.82E-08	#NAME?	0.338	0.336	5.63E-05	CD4+ T cells
ENSG00000166278	3.05E-08	-3.947114067	0.748	0.682	6.10E-05	CD4+ T cells
ENSG00000004799	3.39E-08	-4.094724849	0.415	0.467	6.78E-05	CD4+ T cells
ENSG00000211895	4.34E-08	#NAME?	0.465	0.488	8.67E-05	CD4+ T cells
ENSG00000110848	4.53E-08	-31.89271305	0.802	0.759	9.05E-05	CD4+ T cells

ENSG00000120875	5.50E-08	-39.95379255	0.685	0.711	0.000110057	CD4+ T cells
ENSG00000111536	6.79E-08	-3.185838968	0.407	0.405	0.000135768	CD4+ T cells
ENSG00000132744	8.34E-08	-8.826636271	0.716	0.653	0.000166726	CD4+ T cells
ENSG00000156299	8.50E-08	-14.5537224	0.655	0.629	0.000169937	CD4+ T cells
ENSG00000188505	1.06E-07	-5.537713006	0.506	0.498	0.000212892	CD4+ T cells
ENSG00000132465	1.60E-07	#NAME?	0.459	0.483	0.000320257	CD4+ T cells
ENSG00000137804	2.87E-07	-3.574234034	0.444	0.484	0.000574409	CD4+ T cells
ENSG00000269821	3.18E-07	-4.926324455	0.71	0.633	0.00063509	CD4+ T cells
ENSG00000149257	3.48E-07	-32.13283696	0.834	0.791	0.000696293	CD4+ T cells
ENSG00000132170	4.02E-07	-18.74654908	0.699	0.64	0.000803893	CD4+ T cells
ENSG00000142173	4.77E-07	-6.258614976	0.554	0.556	0.000953341	CD4+ T cells
ENSG00000086300	5.20E-07	-45.31286317	0.565	0.553	0.00104032	CD4+ T cells
ENSG00000117586	5.32E-07	-14.33228173	0.651	0.603	0.001063671	CD4+ T cells
ENSG00000135919	9.67E-07	-2.028502621	0.595	0.548	0.001933809	CD4+ T cells
ENSG00000121933	1.08E-06	-1.690270969	0.598	0.55	0.002158434	CD4+ T cells
ENSG00000187193	1.19E-06	4.452599618	0.751	0.718	0.002382387	CD4+ T cells
ENSG00000161638	1.60E-06	-4.303342324	0.664	0.658	0.003194703	CD4+ T cells
ENSG00000203668	1.71E-06	-3.522811337	0.575	0.511	0.003424406	CD4+ T cells
ENSG00000186407	1.92E-06	-13.34116933	0.813	0.733	0.003837257	CD4+ T cells
ENSG00000117560	1.94E-06	-4.336605443	0.666	0.64	0.003886372	CD4+ T cells
ENSG00000158321	2.53E-06	-4.571221881	0.597	0.605	0.005052811	CD4+ T cells
ENSG00000177374	2.97E-06	-8.697517769	0.672	0.622	0.005939534	CD4+ T cells
ENSG00000127824	3.13E-06	-7.319397794	0.714	0.67	0.006259193	CD4+ T cells
ENSG00000102445	3.46E-06	-7.464360311	0.477	0.472	0.006929773	CD4+ T cells
ENSG00000091409	4.82E-06	-14.57926584	0.545	0.551	0.009636407	CD4+ T cells
ENSG00000125898	4.91E-06	-4.595940711	0.649	0.624	0.009813815	CD4+ T cells
ENSG00000167600	6.69E-06	-3.904284973	0.783	0.706	0.013373876	CD4+ T cells
ENSG00000147872	6.85E-06	-67.53270003	0.728	0.711	0.01369633	CD4+ T cells
ENSG00000168542	7.84E-06	45.45490432	0.613	0.61	0.015688001	CD4+ T cells
ENSG00000122224	1.20E-05	-55.91208697	0.472	0.488	0.024055722	CD4+ T cells
ENSG00000186818	1.27E-05	-8.838049542	0.591	0.517	0.025452198	CD4+ T cells
ENSG00000144290	1.53E-05	-5.548416095	0.384	0.375	0.030649767	CD4+ T cells
ENSG00000211689	1.57E-05	-4.899078758	0.6	0.554	0.031492741	CD4+ T cells
ENSG00000106733	2.31E-05	-11.22987279	0.56	0.606	0.046106506	CD4+ T cells
ENSG00000143333	2.48E-05	-27.4605577	0.747	0.755	0.049553373	CD4+ T cells
ENSG00000243772	0	2.829767052	0.8	0.671	0	NKT cells
ENSG00000271503	0	-18.59806363	0.761	0.68	0	NKT cells
ENSG00000221957	1.24E-272	6.978125272	0.738	0.585	2.48E-269	NKT cells
ENSG00000145649	4.71E-223	5.506642225	0.682	0.578	9.41E-220	NKT cells
ENSG00000125498	1.22E-198	7.309404308	0.679	0.575	2.43E-195	NKT cells
ENSG00000136960	2.49E-154	-3.508899804	0.508	0.627	4.98E-151	NKT cells
ENSG00000160789	3.56E-143	-34.15466881	0.822	0.869	7.11E-140	NKT cells
ENSG00000012124	4.33E-136	-10.30469795	0.366	0.506	8.65E-133	NKT cells
ENSG00000123610	4.53E-128	-3.233742767	0.707	0.568	9.05E-125	NKT cells
ENSG00000184226	6.26E-127	-2.026047578	0.379	0.488	1.25E-123	NKT cells

ENSG00000170345	2.19E-112	-224.1780819	0.925	0.94	4.38E-109	NKT cells
ENSG00000196352	1.04E-111	-21.24384588	0.889	0.888	2.07E-108	NKT cells
ENSG00000157765	1.33E-105	-1.387022289	0.872	0.863	2.66E-102	NKT cells
ENSG00000177706	2.96E-92	-10.85894862	0.704	0.547	5.92E-89	NKT cells
ENSG00000125144	9.08E-85	-22.23696631	0.714	0.605	1.82E-81	NKT cells
ENSG00000105711	1.29E-83	-1.778112231	0.776	0.669	2.58E-80	NKT cells
ENSG00000049768	3.06E-70	-16.28159782	0.671	0.7	6.12E-67	NKT cells
ENSG00000137265	5.09E-69	-23.5528073	0.565	0.669	1.02E-65	NKT cells
ENSG00000143387	3.03E-68	-119.9047487	0.597	0.484	6.06E-65	NKT cells
ENSG00000196611	3.02E-60	-13.89243541	0.241	0.345	6.04E-57	NKT cells
ENSG00000135094	7.29E-54	-55.27991726	0.74	0.716	1.46E-50	NKT cells
ENSG00000092969	4.71E-50	-1.926218531	0.53	0.597	9.42E-47	NKT cells
ENSG00000107317	2.77E-46	-109.2587213	0.587	0.494	5.54E-43	NKT cells
ENSG00000134152	6.66E-45	-5.202400668	0.565	0.47	1.33E-41	NKT cells
ENSG00000089199	8.44E-45	-42.94803506	0.635	0.581	1.69E-41	NKT cells
ENSG00000189013	1.90E-43	1.411393737	0.522	0.384	3.80E-40	NKT cells
ENSG00000028277	2.96E-43	-7.982567478	0.586	0.627	5.92E-40	NKT cells
ENSG00000131459	4.95E-43	-1.708331352	0.519	0.423	9.90E-40	NKT cells
ENSG00000143185	1.23E-42	-31.97150168	0.676	0.71	2.45E-39	NKT cells
ENSG00000174944	9.02E-39	-2.079484628	0.598	0.612	1.80E-35	NKT cells
ENSG00000138449	1.69E-37	-16.84281239	0.635	0.545	3.38E-34	NKT cells
ENSG00000165272	1.82E-37	-16.18985628	0.821	0.817	3.64E-34	NKT cells
ENSG00000182853	5.65E-36	-6.63401613	0.664	0.674	1.13E-32	NKT cells
ENSG00000102096	7.02E-36	-113.6477176	0.785	0.796	1.40E-32	NKT cells
ENSG00000087586	5.24E-34	-4.199540034	0.479	0.521	1.05E-30	NKT cells
ENSG00000198576	1.33E-33	-8.234709393	0.618	0.662	2.67E-30	NKT cells
ENSG00000070190	1.22E-30	-9.777516416	0.598	0.613	2.45E-27	NKT cells
ENSG00000232810	4.91E-30	-46.92312972	0.8	0.738	9.83E-27	NKT cells
ENSG00000115602	6.08E-30	-14.58772436	0.715	0.624	1.22E-26	NKT cells
ENSG00000131016	7.47E-29	-6.674454943	0.518	0.546	1.49E-25	NKT cells
ENSG00000137801	1.05E-28	-84.22717963	0.656	0.708	2.11E-25	NKT cells
ENSG00000137571	3.73E-27	-1.711980758	0.599	0.53	7.46E-24	NKT cells
ENSG00000227191	4.97E-27	-1.236191421	0.631	0.641	9.95E-24	NKT cells
ENSG00000138778	6.01E-25	-19.48345262	0.408	0.444	1.20E-21	NKT cells
ENSG00000135378	3.93E-24	-3.908344379	0.642	0.658	7.85E-21	NKT cells
ENSG00000164920	4.07E-23	-14.3196459	0.378	0.399	8.15E-20	NKT cells
ENSG00000197696	3.84E-20	-3.344768867	0.668	0.628	7.69E-17	NKT cells
ENSG00000035720	4.28E-20	-14.13068439	0.389	0.427	8.57E-17	NKT cells
ENSG00000181847	4.60E-20	-14.65536766	0.613	0.591	9.19E-17	NKT cells
ENSG00000105609	3.08E-19	-2.633575378	0.687	0.573	6.17E-16	NKT cells
ENSG00000133063	2.19E-17	-38.55573745	0.338	0.396	4.39E-14	NKT cells
ENSG00000073849	7.59E-17	-10.37384328	0.7	0.62	1.52E-13	NKT cells
ENSG00000164236	1.21E-16	-4.538643138	0.637	0.609	2.42E-13	NKT cells
ENSG00000163735	3.54E-16	-16.84422151	0.368	0.415	7.08E-13	NKT cells
ENSG00000198734	1.65E-15	-1.099491348	0.582	0.572	3.29E-12	NKT cells

ENSG00000147889	1.35E-14	-5.442064855	0.601	0.627	2.71E-11	NKT cells
ENSG00000172602	1.40E-14	-4.411393486	0.73	0.717	2.80E-11	NKT cells
ENSG00000196549	5.59E-14	-1.311929896	0.444	0.48	1.12E-10	NKT cells
ENSG00000104419	8.24E-14	-10.09394348	0.747	0.765	1.65E-10	NKT cells
ENSG00000150337	1.38E-13	-1.265444605	0.8	0.699	2.77E-10	NKT cells
ENSG00000134321	1.46E-10	-13.08838608	0.598	0.639	2.92E-07	NKT cells
ENSG00000117228	1.93E-10	-36.17453577	0.456	0.498	3.87E-07	NKT cells
ENSG00000131203	1.98E-10	-9.073444934	0.752	0.681	3.96E-07	NKT cells
ENSG00000121039	5.88E-10	-11.29651855	0.595	0.633	1.18E-06	NKT cells
ENSG00000271614	6.88E-10	-1.887832054	0.631	0.623	1.38E-06	NKT cells
ENSG00000139289	6.89E-10	-1.897870634	0.761	0.793	1.38E-06	NKT cells
ENSG00000177606	1.08E-09	-139.630325	0.93	0.949	2.16E-06	NKT cells
ENSG00000066827	4.76E-09	-8.241998117	0.666	0.651	9.52E-06	NKT cells
ENSG00000106366	9.19E-09	-22.57667727	0.675	0.629	1.84E-05	NKT cells
ENSG00000187601	1.42E-08	-2.085039266	0.662	0.636	2.85E-05	NKT cells
ENSG00000163132	1.64E-08	-24.9540421	0.486	0.398	3.29E-05	NKT cells
ENSG00000056736	6.79E-08	-1.803972711	0.739	0.728	0.00013586	NKT cells
ENSG00000123989	9.46E-08	-3.475019769	0.515	0.473	0.000189219	NKT cells
ENSG00000215218	9.75E-08	-1.873215682	0.304	0.219	0.000194912	NKT cells
ENSG00000124466	3.84E-07	-34.47651056	0.669	0.637	0.000767121	NKT cells
ENSG00000138821	7.17E-07	-3.191087089	0.616	0.602	0.001434231	NKT cells
ENSG00000073737	1.06E-06	-1.425657108	0.519	0.466	0.002116077	NKT cells
ENSG00000119917	1.32E-06	-48.88362005	0.721	0.711	0.002649677	NKT cells
ENSG00000088340	1.83E-05	-10.73660343	0.375	0.311	0.0366017	NKT cells
ENSG00000138180	3.03E-268	1.219254688	0.714	0.504	6.05E-265	B cells
ENSG00000163808	5.57E-251	1.734260856	0.191	0.432	1.11E-247	B cells
ENSG00000109684	9.28E-232	1.252350757	0.731	0.66	1.86E-228	B cells
ENSG00000112742	6.02E-171	1.063143606	0.272	0.478	1.20E-167	B cells
ENSG00000247746	1.26E-149	1.429994729	0.692	0.517	2.52E-146	B cells
ENSG00000143228	3.87E-148	1.057848241	0.669	0.426	7.74E-145	B cells
ENSG00000108551	5.29E-139	-3.349812017	0.648	0.746	1.06E-135	B cells
ENSG00000126787	2.50E-137	6.515753467	0.439	0.153	4.99E-134	B cells
ENSG00000136026	5.91E-132	-16.44500613	0.433	0.65	1.18E-128	B cells
ENSG00000123485	1.06E-120	1.774149363	0.503	0.336	2.12E-117	B cells
ENSG00000170312	4.86E-94	1.727072568	0.561	0.637	9.73E-91	B cells
ENSG00000136573	1.60E-92	2.74392977	0.6	0.497	3.19E-89	B cells
ENSG00000105974	2.34E-88	-8.325189308	0.684	0.598	4.68E-85	B cells
ENSG00000168685	6.05E-76	-2.210249897	0.694	0.772	1.21E-72	B cells
ENSG00000110944	1.14E-73	-28.80990483	0.517	0.65	2.27E-70	B cells
ENSG00000138160	2.76E-70	1.985429469	0.534	0.407	5.53E-67	B cells
ENSG00000211892	2.31E-58	#NAME?	0.59	0.466	4.61E-55	B cells
ENSG00000068489	2.13E-57	1.17795898	0.256	0.369	4.26E-54	B cells
ENSG00000023330	6.42E-57	-4.885884013	0.481	0.626	1.28E-53	B cells
ENSG00000239951	3.65E-52	-424.7866414	0.479	0.367	7.30E-49	B cells
ENSG00000132185	4.36E-49	1.113425886	0.418	0.241	8.72E-46	B cells

ENSG00000072571	2.82E-48	1.134953143	0.585	0.527	5.64E-45	B cells
ENSG00000189057	9.45E-47	2.493701219	0.442	0.592	1.89E-43	B cells
ENSG00000127074	1.17E-46	6.866784711	0.48	0.629	2.35E-43	B cells
ENSG00000134285	1.61E-46	-4.298666154	0.416	0.518	3.22E-43	B cells
ENSG00000138207	8.88E-46	-4.203329859	0.406	0.5	1.78E-42	B cells
ENSG00000128656	4.02E-45	1.043419718	0.627	0.42	8.04E-42	B cells
ENSG00000110777	2.73E-44	-9.526899213	0.362	0.424	5.45E-41	B cells
ENSG00000117707	1.64E-39	1.67347703	0.548	0.795	3.27E-36	B cells
ENSG00000175063	1.69E-38	1.716110755	0.638	0.519	3.39E-35	B cells
ENSG00000050730	2.60E-29	1.899500795	0.565	0.39	5.21E-26	B cells
ENSG00000152804	8.04E-28	1.545852476	0.601	0.51	1.61E-24	B cells
ENSG00000115594	1.62E-27	1.021140098	0.601	0.7	3.23E-24	B cells
ENSG00000134545	1.18E-25	1.901946433	0.479	0.512	2.36E-22	B cells
ENSG00000211677	3.17E-24	#NAME?	0.516	0.404	6.35E-21	B cells
ENSG00000137507	2.27E-23	12.04001967	0.654	0.6	4.54E-20	B cells
ENSG00000234184	4.55E-23	1.682357253	0.368	0.111	9.11E-20	B cells
ENSG00000204381	1.01E-19	1.203075681	0.486	0.588	2.02E-16	B cells
ENSG00000183722	6.71E-19	1.763079829	0.546	0.419	1.34E-15	B cells
ENSG00000106123	2.17E-18	1.728358634	0.592	0.58	4.34E-15	B cells
ENSG00000153563	7.02E-17	-5.25561218	0.26	0.404	1.40E-13	B cells
ENSG00000122952	1.98E-14	1.007328317	0.655	0.669	3.97E-11	B cells
ENSG00000170509	7.83E-14	1.356422158	0.428	0.5	1.57E-10	B cells
ENSG00000137474	3.91E-13	1.679919347	0.479	0.454	7.81E-10	B cells
ENSG00000108932	4.79E-11	-9.113471411	0.594	0.512	9.58E-08	B cells
ENSG00000135114	6.22E-11	25.2709122	0.628	0.586	1.24E-07	B cells
ENSG00000164938	7.09E-11	-37.85065927	0.587	0.583	1.42E-07	B cells
ENSG00000075218	5.18E-10	1.144121087	0.391	0.413	1.04E-06	B cells
ENSG00000226777	1.62E-09	-13.75218802	0.381	0.248	3.24E-06	B cells
ENSG00000169439	6.38E-09	-6.69108732	0.629	0.611	1.28E-05	B cells
ENSG00000130164	2.88E-08	-1.900735876	0.76	0.647	5.77E-05	B cells
ENSG00000164056	8.52E-08	1.180101115	0.58	0.612	0.000170376	B cells
ENSG00000115758	2.94E-07	-21.65221078	0.637	0.619	0.000588539	B cells
ENSG00000197632	5.31E-07	-90.15717645	0.36	0.405	0.00106275	B cells
ENSG00000089685	1.92E-05	1.232544957	0.668	0.682	0.038302396	B cells
ENSG00000205089	0	1.073216944	0.962	0.528	0	B cells
ENSG00000116748	7.85E-263	1.056549258	0.813	0.096	1.57E-259	B cells
ENSG00000107438	9.11E-174	1.119801551	0.901	0.658	1.82E-170	B cells
ENSG00000171241	4.89E-140	2.056287069	0.199	0.464	9.78E-137	B cells
ENSG00000149054	5.42E-128	1.4129624	0.201	0.442	1.08E-124	B cells
ENSG00000116014	5.28E-122	1.552030427	0.237	0.561	1.06E-118	B cells
ENSG00000110492	2.50E-120	1.524185135	0.804	0.736	5.00E-117	B cells
ENSG00000189143	4.29E-113	1.467728257	0.862	0.715	8.59E-110	B cells
ENSG00000123096	1.47E-98	1.910648999	0.725	0.302	2.95E-95	B cells
ENSG00000164330	2.93E-93	2.803351462	0.253	0.279	5.87E-90	B cells
ENSG00000154099	3.39E-65	3.598579959	0.7	0.501	6.77E-62	B cells

ENSG00000164440	1.39E-63	1.447705057	0.263	0.524	2.78E-60	B cells
ENSG00000181690	9.95E-60	2.257600139	0.285	0.524	1.99E-56	B cells
ENSG00000077943	1.39E-45	1.697911416	0.637	0.171	2.78E-42	B cells
ENSG00000154553	5.53E-35	2.088254508	0.709	0.706	1.11E-31	B cells
ENSG00000211949	6.06E-35	101.1989596	0.349	0.154	1.21E-31	B cells
ENSG00000108405	2.57E-33	1.030398141	0.368	0.559	5.14E-30	B cells
ENSG00000103089	2.71E-31	1.054965825	0.36	0.385	5.42E-28	B cells
ENSG00000183091	6.55E-31	2.479504275	0.304	0.424	1.31E-27	B cells
ENSG00000241351	8.96E-27	410.159272	0.35	0.153	1.79E-23	B cells
ENSG00000138411	3.12E-26	1.362974386	0.606	0.383	6.24E-23	B cells
ENSG00000168952	1.49E-16	3.603747921	0.604	0.624	2.97E-13	B cells
ENSG00000125398	2.06E-15	4.313820234	0.513	0.714	4.12E-12	B cells
ENSG00000205038	1.88E-06	1.377044385	0.443	0.593	0.003768003	B cells
ENSG00000168081	6.96E-06	2.317909492	0.535	0.202	0.013914825	B cells
ENSG00000135363	0	2.69687187	0.778	0.563	0	monocytic cells
ENSG00000161055	0	2.58113169	0.881	0.863	0	monocytic cells
ENSG00000107719	0	2.42641002	0.719	0.645	0	monocytic cells
ENSG00000163106	0	2.30481413	0.76	0.727	0	monocytic cells
ENSG00000090530	0	2.018429409	0.751	0.687	0	monocytic cells
ENSG00000183578	0	1.791374049	0.727	0.744	0	monocytic cells
ENSG00000106991	0	1.513585918	0.703	0.63	0	monocytic cells
ENSG00000164604	0	1.176484175	0.638	0.433	0	monocytic cells
ENSG00000183087	5.26E-297	1.165379406	0.691	0.581	1.05E-293	monocytic cells
ENSG00000117009	4.09E-291	2.395883394	0.635	0.436	8.18E-288	monocytic cells
ENSG00000105509	8.10E-256	2.676912635	0.598	0.346	1.62E-252	monocytic cells
ENSG00000105472	4.14E-250	2.34024877	0.709	0.757	8.27E-247	monocytic cells
ENSG00000158716	3.72E-239	1.67597514	0.718	0.675	7.44E-236	monocytic cells
ENSG00000165685	5.85E-237	2.758008338	0.696	0.731	1.17E-233	monocytic cells
ENSG00000154783	2.25E-229	1.013146141	0.666	0.637	4.50E-226	monocytic cells
ENSG00000149573	3.63E-228	2.10956981	0.708	0.665	7.26E-225	monocytic cells
ENSG00000106034	2.27E-224	1.38093079	0.616	0.436	4.53E-221	monocytic cells
ENSG00000257017	6.91E-223	1.366907849	0.508	0.304	1.38E-219	monocytic cells
ENSG00000237649	3.81E-218	2.498796798	0.695	0.695	7.63E-215	monocytic cells
ENSG00000163453	1.25E-214	1.156532749	0.731	0.742	2.50E-211	monocytic cells
ENSG00000169116	1.77E-211	1.138420264	0.726	0.761	3.55E-208	monocytic cells
ENSG00000123838	1.73E-204	1.16118695	0.79	0.865	3.45E-201	monocytic cells
ENSG00000183742	1.66E-188	1.156671938	0.681	0.726	3.32E-185	monocytic cells
ENSG00000154146	4.82E-175	2.228217856	0.71	0.796	9.63E-172	monocytic cells
ENSG00000137672	1.83E-173	1.593276797	0.665	0.708	3.65E-170	monocytic cells
ENSG00000087076	2.36E-173	1.636202278	0.579	0.393	4.73E-170	monocytic cells
ENSG00000111186	2.46E-173	1.146082485	0.43	0.209	4.91E-170	monocytic cells
ENSG00000150540	3.48E-172	2.760094074	0.661	0.689	6.97E-169	monocytic cells
ENSG00000142512	7.08E-171	2.232798228	0.649	0.653	1.42E-167	monocytic cells
ENSG00000118849	4.80E-164	2.677245088	0.524	0.28	9.61E-161	monocytic cells
ENSG00000101335	3.87E-159	1.392672959	0.664	0.582	7.73E-156	monocytic cells

ENSG00000159450	1.73E-152	2.204140818	0.592	0.541	3.47E-149	monocytic cells
ENSG00000156113	8.42E-152	1.013366924	0.625	0.606	1.68E-148	monocytic cells
ENSG00000091436	2.83E-149	1.427233711	0.624	0.573	5.66E-146	monocytic cells
ENSG00000139567	5.17E-147	2.104072497	0.606	0.571	1.03E-143	monocytic cells
ENSG00000101443	1.74E-144	1.104525132	0.765	0.8	3.47E-141	monocytic cells
ENSG00000144837	2.17E-143	2.116753132	0.625	0.633	4.34E-140	monocytic cells
ENSG00000196260	7.57E-143	1.175102292	0.751	0.804	1.51E-139	monocytic cells
ENSG00000135052	4.88E-142	1.00005226	0.675	0.673	9.77E-139	monocytic cells
ENSG00000139618	8.78E-137	1.82428024	0.578	0.421	1.76E-133	monocytic cells
ENSG00000143320	1.32E-130	1.331050526	0.732	0.802	2.65E-127	monocytic cells
ENSG00000157240	3.82E-120	1.141873314	0.624	0.647	7.64E-117	monocytic cells
ENSG00000186642	3.56E-118	1.412735421	0.632	0.715	7.12E-115	monocytic cells
ENSG00000175352	1.45E-113	1.405996332	0.597	0.593	2.90E-110	monocytic cells
ENSG00000214894	1.04E-108	1.585888182	0.501	0.384	2.07E-105	monocytic cells
ENSG00000079257	2.04E-107	1.317015174	0.289	0.601	4.08E-104	monocytic cells
ENSG00000128283	8.62E-104	2.022278604	0.729	0.818	1.72E-100	monocytic cells
ENSG00000180113	1.48E-102	1.072014568	0.444	0.25	2.96E-99	monocytic cells
ENSG00000146072	7.22E-102	2.432347308	0.626	0.696	1.44E-98	monocytic cells
ENSG00000101384	3.13E-92	2.172733208	0.627	0.691	6.27E-89	monocytic cells
ENSG00000166347	3.42E-92	1.2087557	0.737	0.755	6.84E-89	monocytic cells
ENSG00000139354	5.15E-92	2.398941397	0.562	0.497	1.03E-88	monocytic cells
ENSG00000122691	1.39E-90	-9.266037789	0.275	0.211	2.78E-87	monocytic cells
ENSG00000177675	3.47E-83	1.727924507	0.319	0.681	6.93E-80	monocytic cells
ENSG00000214212	1.98E-82	1.636343232	0.631	0.775	3.95E-79	monocytic cells
ENSG00000162493	2.13E-79	1.350914117	0.573	0.577	4.27E-76	monocytic cells
ENSG00000134830	2.21E-75	1.012637022	0.567	0.614	4.42E-72	monocytic cells
ENSG00000029993	1.43E-73	2.770768394	0.666	0.758	2.86E-70	monocytic cells
ENSG00000128815	1.75E-73	1.975353307	0.556	0.489	3.50E-70	monocytic cells
ENSG00000173530	3.79E-72	2.693284471	0.566	0.535	7.58E-69	monocytic cells
ENSG00000107731	4.80E-70	1.872478879	0.556	0.603	9.60E-67	monocytic cells
ENSG00000163710	2.53E-69	2.833719489	0.531	0.46	5.06E-66	monocytic cells
ENSG00000104972	2.36E-62	2.047082105	0.599	0.592	4.72E-59	monocytic cells
ENSG00000126759	4.19E-60	1.350548514	0.645	0.746	8.38E-57	monocytic cells
ENSG00000171659	2.53E-59	2.674555988	0.544	0.599	5.06E-56	monocytic cells
ENSG00000118513	4.71E-58	1.121680951	0.645	0.685	9.41E-55	monocytic cells
ENSG00000164761	4.79E-58	1.823863386	0.471	0.699	9.59E-55	monocytic cells
ENSG00000250722	8.02E-58	1.50901664	0.414	0.688	1.60E-54	monocytic cells
ENSG00000165655	1.34E-56	1.601523169	0.592	0.679	2.68E-53	monocytic cells
ENSG00000002587	4.19E-56	1.765129724	0.571	0.655	8.37E-53	monocytic cells
ENSG00000153208	7.86E-54	1.832508611	0.486	0.412	1.57E-50	monocytic cells
ENSG00000125968	1.08E-53	1.94913805	0.649	0.689	2.15E-50	monocytic cells
ENSG00000137673	2.00E-52	2.171026736	0.655	0.759	4.01E-49	monocytic cells
ENSG00000164120	6.34E-52	-1.913909675	0.685	0.71	1.27E-48	monocytic cells
ENSG00000108950	6.67E-52	1.560138864	0.532	0.541	1.33E-48	monocytic cells
ENSG00000167772	1.15E-50	1.613687903	0.511	0.553	2.29E-47	monocytic cells

ENSG00000044459	2.67E-50	1.835361149	0.54	0.532	5.34E-47	monocytic cells
ENSG00000128422	9.62E-44	2.622881291	0.616	0.623	1.92E-40	monocytic cells
ENSG00000096696	7.51E-43	1.70031302	0.553	0.563	1.50E-39	monocytic cells
ENSG00000079215	1.28E-42	2.861538462	0.518	0.495	2.56E-39	monocytic cells
ENSG00000086548	1.41E-42	1.914013992	0.626	0.667	2.83E-39	monocytic cells
ENSG00000196664	1.52E-41	1.071341712	0.499	0.45	3.04E-38	monocytic cells
ENSG00000256612	6.33E-41	1.671621447	0.707	0.809	1.27E-37	monocytic cells
ENSG00000160326	7.04E-37	1.608317161	0.583	0.654	1.41E-33	monocytic cells
ENSG00000125845	1.45E-29	2.091347694	0.476	0.689	2.90E-26	monocytic cells
ENSG00000115380	9.36E-29	1.762429677	0.596	0.695	1.87E-25	monocytic cells
ENSG00000107562	2.51E-28	1.590360154	0.237	0.446	5.02E-25	monocytic cells
ENSG00000124507	2.67E-28	-1.143564782	0.175	0.435	5.33E-25	monocytic cells
ENSG00000159958	3.00E-24	-1.832026105	0.216	0.472	6.01E-21	monocytic cells
ENSG00000121380	5.88E-22	1.032031803	0.387	0.603	1.18E-18	monocytic cells
ENSG00000188906	1.02E-21	1.75610135	0.603	0.768	2.03E-18	monocytic cells
ENSG00000025434	1.26E-20	1.174066321	0.519	0.591	2.51E-17	monocytic cells
ENSG00000104974	1.59E-16	1.074112774	0.433	0.667	3.19E-13	monocytic cells
ENSG00000174600	2.60E-14	1.126980262	0.398	0.577	5.19E-11	monocytic cells
ENSG00000188643	7.55E-14	1.88511981	0.663	0.778	1.51E-10	monocytic cells
ENSG00000003436	4.93E-13	2.870852528	0.545	0.549	9.86E-10	monocytic cells
ENSG00000164104	4.60E-12	-35.24151756	0.739	0.692	9.20E-09	monocytic cells
ENSG00000117594	6.43E-12	2.299427882	0.495	0.63	1.29E-08	monocytic cells
ENSG00000112378	8.14E-11	-1.372954224	0.545	0.609	1.63E-07	monocytic cells
ENSG00000161243	3.10E-07	1.099277843	0.34	0.473	0.0006199	monocytic cells
ENSG00000224137	1.17E-05	1.080885799	0.4	0.572	0.023300823	monocytic cells
ENSG00000062282	0	2.220720117	0.896	0.598	0	Neutrophil
ENSG00000136869	0	1.553959315	0.878	0.692	0	Neutrophil
ENSG00000186529	0	1.092776082	0.817	0.414	0	Neutrophil
ENSG00000138772	2.74E-249	1.645906059	0.844	0.627	5.48E-246	Neutrophil
ENSG00000135636	5.13E-166	1.569534411	0.732	0.497	1.03E-162	Neutrophil
ENSG00000101307	4.52E-154	1.820633747	0.724	0.459	9.05E-151	Neutrophil
ENSG00000211592	4.24E-55	#NAME?	0.649	0.795	8.47E-52	Neutrophil
ENSG00000157551	2.21E-40	1.115786065	0.642	0.698	4.42E-37	Neutrophil
ENSG00000169435	2.04E-31	-8.368980911	0.343	0.233	4.08E-28	Neutrophil
ENSG00000154451	8.63E-06	14.81523004	0.453	0.478	0.017269448	Neutrophil
ENSG00000126878	5.69E-51	1.264665308	0.918	0.603	1.14E-47	Dendritic cells
ENSG00000155367	2.66E-50	2.366401547	0.929	0.737	5.31E-47	Dendritic cells
ENSG00000242258	1.68E-48	5.123996081	0.929	0.579	3.36E-45	Dendritic cells
ENSG00000113749	2.88E-41	1.572397573	0.888	0.617	5.77E-38	Dendritic cells
ENSG00000205726	1.99E-38	1.179345677	0.857	0.489	3.99E-35	Dendritic cells
ENSG00000075223	1.41E-36	1.908632445	0.857	0.521	2.83E-33	Dendritic cells
ENSG00000198363	1.61E-28	1.773418467	0.837	0.54	3.22E-25	Dendritic cells
ENSG00000166265	2.92E-28	2.214524954	0.776	0.226	5.84E-25	Dendritic cells
ENSG00000103316	6.97E-23	2.46797647	0.204	0.706	1.39E-19	Dendritic cells
ENSG00000198467	5.72E-22	6.078351336	0.776	0.542	1.14E-18	Dendritic cells

ENSG00000102174	1.49E-21	3.029889505	0.776	0.52	2.98E-18	Dendritic cells
ENSG00000130300	5.48E-21	3.356976303	0.755	0.5	1.10E-17	Dendritic cells
ENSG00000169083	1.23E-14	1.891618285	0.704	0.498	2.47E-11	Dendritic cells
ENSG00000204301	1.25E-12	2.078759042	0.255	0.418	2.49E-09	Dendritic cells
ENSG00000168993	2.70E-10	1.032284481	0.286	0.51	5.40E-07	Dendritic cells
ENSG00000177614	1.49E-09	1.308148608	0.408	0.54	2.97E-06	Dendritic cells
ENSG00000175600	5.43E-09	1.17524783	0.357	0.212	1.09E-05	Dendritic cells
ENSG00000171115	2.16E-08	1.29568344	0.765	0.596	4.33E-05	Dendritic cells
ENSG00000134986	2.27E-06	1.431901434	0.643	0.679	0.004542538	Dendritic cells
ENSG00000171476	7.58E-06	-1.021919486	0.541	0.674	0.015163304	Dendritic cells
