# Supplementary material for: Single-cell RNA sequencing integrated with bulk RNA sequencing analysis identifies a tumor immune microenvironment-related lncRNA signature in lung adenocarcinoma
Source: BMC Biol. 2024 Mar 22;22:69. doi: 10.1186/s12915-024-01866-5 (PMC10960411; doi:10.1186/s12915-024-01866-5)
Supplement: Supplementary file 9 — Additional file 9: Table S3. TIME-related genes with prognostic value. [file 12915_2024_1866_MOESM9_ESM.pdf]

Table S3. TIME-related genes with prognostic value.

Gene	HR	HR.95L	HR.95H	pvalue
ENSG00000102245	0.744757985	0.629680275	0.880866812	0.000579166
ENSG00000100311	1.242104904	1.067568357	1.445176398	0.005012042
ENSG00000140749	0.870207465	0.765229664	0.989586614	0.034043232
ENSG00000132334	0.850519184	0.730101243	0.990798044	0.037649566
ENSG00000168329	0.821041999	0.719422267	0.937015708	0.003444581
ENSG00000131401	0.866038832	0.771340998	0.972362755	0.014919594
ENSG00000103811	0.815770097	0.727913032	0.914231264	0.00046122
ENSG00000179639	0.900969138	0.828744866	0.979487682	0.014440811
ENSG00000108179	1.335518538	1.066716876	1.67205545	0.011628175
ENSG00000140968	0.848995277	0.742906074	0.970234334	0.016232129
ENSG00000123689	1.111486552	1.011394873	1.221483703	0.028142779
ENSG00000011422	1.224382151	1.073258478	1.396785288	0.002596881
ENSG00000223865	0.873674044	0.786081916	0.971026455	0.012230419
ENSG00000231389	0.895928063	0.81032402	0.990575468	0.031971223
ENSG00000196126	0.886598195	0.801653359	0.980543959	0.019164393
ENSG00000075624	1.629821426	1.223427359	2.171210135	0.000843852
ENSG00000204287	0.881185243	0.795230632	0.976430485	0.015716038
ENSG00000019582	0.884533256	0.795420283	0.983629785	0.023536419
ENSG00000168878	0.912754055	0.87253409	0.954827982	7.18E-05
ENSG00000196735	0.880647291	0.799705671	0.969781357	0.009773429
ENSG00000137462	0.858929421	0.765248038	0.964079243	0.009857005
ENSG00000101160	0.812403701	0.670669507	0.984090923	0.033677656
ENSG00000136826	1.139055231	1.013911674	1.279644818	0.028334046
ENSG00000158481	0.855283993	0.773212692	0.946066607	0.002388342
ENSG00000176597	1.333268078	1.134985151	1.56619121	0.000463061
ENSG00000104870	0.753279642	0.625568173	0.907063759	0.002798456
ENSG00000116962	1.186250756	1.034150416	1.360721646	0.014703055
ENSG00000204388	1.25268318	1.060683512	1.479437676	0.007954575
ENSG00000277443	1.207942438	1.012309973	1.441381565	0.036110079
ENSG00000111275	0.819413908	0.706042339	0.950989927	0.008757882
ENSG00000232629	0.912267824	0.843649707	0.986466986	0.021364469
ENSG00000242574	0.856324104	0.763211418	0.960796646	0.00826889
ENSG00000173110	1.139851252	1.021705962	1.27165831	0.019047811
ENSG00000163191	1.381102446	1.117245286	1.707274123	0.002837115
ENSG00000204389	1.228853874	1.056405277	1.429453142	0.007557922
ENSG00000166428	0.796667243	0.671155398	0.945650884	0.009353973
ENSG00000165795	0.823862545	0.712198305	0.953034413	0.009125959
ENSG00000010671	0.813988517	0.705516802	0.939137528	0.004794619
ENSG00000227507	0.888327645	0.795315128	0.992218023	0.03586925
ENSG00000153395	0.89040018	0.797074008	0.994653537	0.039892728
ENSG00000119655	0.829974607	0.736203718	0.935689172	0.002313958
ENSG00000163661	1.176110421	1.033170599	1.33882606	0.01414566
ENSG00000135549	1.185132211	1.072277065	1.309865149	0.00087862

ENSG00000104312	1.465593661	1.219057674	1.761987825	4.75E-05
ENSG00000172216	1.35188532	1.10820634	1.649145878	0.002947893
ENSG00000121316	1.141242071	1.008750145	1.291135839	0.035875791
ENSG00000038427	1.123425921	1.014127143	1.244504507	0.025841309
ENSG00000120708	1.110803148	1.005417525	1.227235057	0.038809744
ENSG00000172243	0.872903321	0.773999311	0.984445589	0.026727768
ENSG00000185022	1.253428132	1.02704347	1.529713327	0.026248341
ENSG00000051108	0.70946718	0.566034862	0.889245015	0.002895779
ENSG00000130513	0.895450666	0.819409027	0.978549013	0.014732929
ENSG00000197253	0.908440442	0.834231873	0.989250187	0.027206511
ENSG00000137331	1.185060126	1.049255672	1.338441659	0.006252753
ENSG00000132514	0.847556544	0.751751972	0.955570615	0.006881033
ENSG00000196923	1.362393704	1.14088643	1.626907426	0.000635766
ENSG00000100368	0.872963746	0.764507376	0.996806212	0.044725735
ENSG00000059728	1.250880149	1.048157352	1.492811307	0.013089367
ENSG00000129521	1.154662649	1.057771952	1.260428422	0.001300034
ENSG00000115008	1.197171686	1.067874296	1.342124304	0.002027841
ENSG00000204257	0.822561228	0.727713627	0.929770926	0.001778839
ENSG00000162896	0.94335674	0.896849354	0.992275832	0.023785603
ENSG00000113269	0.757895014	0.591312636	0.971406355	0.028593147
ENSG00000204252	0.884297668	0.798995424	0.978706938	0.017509747
ENSG00000072110	1.324191925	1.11230893	1.576436372	0.001597337
ENSG00000163535	1.407404414	1.192083593	1.661617689	5.49E-05
ENSG00000182718	1.345535	1.118004142	1.619371851	0.001688649
ENSG00000124882	1.082926266	1.019695319	1.150078141	0.00944939
ENSG00000131400	0.906590236	0.861796362	0.953712377	0.000148767
ENSG00000148926	1.256862552	1.132963526	1.394310972	1.58E-05
ENSG00000198435	1.260956204	1.081760557	1.469835943	0.003028055
ENSG00000158488	0.847169871	0.757943452	0.946900179	0.003490838
ENSG00000165140	0.808347388	0.709843574	0.920520413	0.001331699
ENSG00000117724	1.243436843	1.109105751	1.394037657	0.000187513
ENSG00000160213	1.230773399	1.026555431	1.475617502	0.024891018
ENSG00000211445	0.86779307	0.765909897	0.983228988	0.026054739
ENSG00000111341	0.859925792	0.769838117	0.960555669	0.007524358
ENSG00000112096	1.193742599	1.009986987	1.410930449	0.037849197
ENSG00000159399	1.189398603	1.052711109	1.343834053	0.005357974
ENSG00000102265	1.188807556	1.002268538	1.410064619	0.047035102
ENSG00000114270	1.155274785	1.051332312	1.269493779	0.002694373
ENSG00000181631	0.813345719	0.709959177	0.931787743	0.002896409
ENSG00000139370	1.363014005	1.009299652	1.840689409	0.043347002
ENSG00000122641	1.137953555	1.017304565	1.27291112	0.023821426
ENSG00000179344	0.896096032	0.818455995	0.981101127	0.017663781
ENSG00000160593	0.822683663	0.712209612	0.950293844	0.007979392
ENSG00000138623	1.186720545	1.040450527	1.353553692	0.010747227
ENSG00000075618	1.272170719	1.142072776	1.417088623	1.22E-05

ENSG00000114450	1.202017952	1.013723312	1.42528749	0.034279875
ENSG00000123975	1.209775595	1.049006565	1.395183825	0.00885532
ENSG00000134755	1.240258499	1.086017252	1.416405809	0.001484024
ENSG00000164265	0.9487373	0.908227714	0.991053731	0.018098473
ENSG00000139970	0.835880428	0.708990008	0.985480871	0.032837031
ENSG00000100558	1.39440786	1.216899825	1.597808825	1.70E-06
ENSG00000135047	1.307337253	1.145468951	1.492079459	7.07E-05
ENSG00000138166	1.185304614	1.0364488	1.355539251	0.013034647
ENSG00000158050	0.863057839	0.745405361	0.99928022	0.048884968
ENSG00000211751	0.751204657	0.608709911	0.92705643	0.007682017
ENSG00000124762	1.196135813	1.00462108	1.424159726	0.044246076
ENSG00000189377	0.867176244	0.813313713	0.924605876	1.33E-05
ENSG00000092964	0.803235443	0.697683566	0.924756162	0.002301871
ENSG00000060138	1.34298961	1.064747306	1.693942856	0.012788293
ENSG00000186074	0.857346036	0.75320098	0.975891224	0.019843699
ENSG00000108924	0.847202564	0.764272059	0.939131788	0.001606218
ENSG00000166927	0.87896875	0.775319893	0.996473934	0.043889948
ENSG00000116285	1.218205021	1.076940334	1.377999715	0.001697185
ENSG00000112137	0.810685083	0.685370608	0.958912296	0.014298677
ENSG00000103257	1.192182422	1.063498988	1.336436559	0.002558204
ENSG00000068366	1.191950483	1.018562083	1.394854548	0.028576862
ENSG00000129538	0.866259	0.788186024	0.952065416	0.002889043
ENSG00000136810	1.332205104	1.12771389	1.573777229	0.000741916
ENSG00000181649	1.142752185	1.010939215	1.291751806	0.03284663
ENSG00000085265	0.879202726	0.780460254	0.990437924	0.034171936
ENSG00000143416	0.854315227	0.77344469	0.9436415	0.00191407
ENSG00000157404	0.892430014	0.820546827	0.970610456	0.00790324
ENSG00000229644	1.138154684	1.02419616	1.264792954	0.016211276
ENSG00000115919	1.24733473	1.132848611	1.373390861	6.82E-06
ENSG00000116191	1.261500803	1.048244512	1.518142245	0.013946454
ENSG00000115590	1.226309918	1.103296999	1.363038254	0.000155166
ENSG00000169908	1.156741792	1.014958061	1.318331883	0.029071885
ENSG00000069849	1.281113184	1.038141391	1.58095131	0.020953085
ENSG00000178381	1.253762827	1.042550631	1.50776488	0.016275341
ENSG00000196230	1.263578272	1.04525076	1.527509101	0.015638537
ENSG00000149534	0.796584734	0.687172434	0.923417773	0.002553924
ENSG00000109805	1.270326282	1.12989462	1.42821183	6.25E-05
ENSG00000157227	1.230518896	1.078508102	1.403954918	0.002046585
ENSG00000124216	1.201525396	1.049237992	1.37591594	0.007929557
ENSG00000148773	1.259995439	1.124287234	1.412084436	7.04E-05
ENSG00000172236	0.907310921	0.826295904	0.996269138	0.041520917
ENSG00000071205	1.349101562	1.081418732	1.683043736	0.007963195
ENSG00000172215	0.82628879	0.703434765	0.970599121	0.020162093
ENSG00000159388	0.819407266	0.725428382	0.925561068	0.001352766
ENSG00000163599	0.861033059	0.744251581	0.996138868	0.044221469

ENSG00000141543	1.40706978	1.120112469	1.767541581	0.003338755
ENSG00000138678	1.202337487	1.051761835	1.374470327	0.006950251
ENSG00000151012	1.117693176	1.026451423	1.217045452	0.010441992
ENSG00000180879	0.806532953	0.652286788	0.997253688	0.047104691
ENSG00000153064	0.834941532	0.712787288	0.978030014	0.025402571
ENSG00000145414	1.443348881	1.054904512	1.974828973	0.021784085
ENSG00000176014	1.257999891	1.085879492	1.45740272	0.002232
ENSG00000211679	0.912410766	0.846130171	0.983883371	0.017208606
ENSG00000126353	0.861816055	0.760364743	0.97680346	0.019952064
ENSG00000138135	0.889666225	0.810705616	0.976317392	0.013686193
ENSG00000141682	1.140944384	1.021783282	1.274002141	0.019136561
ENSG00000164692	1.135808571	1.025764929	1.25765765	0.014316388
ENSG00000129993	0.732984646	0.610779059	0.879641311	0.000843694
ENSG00000198502	0.89025623	0.819404114	0.967234777	0.006009038
ENSG00000211598	0.929616531	0.869029267	0.994427838	0.033798756
ENSG00000117322	0.865302066	0.780272181	0.959598052	0.006117448
ENSG00000173114	0.761791561	0.613561459	0.945832522	0.013725393
ENSG00000129244	0.789126572	0.641677627	0.970457314	0.02482465
ENSG00000116717	1.177335549	1.026137598	1.350812014	0.019918518
ENSG00000176890	1.337412593	1.165841689	1.534232701	3.32E-05
ENSG00000104738	1.293143287	1.12745029	1.483186953	0.000238164
ENSG00000204305	0.906802518	0.845221763	0.972869894	0.00640058
ENSG00000135929	0.835494543	0.734164474	0.950810282	0.006437692
ENSG00000168675	0.804006827	0.660628922	0.978502388	0.029493938
ENSG00000163751	0.897348487	0.827000912	0.973680071	0.009313873
ENSG00000135480	1.257007759	1.087870702	1.452441454	0.001920608
ENSG00000230006	0.807658217	0.687716688	0.948518202	0.009204915
ENSG00000019991	0.802701055	0.683461466	0.94274369	0.007393647
ENSG00000141655	1.269416953	1.056331426	1.525486567	0.010943758
ENSG00000168243	1.088062034	1.002672238	1.180723815	0.042974088
ENSG00000099958	0.882937222	0.779677716	0.999872283	0.049765189
ENSG00000093072	0.842715063	0.740129979	0.959518865	0.009768277
ENSG00000166562	0.872263258	0.763045405	0.997113916	0.045251389
ENSG00000170113	1.398273678	1.094384518	1.786546908	0.007332546
ENSG00000099194	1.135770633	1.004126941	1.28467316	0.042817351
ENSG00000072274	1.137727781	1.002676843	1.290968784	0.045346184
ENSG00000170265	1.334643113	1.021774783	1.743311999	0.034173562
ENSG00000149212	0.874171425	0.765195694	0.99866699	0.047748527
ENSG00000160856	0.782648597	0.651233434	0.940582585	0.008972135
ENSG00000240403	0.611838179	0.404768024	0.924840737	0.019773228
ENSG00000211899	0.918623296	0.859179908	0.982179345	0.01289052
ENSG00000270164	0.75840196	0.615538246	0.934423712	0.009407442
ENSG00000105369	0.875830509	0.801735413	0.956773354	0.003284628
ENSG00000132906	1.383724209	1.0916989	1.75386518	0.007244507
ENSG00000164825	1.072144601	1.008692391	1.139588298	0.025219711

ENSG00000023445	1.239920669	1.101839746	1.395305687	0.000357107
ENSG00000048462	0.869775191	0.780590071	0.96915002	0.011482197
ENSG00000172322	0.849561109	0.73549571	0.981316503	0.026667118
ENSG00000088826	1.304641894	1.140186175	1.492818021	0.000109573
ENSG00000163687	0.794641884	0.678616903	0.930503972	0.004311735
ENSG00000181374	0.919730228	0.846796315	0.998945883	0.047146303
ENSG00000108821	1.123421542	1.02632831	1.229700037	0.011620703
ENSG00000125148	1.176652388	1.051473826	1.316733531	0.004588754
ENSG00000131747	1.180005083	1.06437836	1.308192695	0.00165681
ENSG00000175445	0.91558767	0.842365065	0.995175152	0.038108053
ENSG00000066279	1.324211412	1.165807092	1.504138958	1.56E-05
ENSG00000135451	1.178726329	1.057923801	1.313323094	0.002876426
ENSG00000140287	0.79164854	0.663972161	0.943876036	0.009223901
ENSG00000128965	1.177096841	1.006373691	1.37678179	0.04140686
ENSG00000111796	0.80530916	0.695646925	0.932258622	0.003741768
ENSG00000137807	1.284690991	1.134433025	1.454850932	7.90E-05
ENSG00000118193	1.360293597	1.1910146	1.553632231	5.68E-06
ENSG00000143297	0.862239321	0.747970626	0.993965031	0.041011376
ENSG00000211753	0.867086637	0.761586158	0.987201813	0.03119637
ENSG00000179163	0.77105952	0.6444232	0.922581284	0.004507705
ENSG00000129116	1.187410708	1.024668854	1.375999852	0.022372085
ENSG00000171848	1.308841639	1.166944063	1.467993618	4.29E-06
ENSG00000163993	1.066188749	1.014605079	1.120394991	0.011308431
ENSG00000269404	0.848278541	0.741356166	0.970621836	0.016677175
ENSG00000122254	0.832143115	0.739568235	0.936305984	0.002260502
ENSG00000142156	1.165867629	1.029150593	1.320746775	0.015888336
ENSG00000088325	1.252841163	1.131773108	1.386860113	1.38E-05
ENSG00000158825	1.091438474	1.016857525	1.17148953	0.015398147
ENSG00000162998	0.839015086	0.738403789	0.953335188	0.007076622
ENSG00000115963	1.21096987	1.0702675	1.370169632	0.002384932
ENSG00000170442	1.219930459	1.079564606	1.378546792	0.001435027
ENSG00000123411	0.718814784	0.573991271	0.90017866	0.004026632
ENSG00000007312	0.84020239	0.744588864	0.948093762	0.004732431
ENSG00000006451	1.404096118	1.079363893	1.826525718	0.01143667
ENSG00000156738	0.841371573	0.762935272	0.927871798	0.000541562
ENSG00000115009	1.105607647	1.031592612	1.184933136	0.004514704
ENSG00000144959	1.215548268	1.034877048	1.427761485	0.017428708
ENSG00000097021	1.352411887	1.109964542	1.647816523	0.002744631
ENSG00000196092	0.806900644	0.685027152	0.950456706	0.010223325
ENSG00000176046	0.849279203	0.746065741	0.966771592	0.013468939
ENSG00000211593	0.916595574	0.853731233	0.984088918	0.016287346
ENSG00000159166	1.213358901	1.036712294	1.420104527	0.015992123
ENSG00000110848	0.860446267	0.759549492	0.974745934	0.018181136
ENSG00000188505	1.109209967	1.026950762	1.198058169	0.008378726
ENSG00000132465	0.879577171	0.811535438	0.953323741	0.001786563

ENSG00000137804	1.272797239	1.118864107	1.447908464	0.00024475
ENSG00000149257	1.366696738	1.124818812	1.660587425	0.001669055
ENSG00000132170	1.127594082	1.00177992	1.269209322	0.046654306
ENSG00000142173	1.148439189	1.017054264	1.296796659	0.025564219
ENSG00000135919	1.11494605	1.007671712	1.23364056	0.035028389
ENSG00000161638	1.258907392	1.101384067	1.438960186	0.000735914
ENSG00000203668	1.200627685	1.055696491	1.365455744	0.005340499
ENSG00000127824	1.298994882	1.093382391	1.543273166	0.002925896
ENSG00000102445	0.752418788	0.613152273	0.923317188	0.006450793
ENSG00000091409	1.264351548	1.132664687	1.411348702	2.92E-05
ENSG00000168542	1.104210229	1.004329458	1.214024164	0.040435576
ENSG00000122224	0.691962405	0.548001012	0.873742858	0.001974327
ENSG00000125498	0.561421838	0.348661486	0.904012898	0.01754176
ENSG00000160789	1.265024231	1.024868825	1.56145476	0.028621844
ENSG00000012124	0.824476936	0.71798229	0.946767389	0.006234896
ENSG00000123610	1.170586805	1.035280793	1.323576635	0.011963633
ENSG00000157765	0.892771574	0.836315854	0.953038351	0.000666164
ENSG00000177706	1.221255159	1.034157951	1.442201516	0.018481036
ENSG00000137265	0.841222929	0.738698866	0.957976313	0.009123124
ENSG00000107317	0.866508496	0.786019221	0.955239966	0.003969369
ENSG00000131459	1.265450338	1.098640019	1.457588045	0.0010972
ENSG00000143185	0.850271083	0.726120025	0.995649327	0.043998409
ENSG00000174944	0.796136741	0.660785698	0.959212212	0.016486209
ENSG00000165272	0.910413757	0.843228163	0.982952474	0.016414506
ENSG00000102096	0.85613585	0.740128727	0.990325828	0.036544166
ENSG00000087586	1.279101904	1.12637675	1.452535026	0.000148024
ENSG00000131016	1.200706847	1.093271063	1.318700348	0.000131017
ENSG00000138778	1.338748202	1.157396729	1.548515477	8.56E-05
ENSG00000197696	1.206146772	1.064087791	1.367171062	0.003373046
ENSG00000035720	0.731537471	0.619158295	0.864313821	0.000239226
ENSG00000133063	0.905646592	0.839082188	0.977491552	0.010945054
ENSG00000104419	1.165807404	1.020087934	1.332342888	0.024328298
ENSG00000106366	1.158696804	1.0472605	1.281990759	0.004303389
ENSG00000187601	0.801065665	0.668572361	0.959815627	0.016190993
ENSG00000163132	1.289133838	1.063427923	1.56274442	0.009704128
ENSG00000123989	1.253413217	1.046914565	1.500642694	0.013927911
ENSG00000124466	1.211109072	1.120936991	1.308534909	1.22E-06
ENSG00000138180	1.265538981	1.124126332	1.424741034	9.81E-05
ENSG00000163808	1.215040816	1.056061569	1.39795276	0.006482105
ENSG00000112742	1.2225834	1.083621191	1.379365947	0.001096602
ENSG00000143228	1.198032231	1.068656402	1.343070816	0.001942966
ENSG00000126787	1.318174651	1.175109923	1.478656912	2.44E-06
ENSG00000136026	1.460675249	1.19881696	1.779731396	0.000170641
ENSG00000123485	1.302236167	1.161386985	1.460167073	6.13E-06
ENSG00000170312	1.280055391	1.134651789	1.444092206	5.99E-05

ENSG00000136573	0.790045189	0.670206207	0.931312474	0.004988227
ENSG00000138160	1.298527427	1.140731112	1.478151565	7.76E-05
ENSG00000068489	1.279612186	1.131499026	1.447113351	8.55E-05
ENSG00000239951	0.928266185	0.862431724	0.999126175	0.04733885
ENSG00000132185	0.823615404	0.723740741	0.937272556	0.003259329
ENSG00000072571	1.361800887	1.197668306	1.548426762	2.45E-06
ENSG00000189057	1.262079781	1.108838982	1.436498354	0.00042471
ENSG00000127074	0.552443764	0.381403463	0.800187051	0.00169417
ENSG00000110777	0.872030324	0.779887908	0.975059208	0.016250292
ENSG00000175063	1.177997364	1.073011383	1.293255423	0.00058263
ENSG00000234184	0.788048131	0.66927417	0.92790053	0.004266695
ENSG00000122952	1.26350367	1.108192509	1.440581407	0.000473857
ENSG00000170509	0.846116938	0.740245453	0.967130389	0.014285653
ENSG00000135114	1.151625556	1.021637151	1.298153087	0.020873083
ENSG00000164938	0.772044675	0.640296939	0.930900873	0.006728455
ENSG00000075218	1.299537307	1.142549307	1.478095696	6.64E-05
ENSG00000226777	0.765117118	0.637351736	0.91849472	0.004078912
ENSG00000089685	1.232350683	1.105130013	1.374216778	0.00017122
ENSG00000161055	0.933839859	0.89300277	0.976544431	0.002696963
ENSG00000163106	0.82917119	0.732892906	0.938097309	0.002932833
ENSG00000106034	0.821589952	0.697423926	0.967861904	0.018737334
ENSG00000237649	1.216306263	1.085064921	1.363421577	0.000775562
ENSG00000169116	0.877674066	0.796005299	0.967721906	0.008834756
ENSG00000123838	0.902929976	0.85119122	0.957813617	0.000694847
ENSG00000091436	1.287923881	1.061224253	1.563051277	0.010421358
ENSG00000101443	0.866470167	0.794418169	0.945057124	0.001213404
ENSG00000196260	0.927207384	0.868530461	0.989848453	0.023459473
ENSG00000135052	1.286796035	1.104239114	1.499533946	0.00123708
ENSG00000139618	1.39668727	1.112577901	1.753347185	0.003985039
ENSG00000101384	1.287734256	1.117049714	1.484499297	0.000490838
ENSG00000139354	1.269212704	1.054282139	1.527959952	0.011790105
ENSG00000122691	1.139003843	1.020850212	1.270832624	0.0198449
ENSG00000128815	0.787709581	0.679541616	0.913095489	0.001543891
ENSG00000250722	0.850080422	0.759550962	0.951399918	0.004696599
ENSG00000153208	0.837885758	0.708306881	0.991170016	0.039071468
ENSG00000125968	1.131348007	1.021201727	1.253374606	0.018205484
ENSG00000167772	1.179708361	1.086845353	1.280505836	7.79E-05
ENSG00000044459	1.34430263	1.073912515	1.682771674	0.009813648
ENSG00000128422	1.094054479	1.026110596	1.166497263	0.005997651
ENSG00000096696	1.122388379	1.019524216	1.235630947	0.018561111
ENSG00000196664	0.819981982	0.69965352	0.961004885	0.014237742
ENSG00000256612	0.902634705	0.846678959	0.962288483	0.001705319
ENSG00000159958	0.85684515	0.747102109	0.982708524	0.027146546
ENSG00000188643	1.263919347	1.106482369	1.443757407	0.000559043
ENSG00000164104	1.237456098	1.052345971	1.455127531	0.009962776

ENSG00000112378	1.346389088	1.156356166	1.567651586	0.000127457
ENSG00000224137	0.820113573	0.71625556	0.939031138	0.004097956
ENSG00000186529	1.093420014	1.010178859	1.183520439	0.02706072
ENSG00000157551	0.906241359	0.826291142	0.993927394	0.036687452
ENSG00000116748	0.720546231	0.582794086	0.890858167	0.00246555
ENSG00000171241	1.371388305	1.186209731	1.585475008	1.98E-05
ENSG00000164440	0.784874018	0.616044334	0.999972227	0.049973735
ENSG00000077943	0.851021414	0.74503095	0.972090417	0.017450873
ENSG00000108405	0.720838114	0.592569711	0.876871661	0.001059436
ENSG00000103089	1.124654735	1.015763731	1.245218976	0.023759709
ENSG00000125398	1.091593869	1.002151655	1.189018817	0.044511624
ENSG00000242258	0.70439109	0.559116522	0.887412172	0.002943954
ENSG00000075223	1.219194635	1.081696762	1.374170294	0.00116932
ENSG00000198363	1.216450998	1.080598485	1.369382848	0.001183263
ENSG00000198467	1.189275715	1.02819078	1.375597558	0.019577801
ENSG00000171115	0.848938566	0.724264122	0.995074404	0.04329055
ENSG00000171476	0.91414559	0.84602672	0.987749133	0.023089768
