# Supplementary material for: Single-cell RNA sequencing integrated with bulk RNA sequencing analysis identifies a tumor immune microenvironment-related lncRNA signature in lung adenocarcinoma
Source: BMC Biol. 2024 Mar 22;22:69. doi: 10.1186/s12915-024-01866-5 (PMC10960411; doi:10.1186/s12915-024-01866-5)
Supplement: Supplementary file 10 — Additional file 10: Table S4. LncRNAs from associated modules between distinct immune infiltration clusters. [file 12915_2024_1866_MOESM10_ESM.pdf]

Table S4. LncRNAs from associated modules between distinct immune infiltration clusters.

lncRNA	module
ENSG00000260244	module 4
ENSG00000268388	module 4
ENSG00000255399	module 4
ENSG00000267280	module 4
ENSG00000267107	module 4
ENSG00000250431	module 4
ENSG00000186594	module 4
ENSG00000261269	module 4
ENSG00000267745	module 4
ENSG00000261888	module 4
ENSG00000261754	module 4
ENSG00000261863	module 4
ENSG00000246430	module 4
ENSG00000244215	module 4
ENSG00000224239	module 4
ENSG00000237248	module 4
ENSG00000236304	module 4
ENSG00000272274	module 4
ENSG00000233968	module 4
ENSG00000249201	module 4
ENSG00000259847	module 4
ENSG00000254453	module 4
ENSG00000224958	module 4
ENSG00000255389	module 4
ENSG00000269186	module 4
ENSG00000198358	module 4
ENSG00000260695	module 4
ENSG00000275830	module 4
ENSG00000233760	module 4
ENSG00000225670	module 4
ENSG00000241158	module 4
ENSG00000267272	module 4
ENSG00000267532	module 4
ENSG00000271474	module 4
ENSG00000226031	module 4
ENSG00000227544	module 4
ENSG00000248441	module 4
ENSG00000241720	module 4
ENSG00000254192	module 4
ENSG00000234880	module 4
ENSG00000224957	module 4

ENSG00000254102	module 4
ENSG00000226252	module 4
ENSG00000264705	module 4
ENSG00000175967	module 4
ENSG00000267097	module 4
ENSG00000232046	module 4
ENSG00000261340	module 4
ENSG00000227128	module 4
ENSG00000237179	module 4
ENSG00000255136	module 4
ENSG00000268505	module 4
ENSG00000261222	module 4
ENSG00000234944	module 4
ENSG00000256508	module 4
ENSG00000268926	module 4
ENSG00000233429	module 5
ENSG00000224397	module 5
ENSG00000256262	module 5
ENSG00000259225	module 5
ENSG00000251442	module 5
ENSG00000226777	module 5
ENSG00000234883	module 5
ENSG00000271856	module 5
ENSG00000204261	module 5
ENSG00000229228	module 5
ENSG00000237181	module 5
ENSG00000256128	module 5
ENSG00000273445	module 5
ENSG00000146666	module 5
ENSG00000242258	module 5
ENSG00000280721	module 5
ENSG00000224875	module 5
ENSG00000254703	module 5
ENSG00000233093	module 5
ENSG00000266709	module 5
ENSG00000228857	module 5
ENSG00000235621	module 5
ENSG00000271046	module 5
ENSG00000132832	module 5
ENSG00000260101	module 5
ENSG00000258867	module 5
ENSG00000240350	module 5
ENSG00000234636	module 5
ENSG00000188511	module 5

ENSG00000235304	module 5
ENSG00000259436	module 5
ENSG00000264707	module 5
ENSG00000234699	module 5
ENSG00000267496	module 5
ENSG00000251002	module 5
ENSG00000256862	module 5
ENSG00000281162	module 5
ENSG00000249993	module 5
ENSG00000272917	module 5
ENSG00000242048	module 5
ENSG00000246084	module 5
ENSG00000205056	module 5
ENSG00000249988	module 5
ENSG00000245904	module 5
ENSG00000185433	module 5
ENSG00000226806	module 5
ENSG00000227145	module 5
ENSG00000179840	module 5
ENSG00000253490	module 5
ENSG00000216863	module 5
ENSG00000276241	module 5
ENSG00000122043	module 5
ENSG00000241490	module 5
ENSG00000272405	module 12
ENSG00000223784	module 12
ENSG00000261116	module 12
ENSG00000167912	module 12
ENSG00000255100	module 12
ENSG00000254528	module 12
ENSG00000231131	module 12
ENSG00000261578	module 12
ENSG00000177337	module 12
ENSG00000228705	module 12
ENSG00000237523	module 12
ENSG00000223813	module 12
ENSG00000269887	module 12
ENSG00000254290	module 12
ENSG00000224167	module 12
ENSG00000180525	module 12
ENSG00000224417	module 12
ENSG00000251191	module 12
ENSG00000226733	module 12
ENSG00000236882	module 12

ENSG00000251161	module 12
ENSG00000260896	module 12
ENSG00000258285	module 12
ENSG00000249937	module 12
ENSG00000260878	module 12
ENSG00000178107	module 12
ENSG00000237685	module 12
ENSG00000250033	module 12
ENSG00000236671	module 12
ENSG00000232721	module 12
ENSG00000189295	module 12
ENSG00000280837	module 12
ENSG00000278090	module 12
ENSG00000245534	module 12
ENSG00000237166	module 12
ENSG00000185186	module 12
ENSG00000280179	module 12
ENSG00000188660	module 12
ENSG00000254560	module 13
ENSG00000237978	module 13
ENSG00000233854	module 13
ENSG00000236081	module 13
ENSG00000269210	module 13
ENSG00000259129	module 13
ENSG00000229921	module 13
ENSG00000250682	module 13
ENSG00000269994	module 13
ENSG00000262117	module 13
ENSG00000267123	module 13
ENSG00000198054	module 13
ENSG00000266602	module 13
ENSG00000229404	module 13
ENSG00000269416	module 13
ENSG00000266830	module 13
ENSG00000228630	module 13
ENSG00000265246	module 13
ENSG00000276476	module 13
ENSG00000229970	module 13
ENSG00000228496	module 13
ENSG00000251138	module 13
ENSG00000234722	module 13
ENSG00000251629	module 13
ENSG00000256546	module 13
ENSG00000259985	module 13

ENSG00000224141	module 13
ENSG00000249001	module 13
ENSG00000273108	module 13
ENSG00000270816	module 13
ENSG00000253877	module 13
ENSG00000226686	module 13
ENSG00000259692	module 13
ENSG00000267260	module 13
ENSG00000253706	module 13
ENSG00000259345	module 13
ENSG00000226562	module 13
ENSG00000263745	module 13
ENSG00000224272	module 13
ENSG00000250519	module 13
ENSG00000248429	module 13
ENSG00000261761	module 13
ENSG00000251151	module 13
ENSG00000267309	module 13
ENSG00000279516	module 13
ENSG00000257636	module 13
ENSG00000250590	module 13
ENSG00000250546	module 13
ENSG00000204044	module 13
ENSG00000204904	module 13
ENSG00000184029	module 13
ENSG00000225535	module 13
ENSG00000227279	module 13
ENSG00000240758	module 13
ENSG00000254349	module 13
ENSG00000253661	module 13
ENSG00000245662	module 13
ENSG00000231609	module 13
ENSG00000257194	module 13
ENSG00000230426	module 13
ENSG00000261319	module 13
ENSG00000248285	module 13
ENSG00000183146	module 13
ENSG00000225778	module 13
ENSG00000262768	module 13
ENSG00000249894	module 13
ENSG00000226747	module 13
ENSG00000259868	module 13
ENSG00000260971	module 13
ENSG00000239589	module 13

ENSG00000278932	module 13
ENSG00000269072	module 13
ENSG00000254814	module 13
ENSG00000253859	module 13
ENSG00000276272	module 13
ENSG00000234352	module 13
ENSG00000236268	module 13
ENSG00000248118	module 13
ENSG00000250708	module 13
ENSG00000233316	module 13
ENSG00000184856	module 13
ENSG00000272046	module 13
ENSG00000228956	module 13
ENSG00000259033	module 13
ENSG00000231421	module 13
ENSG00000261060	module 13
ENSG00000258785	module 13
ENSG00000257056	module 13
ENSG00000250392	module 13
ENSG00000231817	module 13
ENSG00000271860	module 13
ENSG00000220891	module 13
ENSG00000241369	module 13
ENSG00000235725	module 13
ENSG00000273840	module 13
ENSG00000250448	module 13
ENSG00000243083	module 13
ENSG00000256115	module 13
ENSG00000230010	module 13
ENSG00000261194	module 13
ENSG00000272620	module 21
ENSG00000225383	module 21
ENSG00000235584	module 21
ENSG00000224189	module 21
ENSG00000248801	module 21
ENSG00000225329	module 21
ENSG00000225342	module 21
ENSG00000259974	module 21
ENSG00000256969	module 21
ENSG00000235026	module 21
ENSG00000230257	module 21
ENSG00000181577	module 21
ENSG00000248890	module 21
ENSG00000260265	module 21

ENSG00000260997	module 21
ENSG00000236318	module 21
ENSG00000215808	module 21
ENSG00000259353	module 21
ENSG00000250742	module 21
ENSG00000260740	module 21
ENSG00000228709	module 21
ENSG00000230798	module 21
ENSG00000247134	module 21
ENSG00000278910	module 21
ENSG00000250786	module 21
ENSG00000276980	module 21
ENSG00000237686	module 21
ENSG00000227640	module 21
ENSG00000243479	module 21
ENSG00000218357	module 21
ENSG00000205611	module 21
ENSG00000204792	module 21
ENSG00000189223	module 21
ENSG00000244300	module 21
ENSG00000261373	module 21
ENSG00000242375	module 21
ENSG00000226363	module 21
ENSG00000227195	module 21
ENSG00000261600	module 21
ENSG00000203706	module 21
ENSG00000254109	module 21
ENSG00000225742	module 21
ENSG00000233834	module 21
ENSG00000226051	module 21
ENSG00000271133	module 21
ENSG00000233338	module 21
ENSG00000233101	module 21
ENSG00000257084	module 21
ENSG00000259439	module 21
ENSG00000255571	module 21
ENSG00000259153	module 21
ENSG00000238142	module 21
ENSG00000234380	module 21
ENSG00000242516	module 21
ENSG00000250328	module 21
ENSG00000265055	module 21
ENSG00000278709	module 21
ENSG00000233559	module 21

ENSG00000248874	module 21
ENSG00000233392	module 21
ENSG00000229196	module 21
ENSG00000256751	module 21
ENSG00000257894	module 21
ENSG00000239911	module 21
ENSG00000265750	module 21
ENSG00000247516	module 21
ENSG00000266010	module 21
ENSG00000230082	module 21
ENSG00000224063	module 21
ENSG00000249307	module 21
ENSG00000272138	module 21
ENSG00000177335	module 21
ENSG00000229891	module 21
ENSG00000230733	module 21
ENSG00000260418	module 21
ENSG00000247095	module 21
ENSG00000250584	module 21
ENSG00000272767	module 21
ENSG00000260025	module 21
ENSG00000175061	module 21
ENSG00000230368	module 21
ENSG00000266680	module 21
ENSG00000261584	module 21
ENSG00000272933	module 21
ENSG00000261742	module 21
ENSG00000235978	module 21
ENSG00000167920	module 21
ENSG00000225978	module 21
ENSG00000248663	module 21
ENSG00000229896	module 21
ENSG00000197595	module 21
ENSG00000225434	module 21
ENSG00000235501	module 21
ENSG00000197568	module 21
ENSG00000253552	module 21
ENSG00000269397	module 21
ENSG00000235947	module 21
ENSG00000233621	module 21
ENSG00000260317	module 21
ENSG00000248636	module 21
ENSG00000237512	module 21
ENSG00000232956	module 21

ENSG00000259065	module 21
ENSG00000261061	module 21
ENSG00000261305	module 21
ENSG00000278730	module 21
ENSG00000260912	module 21
ENSG00000273313	module 21
ENSG00000228044	module 21
ENSG00000247903	module 21
ENSG00000237380	module 21
ENSG00000227591	module 21
ENSG00000280206	module 21
ENSG00000245910	module 21
ENSG00000236751	module 21
ENSG00000273001	module 21
ENSG00000255145	module 21
ENSG00000272330	module 21
ENSG00000237187	module 21
ENSG00000260810	module 21
ENSG00000203721	module 21
ENSG00000255366	module 21
ENSG00000256576	module 21
ENSG00000259969	module 21
ENSG00000253716	module 21
ENSG00000272913	module 21
ENSG00000233930	module 21
ENSG00000255650	module 21
ENSG00000264785	module 21
ENSG00000239335	module 21
ENSG00000204588	module 21
ENSG00000237424	module 21
ENSG00000247317	module 21
ENSG00000269888	module 21
ENSG00000226067	module 21
ENSG00000229116	module 21
ENSG00000276855	module 21
ENSG00000271868	module 21
ENSG00000227089	module 21
ENSG00000225518	module 21
ENSG00000260804	module 21
ENSG00000228343	module 21
ENSG00000269893	module 21
ENSG00000230091	module 21
ENSG00000180066	module 21
ENSG00000257027	module 21

ENSG00000233593	module 21
ENSG00000260193	module 21
ENSG00000172460	module 21
ENSG00000246223	module 21
ENSG00000218537	module 21
ENSG00000266208	module 21
ENSG00000235663	module 21
ENSG00000233251	module 21
ENSG00000223561	module 21
ENSG00000224090	module 21
ENSG00000204387	module 21
ENSG00000270362	module 21
ENSG00000246640	module 21
ENSG00000231566	module 21
ENSG00000267655	module 21
ENSG00000255652	module 21
ENSG00000235904	module 21
ENSG00000256234	module 21
ENSG00000276116	module 21
ENSG00000253959	module 21
ENSG00000232453	module 21
ENSG00000261512	module 21
ENSG00000248161	module 21
ENSG00000272944	module 21
ENSG00000237463	module 21
ENSG00000178977	module 21
ENSG00000082929	module 21
ENSG00000250091	module 21
ENSG00000254531	module 21
ENSG00000180458	module 21
ENSG00000258048	module 21
ENSG00000223478	module 21
ENSG00000267317	module 21
ENSG00000248596	module 21
ENSG00000223403	module 21
ENSG00000261655	module 21
ENSG00000224032	module 21
ENSG00000257913	module 21
ENSG00000260686	module 21
ENSG00000272273	module 21
ENSG00000249082	module 21
ENSG00000186235	module 21
ENSG00000244541	module 21
ENSG00000230387	module 21

ENSG00000281376	module 21
ENSG00000259831	module 21
ENSG00000276223	module 21
ENSG00000237009	module 21
ENSG00000269906	module 21
ENSG00000233070	module 21
ENSG00000254501	module 21
ENSG00000254473	module 21
ENSG00000203644	module 21
ENSG00000241135	module 21
ENSG00000204380	module 21
ENSG00000267454	module 21
ENSG00000269386	module 21
ENSG00000254027	module 21
ENSG00000237413	module 21
ENSG00000228295	module 21
ENSG00000205106	module 21
ENSG00000242396	module 21
ENSG00000247796	module 21
ENSG00000233421	module 21
ENSG00000278970	module 21
ENSG00000245571	module 21
ENSG00000234390	module 21
ENSG00000265666	module 21
ENSG00000229334	module 21
ENSG00000277879	module 21
ENSG00000271787	module 21
ENSG00000258616	module 21
ENSG00000238005	module 21
ENSG00000255345	module 21
ENSG00000227486	module 21
ENSG00000268038	module 21
ENSG00000247081	module 21
ENSG00000273311	module 21
ENSG00000273409	module 21
ENSG00000231482	module 21
ENSG00000244791	module 21
ENSG00000267593	module 21
ENSG00000223799	module 21
ENSG00000225062	module 21
ENSG00000251141	module 21
ENSG00000260941	module 21
ENSG00000233554	module 21
ENSG00000214691	module 21

ENSG00000259623	module 21
ENSG00000253738	module 21
ENSG00000274993	module 21
ENSG00000267040	module 21
ENSG00000228775	module 21
ENSG00000236714	module 21
ENSG00000180769	module 21
ENSG00000276012	module 21
ENSG00000236914	module 21
ENSG00000165511	module 21
ENSG00000267174	module 21
ENSG00000250131	module 21
ENSG00000254885	module 21
ENSG00000246528	module 21
ENSG00000251095	module 21
ENSG00000260423	module 21
ENSG00000259583	module 21
ENSG00000238273	module 21
ENSG00000176753	module 21
ENSG00000177112	module 21
ENSG00000224914	module 21
ENSG00000224043	module 21
ENSG00000236833	module 21
ENSG00000258636	module 21
ENSG00000228509	module 21
ENSG00000196204	module 21
ENSG00000258092	module 21
ENSG00000267058	module 21
ENSG00000242242	module 21
ENSG00000255435	module 21
ENSG00000268061	module 21
ENSG00000272129	module 21
ENSG00000255114	module 21
ENSG00000273838	module 21
ENSG00000247925	module 21
ENSG00000234807	module 21
ENSG00000189419	module 21
ENSG00000258122	module 21
ENSG00000246560	module 21
ENSG00000261167	module 21
ENSG00000231171	module 21
ENSG00000259343	module 21
ENSG00000249364	module 21
ENSG00000245105	module 21

ENSG00000251165	module 21
ENSG00000260583	module 21
ENSG00000225611	module 21
ENSG00000238133	module 21
ENSG00000235848	module 21
ENSG00000244558	module 21
ENSG00000268095	module 21
ENSG00000247853	module 21
ENSG00000274026	module 21
ENSG00000254008	module 21
ENSG00000255495	module 21
ENSG00000205861	module 21
ENSG00000277763	module 21
ENSG00000244953	module 21
ENSG00000261441	module 21
ENSG00000178248	module 21
ENSG00000261613	module 21
ENSG00000217702	module 21
ENSG00000225298	module 21
ENSG00000226496	module 21
ENSG00000136315	module 21
ENSG00000237422	module 21
ENSG00000228013	module 21
ENSG00000259052	module 21
ENSG00000250986	module 21
ENSG00000239513	module 21
ENSG00000214146	module 21
ENSG00000259347	module 21
ENSG00000275620	module 21
ENSG00000204625	module 21
ENSG00000231890	module 21
ENSG00000273784	module 21
ENSG00000257114	module 21
ENSG00000276412	module 21
ENSG00000248774	module 21
ENSG00000230725	module 21
ENSG00000242628	module 21
ENSG00000231453	module 21
ENSG00000256732	module 21
ENSG00000241684	module 21
ENSG00000245870	module 21
ENSG00000255052	module 21
ENSG00000264727	module 21
ENSG00000205293	module 21

ENSG00000254204	module 21
ENSG00000233067	module 21
ENSG00000229694	module 21
ENSG00000267696	module 21
ENSG00000267449	module 21
ENSG00000261773	module 21
ENSG00000224023	module 21
ENSG00000251359	module 21
ENSG00000267890	module 21
ENSG00000260339	module 21
ENSG00000224854	module 21
ENSG00000235652	module 21
ENSG00000278996	module 21
ENSG00000267134	module 21
ENSG00000231010	module 21
ENSG00000236208	module 21
ENSG00000250846	module 21
ENSG00000212766	module 21
ENSG00000275120	module 21
ENSG00000266289	module 21
ENSG00000261296	module 21
ENSG00000225555	module 21
ENSG00000234661	module 21
ENSG00000239332	module 21
ENSG00000253133	module 21
ENSG00000235661	module 21
ENSG00000236719	module 21
ENSG00000261757	module 21
ENSG00000264026	module 21
ENSG00000255794	module 21
ENSG00000264859	module 21
ENSG00000273073	module 21
ENSG00000259359	module 21
ENSG00000260269	module 21
ENSG00000255120	module 21
ENSG00000260979	module 21
ENSG00000228624	module 21
ENSG00000219665	module 21
ENSG00000259869	module 21
ENSG00000245857	module 21
ENSG00000233009	module 21
ENSG00000254202	module 21
ENSG00000257137	module 21
ENSG00000170161	module 21

ENSG00000253893	module 21
ENSG00000267424	module 21
ENSG00000226272	module 21
ENSG00000227456	module 21
ENSG00000246375	module 21
ENSG00000224174	module 21
ENSG00000241280	module 21
ENSG00000260249	module 21
ENSG00000226764	module 21
ENSG00000256020	module 21
ENSG00000242029	module 21
ENSG00000225655	module 21
ENSG00000272817	module 21
ENSG00000224995	module 21
ENSG00000215374	module 21
ENSG00000237928	module 21
ENSG00000226308	module 21
ENSG00000225206	module 21
ENSG00000183822	module 21
ENSG00000260578	module 21
ENSG00000230333	module 21
ENSG00000280924	module 21
ENSG00000226266	module 21
ENSG00000250658	module 21
ENSG00000214803	module 21
ENSG00000272755	module 21
ENSG00000231943	module 21
ENSG00000235407	module 21
ENSG00000227888	module 21
ENSG00000255693	module 21
ENSG00000248309	module 21
ENSG00000262061	module 21
ENSG00000261453	module 21
ENSG00000250107	module 21
ENSG00000253295	module 21
ENSG00000259514	module 21
ENSG00000238184	module 21
ENSG00000167046	module 21
ENSG00000267222	module 21
ENSG00000253327	module 21
ENSG00000232560	module 21
ENSG00000229153	module 21
ENSG00000248079	module 21
ENSG00000236024	module 21

ENSG00000267028	module 21
ENSG00000274874	module 21
ENSG00000235659	module 21
ENSG00000236366	module 21
ENSG00000231689	module 21
ENSG00000258586	module 21
ENSG00000234199	module 21
ENSG00000274718	module 21
ENSG00000260469	module 21
ENSG00000249252	module 21
ENSG00000267308	module 21
ENSG00000274859	module 21
ENSG00000236094	module 21
ENSG00000267984	module 21
ENSG00000254905	module 21
ENSG00000261229	module 21
ENSG00000260947	module 21
ENSG00000275356	module 21
ENSG00000261795	module 21
ENSG00000235989	module 21
ENSG00000231728	module 21
ENSG00000234452	module 21
ENSG00000227811	module 21
ENSG00000257433	module 21
ENSG00000254447	module 21
ENSG00000214043	module 21
ENSG00000267216	module 21
ENSG00000260070	module 21
ENSG00000261487	module 21
ENSG00000231934	module 21
ENSG00000183674	module 21
ENSG00000271743	module 21
ENSG00000225493	module 21
ENSG00000279442	module 21
ENSG00000260246	module 21
ENSG00000261359	module 21
ENSG00000267512	module 21
ENSG00000259149	module 21
ENSG00000281852	module 21
ENSG00000235545	module 21
ENSG00000269037	module 21
ENSG00000241357	module 21
ENSG00000264985	module 21
ENSG00000256150	module 21

ENSG00000255850	module 21
ENSG00000233354	module 21
ENSG00000236548	module 21
ENSG00000229414	module 21
ENSG00000233262	module 21
ENSG00000261070	module 21
ENSG00000271980	module 21
ENSG00000240770	module 21
ENSG00000177757	module 21
ENSG00000249275	module 21
ENSG00000267328	module 21
ENSG00000236301	module 21
ENSG00000228157	module 21
ENSG00000259115	module 21
ENSG00000136275	module 21
ENSG00000267009	module 21
ENSG00000225028	module 21
ENSG00000224715	module 21
ENSG00000275239	module 21
ENSG00000250696	module 21
ENSG00000270074	module 21
ENSG00000256603	module 21
ENSG00000280441	module 21
ENSG00000246331	module 21
ENSG00000272396	module 21
ENSG00000259370	module 21
ENSG00000266921	module 21
ENSG00000125804	module 21
ENSG00000177340	module 21
ENSG00000258121	module 21
ENSG00000188525	module 21
ENSG00000249695	module 21
ENSG00000237862	module 21
ENSG00000235410	module 21
ENSG00000280639	module 21
ENSG00000267105	module 21
ENSG00000279317	module 21
ENSG00000261113	module 21
ENSG00000234817	module 21
ENSG00000262728	module 21
ENSG00000279484	module 21
ENSG00000267535	module 21
ENSG00000248964	module 21
ENSG00000247970	module 21

ENSG00000229236	module 21
ENSG00000248550	module 21
ENSG00000231944	module 21
ENSG00000224661	module 21
ENSG00000268199	module 21
ENSG00000230140	module 21
ENSG00000232762	module 21
ENSG00000267104	module 21
ENSG00000233746	module 21
ENSG00000254551	module 21
ENSG00000253138	module 21
ENSG00000224308	module 21
ENSG00000278419	module 21
ENSG00000280273	module 21
ENSG00000227215	module 21
ENSG00000237714	module 21
ENSG00000261257	module 21
ENSG00000237058	module 21
ENSG00000254226	module 21
ENSG00000256632	module 21
ENSG00000233153	module 21
ENSG00000259907	module 21
ENSG00000224919	module 21
ENSG00000233926	module 21
ENSG00000256494	module 21
ENSG00000259946	module 21
ENSG00000260677	module 21
ENSG00000224356	module 21
ENSG00000257259	module 21
ENSG00000259970	module 21
ENSG00000178457	module 21
ENSG00000273805	module 21
ENSG00000180869	module 21
ENSG00000182586	module 21
ENSG00000269139	module 21
ENSG00000275389	module 21
ENSG00000269460	module 21
ENSG00000224342	module 21
ENSG00000269983	module 21
ENSG00000267160	module 21
ENSG00000261026	module 21
ENSG00000256034	module 21
ENSG00000270207	module 21
ENSG00000253875	module 21

ENSG00000234384	module 21
ENSG00000277543	module 21
ENSG00000234693	module 21
ENSG00000203897	module 21
ENSG00000231666	module 21
ENSG00000261334	module 21
ENSG00000280136	module 21
ENSG00000253356	module 21
ENSG00000254377	module 21
ENSG00000233008	module 21
ENSG00000204250	module 21
ENSG00000275088	module 21
ENSG00000228016	module 21
ENSG00000225050	module 21
ENSG00000227602	module 21
ENSG00000254654	module 21
ENSG00000225446	module 21
ENSG00000240423	module 21
ENSG00000164621	module 21
ENSG00000237787	module 21
ENSG00000260206	module 21
ENSG00000235641	module 21
ENSG00000279151	module 21
ENSG00000246022	module 21
ENSG00000260021	module 21
ENSG00000267070	module 21
ENSG00000254119	module 21
ENSG00000093100	module 21
ENSG00000234435	module 21
ENSG00000249109	module 21
ENSG00000185847	module 21
ENSG00000226047	module 21
ENSG00000260676	module 21
ENSG00000259546	module 21
ENSG00000264215	module 21
ENSG00000261292	module 21
ENSG00000223975	module 21
ENSG00000207751	module 21
ENSG00000229257	module 21
ENSG00000254295	module 21
ENSG00000271776	module 21
ENSG00000237357	module 21
ENSG00000260550	module 21
ENSG00000254813	module 21

ENSG00000224382	module 21
ENSG00000254139	module 21
ENSG00000234520	module 21
ENSG00000230417	module 21
ENSG00000246130	module 21
ENSG00000265168	module 21
ENSG00000185594	module 21
ENSG00000227480	module 21
ENSG00000216560	module 21
ENSG00000260094	module 21
ENSG00000268324	module 21
ENSG00000228215	module 21
ENSG00000259245	module 21
ENSG00000281732	module 21
ENSG00000179219	module 21
ENSG00000263644	module 21
ENSG00000222004	module 21
