# Supplementary material for: Single-cell RNA sequencing integrated with bulk RNA sequencing analysis identifies a tumor immune microenvironment-related lncRNA signature in lung adenocarcinoma
Source: BMC Biol. 2024 Mar 22;22:69. doi: 10.1186/s12915-024-01866-5 (PMC10960411; doi:10.1186/s12915-024-01866-5)
Supplement: Supplementary file 11 — Additional file 11: Table S5. Prognostic value of 33 lncRNAs in six cohorts. [file 12915_2024_1866_MOESM11_ESM.pdf]

Table S5. Prognostic value of 33 lncRNAs in six cohorts.

	TCGA_LUAD	GSE72094	GSE50081	GSE31210	GSE30219	GSE3141
ENSG00000239911	-0.017932003	-0.130297033	-5.03E-05	-0.038940232	-0.02177415	-0.278164886
ENSG00000229334	0.002979702	0.008133068	0.054833612	0.05743321	0.012471216	-0.830576554
ENSG00000225342	-0.062839461	-5.31E-07	-0.121418733	-0.063000126	-0.12126711	-0.160378856
ENSG00000278419	0.556552524	0.002774749	0.121807395	0.001237563	0.052857209	0.014826485
ENSG00000253859	0.060376529	-0.673442502	0.042487015	0.027066357	0.170281193	0.10237661
ENSG00000241684	-0.037086248	-0.174079703	-0.126652537	-0.000661656	-0.078712961	-0.362326414
ENSG00000225329	-0.03437707	-0.006976432	-0.030549279	-0.000823541	-0.015464449	-0.453039105
ENSG00000255399	-0.037628899	-0.020868847	-0.028170796	-0.002051448	-0.157214417	0.382409174
ENSG00000241490	-0.091195766	-0.031117797	-0.013467277	-0.120498704	-0.03054032	-0.396766275
ENSG00000260740	-0.031011365	-0.001029173	-0.026045559	-0.175904282	-0.020225078	-0.774413717
ENSG00000261061	0.004165875	0.00098789	0.005903676	0.001052365	0.00453032	-0.894568383
ENSG00000227544	-0.002451207	-0.000253142	0.808586261	-0.083964237	-0.164068746	-0.045656142
ENSG00000232046	-0.084037442	-0.105424194	-0.064414106	-0.00023043	-0.029192493	-0.212904191
ENSG00000280206	0.018565837	0.130148481	0.062818885	0.014297777	0.052579405	0.01096345
ENSG00000248801	-0.039026843	-0.00017819	-0.062495209	-0.03763293	-0.191737193	-0.032907994
ENSG00000278709	0.000680919	0.004650684	0.01560744	0.001677498	0.003923762	0.172603083
ENSG00000250519	0.586937575	0.000275728	0.161667601	0.003910479	0.105958345	0.00807349
ENSG00000245534	0.055649563	-9.87E-06	-0.155868752	-0.007605174	-0.055924756	-0.013486063
ENSG00000167912	-0.16914564	-4.17E-05	-1.50E-05	-0.037708861	-0.022162122	-0.306769379
ENSG00000259974	-0.017548219	-0.00024815	-0.003987847	-0.10340017	-0.082413628	-0.345410578
ENSG00000224189	-0.113465595	-1.04E-07	-0.019646385	-0.03253954	-0.117419436	-0.183367989
ENSG00000260244	-0.029146927	-0.007502986	-0.068057082	-0.011751986	-0.084247539	-0.504892966
ENSG00000261113	-0.699773576	-0.172126601	-0.001771558	-0.002210868	-0.000455227	-0.002890474
ENSG00000229891	-0.026065864	-0.021113199	-0.088270933	-0.029443243	-0.116264152	-0.009382994
ENSG00000280721	0.693652848	0.000352938	0.009251145	0.000850746	0.00763144	0.008059032
ENSG00000237523	9.82E-05	0.00014179	0.072848333	0.000163686	0.016167502	-0.131989319
ENSG00000225383	-0.013124684	-0.001181487	-0.011486452	-0.067808315	-0.084723488	-0.951677596
ENSG00000180769	0.616070612	-0.000394681	-0.176032722	-0.000138887	-0.074435275	-0.052623342
ENSG00000276012	-0.605851901	-0.161816257	-0.135326055	-0.10200226	-0.021337674	-0.185162287
ENSG00000233093	-0.000931638	-0.000925158	-0.061922082	-0.412195642	-0.131956858	-0.008473294
ENSG00000224167	0.02023365	0.171547852	0.092853446	0.001613699	1.01E-06	-0.451645728
ENSG00000281376	0.000506043	0.002604672	0.000978963	-0.348593898	0.01716598	0.000237297
ENSG00000258092	0.040353633	9.65E-06	0.056828471	0.080856019	0.032483926	0.921002218
