# Supplementary material for: Single-cell RNA sequencing integrated with bulk RNA sequencing analysis identifies a tumor immune microenvironment-related lncRNA signature in lung adenocarcinoma
Source: BMC Biol. 2024 Mar 22;22:69. doi: 10.1186/s12915-024-01866-5 (PMC10960411; doi:10.1186/s12915-024-01866-5)
Supplement: Supplementary file 12 — Additional file 12: Table S6. Formula for the calculation of TRLS. [file 12915_2024_1866_MOESM12_ESM.pdf]

Table S6. Formula for the calculation of TRLS.

TRLS = $\sum_{i=1}^{33} gene(i) * coef(i)$, where gene(i), coef(i) represented gene expression level, and the coefficient index. Specific gene names and coefficient indexes are shown in the table below.

gene	coef
ENSG00000239911	-0.030035653
ENSG00000225342	0.00130933
ENSG00000241684	-0.019165415
ENSG00000225329	-0.007224547
ENSG00000255399	-0.013733801
ENSG00000241490	-0.019967817
ENSG00000260740	-0.025677208
ENSG00000227544	-0.036869042
ENSG00000232046	-0.023250559
ENSG00000248801	-0.000839909
ENSG00000245534	0.030187154
ENSG00000167912	-0.00552471
ENSG00000259974	-0.020703545
ENSG00000224189	0.00310215
ENSG00000260244	-0.010134104
ENSG00000261113	0.005681313
ENSG00000229891	-0.020038513
ENSG00000225383	-0.010819976
ENSG00000180769	0.040579047
ENSG00000276012	0.009635627
ENSG00000233093	-0.047521074
ENSG00000229334	0.035826742
ENSG00000278419	0.005575795
ENSG00000253859	0.031837394
ENSG00000261061	0.034894752
ENSG00000280206	0.027714215
ENSG00000278709	0.054723268
ENSG00000250519	-0.003296945
ENSG00000280721	0.005711753
ENSG00000237523	0.05978065
ENSG00000224167	0.023710505
ENSG00000281376	0.047965639
ENSG00000258092	0.028184396
