# Supplementary material for: Single-cell RNA sequencing integrated with bulk RNA sequencing analysis identifies a tumor immune microenvironment-related lncRNA signature in lung adenocarcinoma
Source: BMC Biol. 2024 Mar 22;22:69. doi: 10.1186/s12915-024-01866-5 (PMC10960411; doi:10.1186/s12915-024-01866-5)
Supplement: Supplementary file 13 — Additional file 13: Table S7. Individual TRLS for patients in six cohorts. [file 12915_2024_1866_MOESM13_ESM.pdf]

Table S7. Individual TRLS for patients in six cohorts.

TGGA-LUAD	TRLS	GSE72094	TRLS	GSE50081	TRLS
TCGA-05-4249-01A	-0.074376144	GSM1854798	-0.017047037	GSM1213670	-0.167316696
TCGA-05-4250-01A	0.495212425	GSM1854799	0.21289003	GSM1213671	-0.313922279
TCGA-05-4382-01A	0.237594702	GSM1854800	0.296939355	GSM1213672	0.023143228
TCGA-05-4384-01A	-0.222234577	GSM1854801	-0.291698701	GSM1213673	0.198153178
TCGA-05-4389-01A	-0.040211687	GSM1854802	0.254238257	GSM1213676	0.15814
TCGA-05-4390-01A	0.056546133	GSM1854803	-0.040735712	GSM1213677	0.250127431
TCGA-05-4396-01A	-0.235055066	GSM1854804	0.101734935	GSM1213678	-0.010476147
TCGA-05-4397-01A	0.318374359	GSM1854805	0.470478057	GSM1213679	-0.29540448
TCGA-05-4398-01A	0.223280793	GSM1854806	-0.172326318	GSM1213680	0.300541081
TCGA-05-4402-01A	0.068134577	GSM1854807	-0.078593526	GSM1213681	0.059691636
TCGA-05-4403-01A	0.109761098	GSM1854808	-0.362801061	GSM1213682	0.128316049
TCGA-05-4405-01A	-0.199578147	GSM1854809	-0.249494457	GSM1213683	-0.018092284
TCGA-05-4415-01A	0.814997668	GSM1854810	0.224595912	GSM1213685	-0.481157365
TCGA-05-4417-01A	-0.09246	GSM1854811	0.124559602	GSM1213686	0.31294606
TCGA-05-4418-01A	0.389441311	GSM1854812	-0.245508789	GSM1213687	-0.344034198
TCGA-05-4420-01A	0.12040407	GSM1854813	-0.502889823	GSM1213688	-0.145353787
TCGA-05-4422-01A	-0.182777569	GSM1854814	0.065834326	GSM1213689	-0.408874496
TCGA-05-4424-01A	-0.09069399	GSM1854815	0.123549355	GSM1213690	-0.045265617
TCGA-05-4425-01A	-0.059277934	GSM1854816	-0.123563392	GSM1213691	0.121286531
TCGA-05-4426-01A	0.004709942	GSM1854817	0.336842132	GSM1213692	-0.479245314
TCGA-05-4427-01A	0.332306343	GSM1854818	0.141000932	GSM1213693	-0.09354217
TCGA-05-4430-01A	0.062002597	GSM1854819	-0.059023296	GSM1213694	-0.006395278
TCGA-05-4432-01A	0.001550996	GSM1854820	0.000317352	GSM1213695	0.198674392
TCGA-05-4433-01A	0.103623656	GSM1854821	-0.129328346	GSM1213696	-0.116226338
TCGA-05-4434-01A	-0.108919473	GSM1854822	-0.100545917	GSM1213697	-0.479633821
TCGA-05-5420-01A	-0.028332214	GSM1854823	0.355079598	GSM1213698	0.158491457
TCGA-05-5423-01A	-0.350683191	GSM1854825	-0.027247549	GSM1213699	-0.331776262
TCGA-05-5425-01A	-0.073229987	GSM1854826	-0.340312031	GSM1213700	0.10777221
TCGA-05-5428-01A	0.025151669	GSM1854827	0.315481951	GSM1213702	0.166037128
TCGA-05-5429-01A	0.431994701	GSM1854828	0.039701954	GSM1213703	-0.319002587
TCGA-05-5715-01A	-0.014178126	GSM1854829	0.307857481	GSM1213704	0.122377921
TCGA-35-3615-01A	-0.31507076	GSM1854830	0.127279154	GSM1213707	-0.063982733
TCGA-35-4122-01A	0.202030292	GSM1854831	0.505488894	GSM1213710	0.124058243
TCGA-35-4123-01A	0.393148668	GSM1854832	-0.364302379	GSM1213715	0.56352734
TCGA-35-5375-01A	-0.252468488	GSM1854833	-0.393115188	GSM1213716	0.152161214
TCGA-38-4625-01A	0.213413978	GSM1854834	-0.078206012	GSM1213717	0.379437317
TCGA-38-4626-01A	-0.24502706	GSM1854835	-0.030756387	GSM1213720	-0.013066165
TCGA-38-4627-01A	-0.011111107	GSM1854836	-0.126191204	GSM1213722	0.227761006
TCGA-38-4628-01A	0.05185054	GSM1854837	-0.032820256	GSM1213723	0.301274919
TCGA-38-4629-01A	0.453352564	GSM1854838	0.229256231	GSM1213724	-0.334087636
TCGA-38-4630-01A	0.260384666	GSM1854839	-0.36125656	GSM1213726	0.072112295
TCGA-38-4631-01A	0.650720615	GSM1854840	-0.341064564	GSM1213727	-0.04779766

TCGA-38-4632-01A	0.240225803	GSM1854841	0.407155534	GSM1213728	-0.268274503
TCGA-38-6178-01A	-0.188240822	GSM1854842	-0.348813532	GSM1213730	0.377054921
TCGA-38-7271-01A	-0.311584945	GSM1854843	-0.377420259	GSM1213733	0.146840182
TCGA-38-A44F-01A	-0.182097725	GSM1854844	-0.226074555	GSM1213734	-0.040324833
TCGA-44-2655-01A	-0.22316916	GSM1854846	-0.095122774	GSM1213735	-0.049342594
TCGA-44-2656-01A	0.083927775	GSM1854847	-0.185784783	GSM1213736	0.409589277
TCGA-44-2657-01A	-0.358101231	GSM1854848	-0.256126839	GSM1213737	-0.118395138
TCGA-44-2659-01A	-0.338581746	GSM1854849	-0.199641849	GSM1213738	-0.192538773
TCGA-44-2661-01A	-0.53581083	GSM1854850	0.049471234	GSM1213739	0.043967976
TCGA-44-2662-01A	0.275279959	GSM1854851	-0.374663754	GSM1213740	-0.092283461
TCGA-44-2665-01A	0.105400111	GSM1854852	-0.564395255	GSM1213741	-0.152606008
TCGA-44-2666-01A	-0.195126382	GSM1854853	0.086940379	GSM1213742	0.24374833
TCGA-44-2668-01A	0.496273976	GSM1854854	-0.49169849	GSM1213743	-0.070413949
TCGA-44-3396-01A	0.078599268	GSM1854855	0.241260371	GSM1213746	-0.119612931
TCGA-44-3398-01A	-0.191074924	GSM1854856	0.160650536	GSM1213747	-0.0670589
TCGA-44-3917-01A	-0.140114982	GSM1854857	-0.267417687	GSM1213749	-0.028216849
TCGA-44-3918-01A	-0.069772275	GSM1854859	-0.154729851	GSM1213750	0.264530937
TCGA-44-3919-01A	-0.117185592	GSM1854860	0.189435615	GSM1213751	-0.188516939
TCGA-44-4112-01A	-0.021958598	GSM1854861	0.241982615	GSM1213752	-0.088761293
TCGA-44-5643-01A	0.157229118	GSM1854862	-0.383976399	GSM1213753	-0.391843771
TCGA-44-5644-01A	0.151530438	GSM1854863	-0.139019398	GSM1213754	0.160440062
TCGA-44-5645-01A	-0.340277683	GSM1854864	-0.275446846	GSM1213756	-0.238907975
TCGA-44-6145-01A	-0.002827936	GSM1854865	0.034258692	GSM1213757	-0.019478899
TCGA-44-6146-01A	0.073009642	GSM1854866	0.293733267	GSM1213758	-0.430774059
TCGA-44-6147-01A	-0.242945814	GSM1854867	-0.050406425	GSM1213759	0.159373734
TCGA-44-6148-01A	-0.453888944	GSM1854868	0.540969656	GSM1213760	0.147817098
TCGA-44-6774-01A	-0.013819215	GSM1854869	-0.174519095	GSM1213761	-0.330059411
TCGA-44-6775-01A	0.125280264	GSM1854870	0.146453723	GSM1213762	0.325486255
TCGA-44-6776-01A	-0.178037436	GSM1854871	-0.035415065	GSM1213763	-0.063328025
TCGA-44-6777-01A	-0.128810732	GSM1854872	0.215595823	GSM1213764	0.00116652
TCGA-44-6778-01A	-0.289953062	GSM1854873	0.185479261	GSM1213768	0.011808336
TCGA-44-6779-01A	0.352387304	GSM1854874	-0.080495479	GSM1213769	-0.079334956
TCGA-44-7659-01A	-0.227660527	GSM1854875	0.014013821	GSM1213770	0.090174109
TCGA-44-7660-01A	0.179342112	GSM1854876	0.041231375	GSM1213773	-0.277831744
TCGA-44-7661-01A	0.230509725	GSM1854877	-0.129358877	GSM1213774	-0.137219896
TCGA-44-7662-01A	0.1921837	GSM1854879	-0.179704602	GSM1213775	-0.070448856
TCGA-44-7667-01A	0.121222021	GSM1854880	0.134582549	GSM1213777	0.005919492
TCGA-44-7669-01A	0.17611819	GSM1854881	0.188246506	GSM1213779	-0.336044937
TCGA-44-7670-01A	0.287032912	GSM1854882	0.071728258	GSM1213780	0.0792687
TCGA-44-7671-01A	0.071010726	GSM1854883	-0.118343952	GSM1213781	0.127286472
TCGA-44-7672-01A	0.09461493	GSM1854884	0.174139146	GSM1213782	0.01893099
TCGA-44-8117-01A	0.285912954	GSM1854885	-0.033755338	GSM1213783	0.255547099
TCGA-44-8119-01A	0.238422379	GSM1854886	0.627367946	GSM1213784	0.030281307
TCGA-44-8120-01A	0.196436556	GSM1854887	-0.032891487	GSM1213785	-0.101757405

TCGA-44-A479-01A	-0.049744524	GSM1854888	-0.158679677	GSM1213787	-0.19457588
TCGA-44-A47A-01A	-0.173222349	GSM1854889	0.003262938	GSM1213788	-0.095120748
TCGA-44-A47B-01A	0.06937263	GSM1854890	0.031357299	GSM1213792	0.159317867
TCGA-44-A47G-01A	-0.262901277	GSM1854891	0.465482577	GSM1213793	0.266118481
TCGA-44-A4SS-01A	0.090095715	GSM1854892	0.082636735	GSM1213794	-0.430227954
TCGA-44-A4SU-01A	-0.11129691	GSM1854893	0.114361831	GSM1213795	-0.422669275
TCGA-49-4486-01A	-0.293213712	GSM1854895	0.436750771	GSM1213797	-0.489357252
TCGA-49-4487-01A	0.045512636	GSM1854896	-0.02468712	GSM1213798	-0.179112299
TCGA-49-4488-01A	0.268780672	GSM1854897	0.265509482	GSM1213800	-0.01069821
TCGA-49-4490-01A	-0.036770022	GSM1854898	-0.264161616	GSM1213801	0.134768279
TCGA-49-4494-01A	0.12681136	GSM1854899	0.145942675	GSM1213803	0.341919184
TCGA-49-4501-01A	-0.147725365	GSM1854900	-0.323565224	GSM1213804	-0.108523006
TCGA-49-4505-01A	0.04943787	GSM1854902	-0.376882864	GSM1213805	0.339385417
TCGA-49-4506-01A	0.223787957	GSM1854903	0.270271003	GSM1213806	0.279868122
TCGA-49-4507-01A	0.318291671	GSM1854904	-0.252144934	GSM1213807	0.282297691
TCGA-49-4510-01A	-0.214190927	GSM1854906	0.031236844	GSM1213808	-0.472152822
TCGA-49-4512-01A	0.109236836	GSM1854907	-0.026110534	GSM1213809	-0.34718245
TCGA-49-4514-01A	-0.052885897	GSM1854908	0.012338188	GSM1213810	-0.273400972
TCGA-49-6742-01A	0.275351105	GSM1854909	0.20426271	GSM1213811	-0.12178059
TCGA-49-6743-01A	0.381479923	GSM1854910	-0.209647507	GSM1213812	0.261374623
TCGA-49-6744-01A	-0.24433375	GSM1854911	0.314101419	GSM1213814	0.022365869
TCGA-49-6745-01A	0.272616268	GSM1854913	-0.173624266	GSM1213816	0.448072745
TCGA-49-6761-01A	0.170199566	GSM1854915	0.025953413	GSM1213818	0.069283598
TCGA-49-6767-01A	0.623334465	GSM1854916	0.244455839	GSM1213819	0.320193947
TCGA-49-AAQV-01A	-0.121353052	GSM1854917	-0.08340891	GSM1213820	0.356841006
TCGA-49-AAR0-01A	-0.247213648	GSM1854918	-0.06650228	GSM1213824	0.128001877
TCGA-49-AAR2-01A	-0.026610448	GSM1854919	0.045613783	GSM1213825	-0.357876604
TCGA-49-AAR3-01A	0.388799015	GSM1854920	-0.250607914	GSM1213826	0.52890489
TCGA-49-AAR4-01A	0.144699633	GSM1854921	0.439686548	GSM1213827	0.134876459
TCGA-49-AAR9-01A	0.993395495	GSM1854922	-0.388449849	GSM1213831	-0.318766789
TCGA-49-AARE-01A	0.46180385	GSM1854923	0.140210502	GSM1213833	-0.074390348
TCGA-49-AARN-01A	0.053154135	GSM1854924	-0.289877602	GSM1213834	-0.010257005
TCGA-49-AARO-01A	0.18783936	GSM1854925	-0.214958173	GSM1213836	0.287125898
TCGA-49-AARQ-01A	0.007567523	GSM1854926	0.016339505	GSM1213837	0.281202149
TCGA-49-AARR-01A	-0.372501424	GSM1854927	-0.022525859	GSM1213840	0.471877148
TCGA-4B-A93V-01A	0.089440415	GSM1854928	-0.120600846	GSM1213844	-0.074272301
TCGA-50-5044-01A	0.44898921	GSM1854929	-0.059035119	GSM1213845	0.49404638
TCGA-50-5045-01A	-0.244917907	GSM1854930	-0.244914811	GSM1213846	-0.082184761
TCGA-50-5049-01A	-0.156260513	GSM1854931	-0.368856914	GSM1213847	-0.4758566
TCGA-50-5051-01A	0.023298778	GSM1854932	0.02933548	GSM1213848	0.344954037
TCGA-50-5055-01A	-0.288796369	GSM1854933	0.140275587	GSM1213849	0.028377185
TCGA-50-5066-01A	-0.074609933	GSM1854934	-0.317748046	--	--
TCGA-50-5068-01A	-0.499905873	GSM1854935	0.131998286	--	--
TCGA-50-5072-01A	0.693392866	GSM1854936	-0.161842533	--	--

TCGA-50-5930-01A	0.309271009	GSM1854937	0.000655253	--	--
TCGA-50-5931-01A	0.075693448	GSM1854938	0.509458474	--	--
TCGA-50-5932-01A	0.034923983	GSM1854940	-0.055662092	--	--
TCGA-50-5933-01A	0.09619574	GSM1854941	0.200862446	--	--
TCGA-50-5935-01A	-0.19115722	GSM1854942	0.359698837	--	--
TCGA-50-5936-01A	0.233600374	GSM1854944	0.117091073	--	--
TCGA-50-5939-01A	0.376816181	GSM1854945	0.169388342	--	--
TCGA-50-5941-01A	-0.010101441	GSM1854947	0.579642765	--	--
TCGA-50-5942-01A	-0.481668671	GSM1854948	-0.014612709	--	--
TCGA-50-5944-01A	-0.209979211	GSM1854950	-0.17000348	--	--
TCGA-50-5946-01A	0.522677084	GSM1854951	-0.149966301	--	--
TCGA-50-6590-01A	0.269891659	GSM1854952	-0.155225636	--	--
TCGA-50-6591-01A	0.710446717	GSM1854953	-0.293928623	--	--
TCGA-50-6592-01A	0.606343584	GSM1854954	0.456147655	--	--
TCGA-50-6593-01A	-0.07307603	GSM1854955	-0.176122158	--	--
TCGA-50-6594-01A	0.64634144	GSM1854956	0.232676998	--	--
TCGA-50-6595-01A	0.426051953	GSM1854957	0.057038033	--	--
TCGA-50-6597-01A	-0.25733835	GSM1854958	-0.031229543	--	--
TCGA-50-6673-01A	-0.104598234	GSM1854960	0.140190712	--	--
TCGA-50-7109-01A	-0.187310145	GSM1854961	0.223959865	--	--
TCGA-50-8457-01A	-0.436591973	GSM1854962	-0.058789591	--	--
TCGA-50-8459-01A	0.214873202	GSM1854963	0.227956342	--	--
TCGA-50-8460-01A	-0.239257158	GSM1854964	0.266099555	--	--
TCGA-53-7624-01A	0.532433919	GSM1854965	0.116090743	--	--
TCGA-53-7626-01A	-0.162884193	GSM1854966	-0.272217837	--	--
TCGA-53-7813-01A	0.020595773	GSM1854967	0.144916074	--	--
TCGA-53-A4EZ-01A	-0.082017466	GSM1854969	0.314532801	--	--
TCGA-55-1592-01A	-0.234662723	GSM1854970	-0.110821602	--	--
TCGA-55-1594-01A	0.342290426	GSM1854971	-0.078847412	--	--
TCGA-55-1596-01A	0.286601812	GSM1854972	-0.238132141	--	--
TCGA-55-5899-01A	-0.086936393	GSM1854973	0.183671362	--	--
TCGA-55-6543-01A	-0.137578012	GSM1854974	-0.281986857	--	--
TCGA-55-6642-01A	-0.092135732	GSM1854975	0.158982796	--	--
TCGA-55-6712-01A	0.28325289	GSM1854977	-0.141812965	--	--
TCGA-55-6968-01A	0.108344989	GSM1854979	-0.347135395	--	--
TCGA-55-6970-01A	-0.156180803	GSM1854980	-0.593016549	--	--
TCGA-55-6971-01A	-0.239209566	GSM1854981	-0.070044463	--	--
TCGA-55-6972-01A	-0.01237259	GSM1854983	0.354927402	--	--
TCGA-55-6975-01A	0.562084159	GSM1854984	-0.036139452	--	--
TCGA-55-6978-01A	0.279213199	GSM1854985	-0.381526243	--	--
TCGA-55-6979-01A	0.055442859	GSM1854986	0.173530768	--	--
TCGA-55-6980-01A	-0.238982725	GSM1854987	-0.207932841	--	--
TCGA-55-6981-01A	0.122563239	GSM1854988	-0.170885252	--	--
TCGA-55-6982-01A	0.146326867	GSM1854989	-0.116483675	--	--

TCGA-55-6983-01A	-0.266843383	GSM1854990	-0.354856901	--	--
TCGA-55-6984-01A	0.197348297	GSM1854992	0.272770489	--	--
TCGA-55-6985-01A	0.045567634	GSM1854994	-0.298050819	--	--
TCGA-55-6986-01A	-0.191706407	GSM1854995	0.173984582	--	--
TCGA-55-6987-01A	-0.224753236	GSM1854996	0.139039371	--	--
TCGA-55-7227-01A	-0.173345497	GSM1854997	-0.12328839	--	--
TCGA-55-7281-01A	-0.058344102	GSM1854998	0.132579231	--	--
TCGA-55-7283-01A	-0.324902634	GSM1854999	-0.190710128	--	--
TCGA-55-7570-01A	0.659393985	GSM1855000	0.584368785	--	--
TCGA-55-7573-01A	-0.404667753	GSM1855001	-0.009812473	--	--
TCGA-55-7574-01A	-0.457165638	GSM1855003	-0.282582122	--	--
TCGA-55-7576-01A	0.194258976	GSM1855004	-0.117101232	--	--
TCGA-55-7724-01A	0.046145573	GSM1855005	0.007323623	--	--
TCGA-55-7725-01A	-0.444760443	GSM1855006	0.136988984	--	--
TCGA-55-7726-01A	0.353165424	GSM1855007	-0.189103118	--	--
TCGA-55-7727-01A	-0.006210782	GSM1855008	0.404060356	--	--
TCGA-55-7728-01A	-0.135071374	GSM1855009	-0.144553441	--	--
TCGA-55-7815-01A	-0.242719512	GSM1855010	0.225173671	--	--
TCGA-55-7816-01A	-0.201376245	GSM1855011	0.125256712	--	--
TCGA-55-7903-01A	0.083181826	GSM1855012	0.526190224	--	--
TCGA-55-7907-01A	0.205687807	GSM1855014	-0.153265898	--	--
TCGA-55-7910-01A	0.106053321	GSM1855015	-0.328561368	--	--
TCGA-55-7911-01A	-0.046367834	GSM1855016	0.39913062	--	--
TCGA-55-7914-01A	-0.019143116	GSM1855018	-0.06510279	--	--
TCGA-55-7994-01A	0.29069208	GSM1855019	0.074017711	--	--
TCGA-55-7995-01A	0.113115519	GSM1855020	0.292235512	--	--
TCGA-55-8085-01A	-0.167851884	GSM1855021	-0.004246601	--	--
TCGA-55-8087-01A	-0.408415915	GSM1855022	0.471139659	--	--
TCGA-55-8089-01A	0.393334205	GSM1855023	0.192903881	--	--
TCGA-55-8090-01A	0.045856677	GSM1855024	-0.044559625	--	--
TCGA-55-8091-01A	-0.006739809	GSM1855026	0.009471224	--	--
TCGA-55-8092-01A	0.008313255	GSM1855027	0.059381534	--	--
TCGA-55-8094-01A	0.056164267	GSM1855028	0.178665776	--	--
TCGA-55-8096-01A	0.0017227	GSM1855029	0.5249617	--	--
TCGA-55-8097-01A	-0.307864155	GSM1855031	-0.134056726	--	--
TCGA-55-8203-01A	-0.130513693	GSM1855032	0.017929534	--	--
TCGA-55-8204-01A	0.251545874	GSM1855033	0.444924363	--	--
TCGA-55-8205-01A	0.203445165	GSM1855034	-0.094313022	--	--
TCGA-55-8206-01A	-0.366473224	GSM1855035	-0.010922613	--	--
TCGA-55-8207-01A	-0.413871887	GSM1855036	-0.398056731	--	--
TCGA-55-8208-01A	-0.063406468	GSM1855037	0.308335653	--	--
TCGA-55-8299-01A	0.079289326	GSM1855038	0.301669613	--	--
TCGA-55-8301-01A	-0.048938249	GSM1855039	0.582590837	--	--
TCGA-55-8302-01A	0.368249048	GSM1855040	-0.062538326	--	--

TCGA-55-8505-01A	0.296835957	GSM1855041	-0.235959521	--	--
TCGA-55-8506-01A	0.174136844	GSM1855042	0.026410524	--	--
TCGA-55-8507-01A	-0.18647636	GSM1855043	-0.133997225	--	--
TCGA-55-8508-01A	-0.068880137	GSM1855044	-0.131433753	--	--
TCGA-55-8510-01A	-0.087942314	GSM1855045	-0.009813912	--	--
TCGA-55-8511-01A	0.025703082	GSM1855046	-0.201668437	--	--
TCGA-55-8512-01A	-0.264837291	GSM1855047	0.121088741	--	--
TCGA-55-8513-01A	-0.342736278	GSM1855048	-0.005300377	--	--
TCGA-55-8514-01A	-0.231700407	GSM1855049	0.049049116	--	--
TCGA-55-8614-01A	0.197994456	GSM1855050	0.03714923	--	--
TCGA-55-8615-01A	0.103641708	GSM1855051	-0.054716267	--	--
TCGA-55-8616-01A	-0.077325487	GSM1855052	0.116988038	--	--
TCGA-55-8619-01A	-0.285439325	GSM1855053	-0.378503633	--	--
TCGA-55-8620-01A	-0.236563611	GSM1855054	-0.479715685	--	--
TCGA-55-8621-01A	-0.255950668	GSM1855055	-0.360014126	--	--
TCGA-55-A48X-01A	-0.16406904	GSM1855056	-0.252680347	--	--
TCGA-55-A48Y-01A	0.091727177	GSM1855057	-0.067677746	--	--
TCGA-55-A48Z-01A	-0.011735509	GSM1855059	0.004098058	--	--
TCGA-55-A490-01A	-0.063749389	GSM1855060	0.398144855	--	--
TCGA-55-A491-01A	0.224932224	GSM1855061	-0.267148579	--	--
TCGA-55-A492-01A	-0.291374143	GSM1855062	0.486411766	--	--
TCGA-55-A493-01A	0.378697641	GSM1855063	-0.260513988	--	--
TCGA-55-A494-01A	0.093275376	GSM1855064	0.133636423	--	--
TCGA-55-A4DF-01A	0.030802963	GSM1855065	-0.022213257	--	--
TCGA-55-A4DG-01A	-0.260307587	GSM1855066	0.064133678	--	--
TCGA-55-A57B-01A	-0.200581872	GSM1855067	0.17473994	--	--
TCGA-62-8394-01A	0.228367863	GSM1855068	0.031588273	--	--
TCGA-62-8395-01A	-0.173778643	GSM1855069	-0.057763359	--	--
TCGA-62-8397-01A	-0.141394459	GSM1855070	-0.219389802	--	--
TCGA-62-8398-01A	0.323836287	GSM1855071	-0.106322429	--	--
TCGA-62-8399-01A	0.182904003	GSM1855072	-0.028539481	--	--
TCGA-62-8402-01A	0.398375024	GSM1855073	-0.073933868	--	--
TCGA-62-A46O-01A	0.557579973	GSM1855074	-0.014383794	--	--
TCGA-62-A46P-01A	-0.283281359	GSM1855075	-0.153029923	--	--
TCGA-62-A46R-01A	-0.040554447	GSM1855076	-0.220953587	--	--
TCGA-62-A46S-01A	-0.309035383	GSM1855077	0.037693797	--	--
TCGA-62-A46V-01A	-0.110924647	GSM1855078	-0.045913514	--	--
TCGA-62-A46Y-01A	-0.259916025	GSM1855079	0.275458802	--	--
TCGA-62-A470-01A	-0.301984971	GSM1855080	-0.081910399	--	--
TCGA-62-A471-01A	0.574005647	GSM1855081	0.272648818	--	--
TCGA-62-A472-01A	0.089307834	GSM1855082	-0.183964766	--	--
TCGA-64-1676-01A	-0.153012399	GSM1855083	0.126200649	--	--
TCGA-64-1677-01A	0.031978422	GSM1855084	0.00463222	--	--
TCGA-64-1678-01A	0.202131194	GSM1855085	0.241761139	--	--

TCGA-64-1679-01A	0.192639383	GSM1855086	-0.018628528	--	--
TCGA-64-1680-01A	-0.061863859	GSM1855087	-0.121253605	--	--
TCGA-64-1681-01A	-0.123630283	GSM1855088	-0.166910812	--	--
TCGA-64-5774-01A	0.215288861	GSM1855089	0.196007233	--	--
TCGA-64-5775-01A	0.790948297	GSM1855091	-0.024515092	--	--
TCGA-64-5778-01A	-0.109404431	GSM1855092	-0.103496073	--	--
TCGA-64-5779-01A	-0.076496047	GSM1855093	-0.214494127	--	--
TCGA-64-5781-01A	0.248846008	GSM1855094	0.220232649	--	--
TCGA-64-5815-01A	0.189692774	GSM1855095	0.417353263	--	--
TCGA-67-3770-01A	-0.302955141	GSM1855096	-0.104537414	--	--
TCGA-67-3771-01A	0.154186514	GSM1855097	0.126946228	--	--
TCGA-67-3772-01A	-0.121255166	GSM1855098	-0.273572499	--	--
TCGA-67-3773-01A	-0.424433825	GSM1855099	-0.102368933	--	--
TCGA-67-3774-01A	-0.197571853	GSM1855100	0.020287984	--	--
TCGA-67-6215-01A	-0.12329505	GSM1855101	0.039718534	--	--
TCGA-67-6216-01A	-0.073938503	GSM1855102	-0.222851635	--	--
TCGA-67-6217-01A	-0.237845689	GSM1855103	0.063468428	--	--
TCGA-69-7760-01A	0.327923678	GSM1855104	0.245325293	--	--
TCGA-69-7761-01A	0.305213398	GSM1855105	-0.351137013	--	--
TCGA-69-7763-01A	-0.261269188	GSM1855106	-0.007155411	--	--
TCGA-69-7764-01A	-0.184417988	GSM1855107	0.213389831	--	--
TCGA-69-7765-01A	-0.140059258	GSM1855108	0.346376793	--	--
TCGA-69-7973-01A	-0.009679672	GSM1855109	0.269712416	--	--
TCGA-69-7974-01A	0.238765007	GSM1855110	-0.287621178	--	--
TCGA-69-7978-01A	0.187214849	GSM1855111	-0.262097358	--	--
TCGA-69-7979-01A	0.060240705	GSM1855112	-0.310774693	--	--
TCGA-69-7980-01A	0.269441578	GSM1855113	-0.433331069	--	--
TCGA-69-8253-01A	-0.040000819	GSM1855114	0.284214066	--	--
TCGA-69-8254-01A	-0.31004166	GSM1855116	0.138654248	--	--
TCGA-69-8255-01A	0.240364196	GSM1855117	0.596134859	--	--
TCGA-69-8453-01A	-0.080654536	GSM1855118	0.330074561	--	--
TCGA-69-A59K-01A	-0.161519685	GSM1855119	0.061175078	--	--
TCGA-71-6725-01A	0.096675527	GSM1855120	0.521322772	--	--
TCGA-71-8520-01A	0.13184647	GSM1855121	-0.156390953	--	--
TCGA-73-4658-01A	-0.087675019	GSM1855122	0.029459432	--	--
TCGA-73-4659-01A	-0.048048882	GSM1855123	-0.289812197	--	--
TCGA-73-4662-01A	-0.223014984	GSM1855124	0.113138576	--	--
TCGA-73-4666-01A	0.174984809	GSM1855125	0.265312398	--	--
TCGA-73-4668-01A	0.229749725	GSM1855126	0.047283245	--	--
TCGA-73-4670-01A	0.377525877	GSM1855127	-0.371421086	--	--
TCGA-73-4675-01A	0.005439955	GSM1855128	0.074578678	--	--
TCGA-73-4676-01A	0.038341137	GSM1855129	-0.22516241	--	--
TCGA-73-4677-01A	-0.176290572	GSM1855131	0.133021248	--	--
TCGA-73-7498-01A	-0.335475142	GSM1855132	-0.196699533	--	--

TCGA-73-7499-01A	0.021015531	GSM1855133	-0.37734567	--	--
TCGA-73-A9RS-01A	0.426083711	GSM1855134	-0.395406115	--	--
TCGA-75-5125-01A	0.130216778	GSM1855135	0.160025072	--	--
TCGA-75-5146-01A	-0.526095203	GSM1855136	-0.046363539	--	--
TCGA-75-5147-01A	0.034559551	GSM1855137	0.083715751	--	--
TCGA-75-6206-01A	-0.21764002	GSM1855138	0.012830702	--	--
TCGA-75-6212-01A	-0.315347208	GSM1855140	-0.072210241	--	--
TCGA-75-6214-01A	0.544709452	GSM1855141	-0.302557474	--	--
TCGA-75-7025-01A	-0.195884577	GSM1855142	0.163163435	--	--
TCGA-75-7027-01A	0.36925912	GSM1855143	-0.272892827	--	--
TCGA-78-7143-01A	0.042202024	GSM1855144	-0.091383942	--	--
TCGA-78-7145-01A	0.269363836	GSM1855145	-0.077499166	--	--
TCGA-78-7146-01A	0.530989577	GSM1855146	0.071625622	--	--
TCGA-78-7147-01A	-0.034378677	GSM1855147	-0.011578948	--	--
TCGA-78-7148-01A	0.043835982	GSM1855149	-0.333345966	--	--
TCGA-78-7149-01A	-0.272107591	GSM1855150	0.024855811	--	--
TCGA-78-7150-01A	0.608135999	GSM1855151	-0.124415461	--	--
TCGA-78-7152-01A	-0.270863675	GSM1855152	-0.268510025	--	--
TCGA-78-7153-01A	-0.328037967	GSM1855153	0.076064051	--	--
TCGA-78-7154-01A	0.124118638	GSM1855156	0.141512527	--	--
TCGA-78-7155-01A	0.130080281	GSM1855157	-0.176312331	--	--
TCGA-78-7156-01A	-0.175049629	GSM1855158	-0.199303333	--	--
TCGA-78-7158-01A	-0.19086714	GSM1855159	-0.052464318	--	--
TCGA-78-7159-01A	-0.003292103	GSM1855160	0.197756057	--	--
TCGA-78-7160-01A	-0.142868114	GSM1855161	-0.012373157	--	--
TCGA-78-7161-01A	-0.079933918	GSM1855162	-0.46768401	--	--
TCGA-78-7162-01A	-0.359661312	GSM1855163	0.069799318	--	--
TCGA-78-7163-01A	0.028952424	GSM1855164	0.257763245	--	--
TCGA-78-7166-01A	0.146010817	GSM1855165	-0.190218271	--	--
TCGA-78-7167-01A	-0.081780003	GSM1855166	0.174643261	--	--
TCGA-78-7220-01A	0.341390034	GSM1855168	-0.036880128	--	--
TCGA-78-7535-01A	0.04411044	GSM1855169	-0.661620507	--	--
TCGA-78-7536-01A	0.258691844	GSM1855170	-0.113673808	--	--
TCGA-78-7537-01A	-0.295431733	GSM1855171	0.347887088	--	--
TCGA-78-7539-01A	-0.069998344	GSM1855172	-0.122557763	--	--
TCGA-78-7540-01A	0.284703045	GSM1855173	-0.00501178	--	--
TCGA-78-7542-01A	0.439767424	GSM1855174	-0.058868223	--	--
TCGA-78-7633-01A	-0.128221345	GSM1855175	-0.120420638	--	--
TCGA-78-8640-01A	0.185091633	GSM1855176	0.008051948	--	--
TCGA-78-8648-01A	-0.284332848	GSM1855177	-0.28702102	--	--
TCGA-78-8655-01A	0.05524047	GSM1855178	0.222053268	--	--
TCGA-78-8660-01A	0.236977672	GSM1855179	0.286940215	--	--
TCGA-78-8662-01A	0.31362219	GSM1855180	-0.128169842	--	--
TCGA-80-5608-01A	-0.127070317	GSM1855181	0.163186957	--	--

TCGA-80-5611-01A	0.113497949	GSM1855182	-0.183669161	--	--
TCGA-83-5908-01A	0.260831142	GSM1855183	-0.272870823	--	--
TCGA-86-6562-01A	0.000760517	GSM1855184	-0.236746862	--	--
TCGA-86-6851-01A	0.023676797	GSM1855185	0.236080494	--	--
TCGA-86-7701-01A	-0.097894621	GSM1855187	-0.034371836	--	--
TCGA-86-7711-01A	0.348333097	GSM1855188	-0.194808141	--	--
TCGA-86-7713-01A	0.101364994	GSM1855189	-0.160846195	--	--
TCGA-86-7714-01A	-0.230253426	GSM1855190	0.210107153	--	--
TCGA-86-7953-01A	0.048187675	GSM1855192	-0.02592457	--	--
TCGA-86-7954-01A	0.024162639	GSM1855193	-0.007977782	--	--
TCGA-86-7955-01A	0.43569358	GSM1855195	0.169963478	--	--
TCGA-86-8054-01A	0.150761286	GSM1855196	0.219914449	--	--
TCGA-86-8055-01A	0.046512065	GSM1855198	0.51400982	--	--
TCGA-86-8056-01A	-0.295482339	GSM1855199	0.732385853	--	--
TCGA-86-8073-01A	-0.113588309	GSM1855200	0.018225829	--	--
TCGA-86-8074-01A	0.038961521	GSM1855202	-0.214381813	--	--
TCGA-86-8075-01A	0.174132933	GSM1855203	0.202816812	--	--
TCGA-86-8076-01A	-0.246014263	GSM1855204	0.229047874	--	--
TCGA-86-8278-01A	-0.016408013	GSM1855206	0.395141279	--	--
TCGA-86-8279-01A	-0.003796954	GSM1855207	0.737272591	--	--
TCGA-86-8280-01A	-0.339514253	GSM1855208	-0.044425201	--	--
TCGA-86-8358-01A	0.045326866	GSM1855209	-0.222932693	--	--
TCGA-86-8359-01A	-0.061209416	GSM1855210	-0.445316626	--	--
TCGA-86-8585-01A	0.127604998	GSM1855211	0.11355511	--	--
TCGA-86-8668-01A	-0.1111768	GSM1855212	-0.431268349	--	--
TCGA-86-8669-01A	-0.175289401	GSM1855213	-0.019010739	--	--
TCGA-86-8671-01A	-0.439055571	GSM1855214	-0.535301028	--	--
TCGA-86-8672-01A	0.164178567	GSM1855215	-0.167745196	--	--
TCGA-86-8673-01A	0.078757504	GSM1855217	-0.409956885	--	--
TCGA-86-8674-01A	-0.0990842	GSM1855218	-0.346772909	--	--
TCGA-86-A456-01A	-0.126680136	GSM1855219	0.291016765	--	--
TCGA-86-A4D0-01A	0.09685456	GSM1855220	0.39127757	--	--
TCGA-86-A4JF-01A	0.0074605	GSM1855221	-0.413866271	--	--
TCGA-86-A4P7-01A	-0.163630845	GSM1855222	-0.290617063	--	--
TCGA-86-A4P8-01A	-0.647256055	GSM1855223	-0.276919438	--	--
TCGA-91-6828-01A	-0.059147626	GSM1855224	0.438175362	--	--
TCGA-91-6829-01A	0.23580679	GSM1855225	0.744786592	--	--
TCGA-91-6830-01A	-0.011462946	GSM1855226	-0.134970885	--	--
TCGA-91-6831-01A	-0.033903638	GSM1855228	0.086698492	--	--
TCGA-91-6835-01A	-0.071358562	GSM1855229	-0.540765264	--	--
TCGA-91-6836-01A	0.205892303	GSM1855230	0.081404362	--	--
TCGA-91-6840-01A	0.009467321	GSM1855231	-0.120258383	--	--
TCGA-91-6847-01A	0.369364533	GSM1855232	0.618590691	--	--
TCGA-91-6848-01A	0.305209542	GSM1855234	0.197480513	--	--

TCGA-91-6849-01A	-0.163327888	GSM1855235	0.11228141	--	--
TCGA-91-7771-01A	-0.167430856	GSM1855236	-0.363697879	--	--
TCGA-91-8496-01A	-0.617369277	GSM1855237	0.589781294	--	--
TCGA-91-8497-01A	-0.433529302	GSM1855238	0.005565554	--	--
TCGA-91-8499-01A	0.270528536	--	--	--	--
TCGA-91-A4BC-01A	-0.082175488	--	--	--	--
TCGA-91-A4BD-01A	-0.317878747	--	--	--	--
TCGA-93-7347-01A	-0.497441464	--	--	--	--
TCGA-93-7348-01A	-0.281781746	--	--	--	--
TCGA-93-8067-01A	0.268167344	--	--	--	--
TCGA-93-A4JN-01A	0.145163832	--	--	--	--
TCGA-93-A4JO-01A	-0.313344225	--	--	--	--
TCGA-93-A4JP-01A	-0.34224728	--	--	--	--
TCGA-93-A4JQ-01A	0.074070687	--	--	--	--
TCGA-95-7039-01A	-0.051169771	--	--	--	--
TCGA-95-7043-01A	0.013951623	--	--	--	--
TCGA-95-7562-01A	0.084388974	--	--	--	--
TCGA-95-7567-01A	0.197218739	--	--	--	--
TCGA-95-7944-01A	-0.015382107	--	--	--	--
TCGA-95-7947-01A	0.018716129	--	--	--	--
TCGA-95-7948-01A	-0.125448373	--	--	--	--
TCGA-95-8039-01A	-0.074886685	--	--	--	--
TCGA-95-8494-01A	0.306088955	--	--	--	--
TCGA-95-A4VK-01A	0.212554755	--	--	--	--
TCGA-95-A4VN-01A	0.022497477	--	--	--	--
TCGA-95-A4VP-01A	-0.12313901	--	--	--	--
TCGA-97-7546-01A	-0.207274094	--	--	--	--
TCGA-97-7547-01A	-0.282770851	--	--	--	--
TCGA-97-7552-01A	-0.302762066	--	--	--	--
TCGA-97-7553-01A	-0.303431164	--	--	--	--
TCGA-97-7554-01A	0.077572044	--	--	--	--
TCGA-97-7937-01A	0.068608754	--	--	--	--
TCGA-97-7938-01A	-0.240368879	--	--	--	--
TCGA-97-7941-01A	-0.106884039	--	--	--	--
TCGA-97-8171-01A	-0.019389821	--	--	--	--
TCGA-97-8172-01A	-0.433828378	--	--	--	--
TCGA-97-8174-01A	-0.330141902	--	--	--	--
TCGA-97-8175-01A	0.092898437	--	--	--	--
TCGA-97-8176-01A	0.361810784	--	--	--	--
TCGA-97-8177-01A	-0.134462198	--	--	--	--
TCGA-97-8179-01A	-0.067400192	--	--	--	--
TCGA-97-8547-01A	-0.010467891	--	--	--	--
TCGA-97-8552-01A	-0.46932124	--	--	--	--
TCGA-97-A4LX-01A	-0.237536401	--	--	--	--

TCGA-97-A4M0-01A	-0.382886237	--	--	--	--
TCGA-97-A4M1-01A	-0.558397462	--	--	--	--
TCGA-97-A4M2-01A	-0.473395679	--	--	--	--
TCGA-97-A4M3-01A	-0.301439	--	--	--	--
TCGA-97-A4M5-01A	-0.240953293	--	--	--	--
TCGA-97-A4M6-01A	-0.240675324	--	--	--	--
TCGA-97-A4M7-01A	-0.216885664	--	--	--	--
TCGA-99-7458-01A	-0.337322239	--	--	--	--
TCGA-99-8025-01A	0.092772346	--	--	--	--
TCGA-99-8028-01A	-0.319301033	--	--	--	--
TCGA-99-8032-01A	-0.075581554	--	--	--	--
TCGA-99-8033-01A	0.339860238	--	--	--	--
TCGA-99-AA5R-01A	-0.568889179	--	--	--	--
TCGA-J2-8192-01A	-0.161522689	--	--	--	--
TCGA-J2-8194-01A	-0.115004029	--	--	--	--
TCGA-J2-A4AD-01A	0.360046267	--	--	--	--
TCGA-J2-A4AE-01A	-0.334805734	--	--	--	--
TCGA-J2-A4AG-01A	-0.18934258	--	--	--	--
TCGA-L4-A4E5-01A	0.035684787	--	--	--	--
TCGA-L4-A4E6-01A	-0.464778058	--	--	--	--
TCGA-L9-A443-01A	-0.1841722	--	--	--	--
TCGA-L9-A444-01A	-0.113482491	--	--	--	--
TCGA-L9-A50W-01A	-0.075870513	--	--	--	--
TCGA-L9-A5IP-01A	0.4268111	--	--	--	--
TCGA-L9-A743-01A	-0.119336008	--	--	--	--
TCGA-L9-A7SV-01A	0.002163842	--	--	--	--
TCGA-L9-A8F4-01A	0.095344448	--	--	--	--
TCGA-MN-A4N1-01A	0.149721967	--	--	--	--
TCGA-MN-A4N4-01A	0.272388949	--	--	--	--
TCGA-MN-A4N5-01A	-0.023522802	--	--	--	--
TCGA-MP-A4SV-01A	-0.012969916	--	--	--	--
TCGA-MP-A4SW-01A	0.050061353	--	--	--	--
TCGA-MP-A4SY-01A	0.043058899	--	--	--	--
TCGA-MP-A4T4-01A	0.015344838	--	--	--	--
TCGA-MP-A4T6-01A	-0.153993269	--	--	--	--
TCGA-MP-A4T7-01A	0.175482534	--	--	--	--
TCGA-MP-A4T8-01A	0.276941442	--	--	--	--
TCGA-MP-A4T9-01A	-0.130874432	--	--	--	--
TCGA-MP-A4TA-01A	0.630820214	--	--	--	--
TCGA-MP-A4TC-01A	0.133583334	--	--	--	--
TCGA-MP-A4TD-01A	-0.347641148	--	--	--	--
TCGA-MP-A4TE-01A	0.180924703	--	--	--	--
TCGA-MP-A4TF-01A	0.418532071	--	--	--	--
TCGA-MP-A4TH-01A	-0.500517898	--	--	--	--

TCGA-MP-A4TI-01A	0.225207298	--	--	--	--
TCGA-MP-A4TJ-01A	-0.204580072	--	--	--	--
TCGA-MP-A4TK-01A	0.184144378	--	--	--	--
TCGA-MP-A5C7-01A	-0.215697725	--	--	--	--
TCGA-NJ-A4YF-01A	0.065645624	--	--	--	--
TCGA-NJ-A4YG-01A	-0.148235988	--	--	--	--
TCGA-NJ-A4YI-01A	-0.088971819	--	--	--	--
TCGA-NJ-A4YP-01A	-0.049853043	--	--	--	--
TCGA-NJ-A4YQ-01A	-0.086985635	--	--	--	--
TCGA-NJ-A55A-01A	-0.480893244	--	--	--	--
TCGA-NJ-A55O-01A	-0.294762072	--	--	--	--
TCGA-NJ-A55R-01A	-0.172306632	--	--	--	--
TCGA-NJ-A7XG-01A	-0.147151498	--	--	--	--
TCGA-O1-A52J-01A	-0.126513773	--	--	--	--
TCGA-S2-AA1A-01A	-0.52746969	--	--	--	--

GSE31210	TRLS	GSE30219	TRLS	GSE3141	TRLS
GSM773540	0.203493921	GSM1465989	-0.037974231	GSM70127	-0.046187373
GSM773541	0.427770766	GSM748053	-0.248114955	GSM70129	0.333084128
GSM773542	0.257891486	GSM748054	0.387378718	GSM70130	-0.038475845
GSM773543	0.069672591	GSM748055	0.034734504	GSM70131	0.094372801
GSM773544	0.136950677	GSM748057	-0.257494268	GSM70136	-0.219325084
GSM773545	-0.002581726	GSM748058	-0.284915807	GSM70138	-0.001345693
GSM773546	0.325421129	GSM748064	-0.211729557	GSM70140	-0.066097384
GSM773547	-0.055580189	GSM748068	0.205732676	GSM70144	-0.205581495
GSM773548	0.049579784	GSM748071	-0.089648997	GSM70145	0.083324954
GSM773549	-0.089060413	GSM748075	0.073271482	GSM70148	-0.19046538
GSM773550	-0.157685872	GSM748077	0.31003622	GSM70150	-0.230003105
GSM773551	-0.149063081	GSM748078	-0.457173146	GSM70151	0.092976778
GSM773552	0.873581767	GSM748079	0.054597163	GSM70153	-0.153078937
GSM773553	0.166372818	GSM748080	0.205210453	GSM70154	-0.118212139
GSM773554	0.459374608	GSM748081	0.075196129	GSM70155	0.201059216
GSM773555	0.261989351	GSM748083	0.081346104	GSM70156	0.104420163
GSM773556	0.344428502	GSM748085	-0.347761311	GSM70159	0.254079482
GSM773557	0.301386846	GSM748086	-0.368314574	GSM70161	-0.050550701
GSM773558	0.31040518	GSM748087	-0.290814059	GSM70163	-0.009128033
GSM773559	0.29564604	GSM748089	0.103610883	GSM70164	0.013107426
GSM773560	0.228326798	GSM748090	-0.213591319	GSM70169	0.030298175
GSM773561	-0.211335459	GSM748091	0.201062884	GSM70170	-0.149255126
GSM773562	0.387005674	GSM748092	0.172214962	GSM70171	-0.012024674
GSM773563	0.216894467	GSM748094	-0.046853687	GSM70172	-0.18953057
GSM773564	-0.078490083	GSM748100	0.066174533	GSM70173	0.024750668
GSM773565	0.307661374	GSM748101	-0.523113355	GSM70174	0.053582997
GSM773566	-0.118386988	GSM748102	0.023606756	GSM70175	-0.134725831
GSM773567	-0.067301885	GSM748103	-0.312255843	GSM70177	-0.041225647

GSM773568	0.194220019	GSM748104	-0.180147135	GSM70179	-0.097392467
GSM773569	-0.352835985	GSM748108	0.097219043	GSM70181	-0.271888443
GSM773570	-0.236322971	GSM748111	0.080101135	GSM70183	-0.407840802
GSM773571	0.195074325	GSM748113	0.283304224	GSM70184	0.049517825
GSM773572	0.058550852	GSM748116	0.401357915	GSM70190	-0.051029932
GSM773573	0.476382037	GSM748121	-0.156765962	GSM70193	0.027392716
GSM773574	0.200127104	GSM748122	0.06325256	GSM70194	-0.24710058
GSM773575	0.41508817	GSM748123	0.077532543	GSM70195	0.051969309
GSM773576	-0.20508319	GSM748124	-0.106462595	GSM70196	-0.048886187
GSM773577	-0.164297959	GSM748125	0.060855965	GSM70197	0.313179065
GSM773578	0.065553007	GSM748126	-0.275146611	GSM70199	0.00952464
GSM773579	0.067080074	GSM748127	0.259577885	GSM70201	-0.021233574
GSM773580	-0.139002228	GSM748128	-0.276759856	GSM70203	-0.231289434
GSM773581	0.436929944	GSM748130	-0.176714362	GSM70204	-0.06827987
GSM773582	0.11592334	GSM748131	-0.007463431	GSM70208	0.0151544
GSM773583	-0.01770394	GSM748135	-0.17528365	GSM70209	-0.10556105
GSM773584	0.155886885	GSM748137	-0.239277217	GSM70214	-0.06048538
GSM773585	0.236355545	GSM748138	0.319877334	GSM70218	0.203578858
GSM773586	0.582911458	GSM748139	0.273697116	GSM70219	-0.013751217
GSM773587	0.116097819	GSM748140	0.204078698	GSM70220	0.089626858
GSM773588	-0.207077155	GSM748142	-0.165509492	GSM70222	0.019497304
GSM773589	0.418141209	GSM748143	-0.003734016	GSM70223	0.262840817
GSM773590	0.033424349	GSM748146	0.060249727	GSM70224	0.003723675
GSM773591	0.262326179	GSM748147	-0.352840962	GSM70225	0.056729124
GSM773592	0.196707844	GSM748150	-0.32521666	GSM70226	-0.294571372
GSM773593	-0.028742903	GSM748152	-0.27727983	GSM70227	0.436210982
GSM773594	-0.192287371	GSM748157	0.011825359	GSM70229	0.283990725
GSM773595	-0.24648885	GSM748161	-0.378772693	GSM70233	0.271181742
GSM773596	-0.127944839	GSM748162	-0.028153625	GSM70234	0.311409004
GSM773597	0.265013764	GSM748164	-0.227610715	GSM70235	0.083939493
GSM773598	0.118147185	GSM748166	-0.081532102	--	--
GSM773599	0.339466614	GSM748167	-0.417303728	--	--
GSM773600	0.186364608	GSM748169	-0.373081757	--	--
GSM773601	0.800620174	GSM748170	0.14793288	--	--
GSM773602	-0.299635252	GSM748171	-0.062077465	--	--
GSM773603	0.320820612	GSM748173	0.096956649	--	--
GSM773604	0.424965772	GSM748175	0.032111999	--	--
GSM773605	-0.134019909	GSM748179	0.766816018	--	--
GSM773606	-0.18389066	GSM748180	-0.232062367	--	--
GSM773607	-0.140208856	GSM748181	-0.355331162	--	--
GSM773608	-0.324217886	GSM748185	0.563016858	--	--
GSM773609	-0.296260111	GSM748186	0.529823845	--	--
GSM773610	-0.121008257	GSM748187	0.075855408	--	--
GSM773611	-0.105199004	GSM748188	-0.070908249	--	--

GSM773612	-0.1837821	GSM748189	0.31979168	--	--
GSM773613	0.283588006	GSM748239	0.830816203	--	--
GSM773614	0.05732517	GSM748240	0.241993341	--	--
GSM773615	0.277567816	GSM748242	0.195865768	--	--
GSM773616	0.721133478	GSM748243	0.033864274	--	--
GSM773617	-0.039425711	GSM748264	-0.119507196	--	--
GSM773618	-0.222588377	GSM748271	0.26847896	--	--
GSM773619	0.043987008	GSM748276	0.55382021	--	--
GSM773620	-0.335727734	GSM748277	-0.166514043	--	--
GSM773621	0.573916766	GSM748278	-0.061374555	--	--
GSM773622	-0.264278195	GSM748279	0.13833948	--	--
GSM773623	-0.349706439	--	--	--	--
GSM773624	-0.421283369	--	--	--	--
GSM773625	-0.082052855	--	--	--	--
GSM773626	0.11290302	--	--	--	--
GSM773627	-0.056044713	--	--	--	--
GSM773628	0.3611574	--	--	--	--
GSM773629	-0.250423903	--	--	--	--
GSM773630	0.332852793	--	--	--	--
GSM773631	-0.146727529	--	--	--	--
GSM773632	-0.059230623	--	--	--	--
GSM773633	0.107118923	--	--	--	--
GSM773634	-0.237257944	--	--	--	--
GSM773635	-0.472240499	--	--	--	--
GSM773636	0.288249874	--	--	--	--
GSM773637	-0.06211162	--	--	--	--
GSM773638	-0.364055912	--	--	--	--
GSM773639	-0.051376501	--	--	--	--
GSM773640	0.124625145	--	--	--	--
GSM773641	-0.095692874	--	--	--	--
GSM773642	-0.089491979	--	--	--	--
GSM773643	0.253584392	--	--	--	--
GSM773644	-0.018074537	--	--	--	--
GSM773645	0.195454596	--	--	--	--
GSM773646	-0.094331298	--	--	--	--
GSM773647	0.089902096	--	--	--	--
GSM773648	0.031343187	--	--	--	--
GSM773649	0.181019691	--	--	--	--
GSM773650	0.113132774	--	--	--	--
GSM773651	0.315150798	--	--	--	--
GSM773652	-0.281841109	--	--	--	--
GSM773653	-0.157559185	--	--	--	--
GSM773654	-0.025738408	--	--	--	--
GSM773655	0.019290643	--	--	--	--

GSM773656	-0.221057421	--	--	--	--
GSM773657	-0.201062908	--	--	--	--
GSM773658	0.30086103	--	--	--	--
GSM773659	0.663911461	--	--	--	--
GSM773660	0.155327305	--	--	--	--
GSM773661	0.379150765	--	--	--	--
GSM773662	-0.235078427	--	--	--	--
GSM773663	0.099828058	--	--	--	--
GSM773664	-0.035695906	--	--	--	--
GSM773665	0.213506061	--	--	--	--
GSM773666	0.173405844	--	--	--	--
GSM773667	0.216276949	--	--	--	--
GSM773668	-0.126260448	--	--	--	--
GSM773669	0.016010576	--	--	--	--
GSM773670	0.068496229	--	--	--	--
GSM773671	-0.398435836	--	--	--	--
GSM773672	-0.093924957	--	--	--	--
GSM773673	0.293398661	--	--	--	--
GSM773674	0.870247487	--	--	--	--
GSM773675	0.155384207	--	--	--	--
GSM773676	-0.078233028	--	--	--	--
GSM773677	0.018116064	--	--	--	--
GSM773678	-0.007147266	--	--	--	--
GSM773679	0.008327494	--	--	--	--
GSM773680	-0.14840278	--	--	--	--
GSM773681	-0.350424067	--	--	--	--
GSM773682	-0.057108284	--	--	--	--
GSM773683	0.331835515	--	--	--	--
GSM773684	-0.10084659	--	--	--	--
GSM773685	-0.011483519	--	--	--	--
GSM773686	-0.26656227	--	--	--	--
GSM773687	-0.06691216	--	--	--	--
GSM773688	-0.064747353	--	--	--	--
GSM773689	-0.420315264	--	--	--	--
GSM773690	-0.304399745	--	--	--	--
GSM773691	-0.199543978	--	--	--	--
GSM773692	-0.292761933	--	--	--	--
GSM773693	-0.025509383	--	--	--	--
GSM773694	-0.115944534	--	--	--	--
GSM773695	-0.288443788	--	--	--	--
GSM773696	-0.108811888	--	--	--	--
GSM773697	0.141337816	--	--	--	--
GSM773698	0.04611755	--	--	--	--
GSM773699	-0.282146518	--	--	--	--

GSM773700	-0.025437499	--	--	--	--
GSM773701	-0.306874131	--	--	--	--
GSM773702	-0.058246926	--	--	--	--
GSM773703	0.315645971	--	--	--	--
GSM773704	-0.208904064	--	--	--	--
GSM773705	-0.171338554	--	--	--	--
GSM773706	-0.327288843	--	--	--	--
GSM773707	-0.097668659	--	--	--	--
GSM773708	-0.03178649	--	--	--	--
GSM773709	-0.248060513	--	--	--	--
GSM773710	-0.246902615	--	--	--	--
GSM773711	-0.265019009	--	--	--	--
GSM773712	-0.133240222	--	--	--	--
GSM773713	0.141131856	--	--	--	--
GSM773714	-0.040487123	--	--	--	--
GSM773715	0.256934232	--	--	--	--
GSM773716	0.145717013	--	--	--	--
GSM773717	0.065728504	--	--	--	--
GSM773718	-0.359216264	--	--	--	--
GSM773719	-0.079935966	--	--	--	--
GSM773720	0.037780049	--	--	--	--
GSM773721	-0.403493968	--	--	--	--
GSM773722	-0.011431408	--	--	--	--
GSM773723	0.195999206	--	--	--	--
GSM773724	-0.092429091	--	--	--	--
GSM773725	-0.043160847	--	--	--	--
GSM773726	-0.083788843	--	--	--	--
GSM773727	0.144899856	--	--	--	--
GSM773728	-0.1111347	--	--	--	--
GSM773729	0.037712412	--	--	--	--
GSM773730	0.227844765	--	--	--	--
GSM773731	-0.190772088	--	--	--	--
GSM773732	-0.199527712	--	--	--	--
GSM773733	-0.104459998	--	--	--	--
GSM773734	-0.234232506	--	--	--	--
GSM773735	-0.277000897	--	--	--	--
GSM773736	-0.142547067	--	--	--	--
GSM773737	-0.11609356	--	--	--	--
GSM773738	-0.062123594	--	--	--	--
GSM773739	-0.263660022	--	--	--	--
GSM773740	0.006328149	--	--	--	--
GSM773741	0.040681041	--	--	--	--
GSM773742	0.305524998	--	--	--	--
GSM773743	-0.135995429	--	--	--	--

GSM773744	-0.298190804	--	--	--	--
GSM773745	-0.111218936	--	--	--	--
GSM773746	-0.120690074	--	--	--	--
GSM773747	-0.360563776	--	--	--	--
GSM773748	-0.569941228	--	--	--	--
GSM773749	0.2823213	--	--	--	--
GSM773750	-0.374289006	--	--	--	--
GSM773751	-0.104185673	--	--	--	--
GSM773752	-0.199600062	--	--	--	--
GSM773753	-0.163597741	--	--	--	--
GSM773754	-0.112788268	--	--	--	--
GSM773755	-0.181831658	--	--	--	--
GSM773756	-0.238544059	--	--	--	--
GSM773757	-0.271010351	--	--	--	--
GSM773758	-0.356206083	--	--	--	--
GSM773759	-0.392407862	--	--	--	--
GSM773760	-0.166791955	--	--	--	--
GSM773761	-0.29344568	--	--	--	--
GSM773762	0.044938083	--	--	--	--
GSM773763	-0.230702589	--	--	--	--
GSM773764	0.091202376	--	--	--	--
GSM773765	-0.34898403	--	--	--	--
