# Supplementary material for: Single-cell RNA sequencing integrated with bulk RNA sequencing analysis identifies a tumor immune microenvironment-related lncRNA signature in lung adenocarcinoma
Source: BMC Biol. 2024 Mar 22;22:69. doi: 10.1186/s12915-024-01866-5 (PMC10960411; doi:10.1186/s12915-024-01866-5)
Supplement: Supplementary file 14 — Additional file 14: Table S8. Multivariate Cox regression of TRLS regarding to OS. [file 12915_2024_1866_MOESM14_ESM.pdf]

Table S8. Multivariate Cox regression of TRLS regarding to OS.

Cohort	Clinical Characteristics	HR	HR.95L	HR.95H	P-value
**TCGA-LUAD**	**TRLS (Risk score)**	**7.467045**	**3.628351**	**15.36697**	**4.76E-08**
TCGA-LUAD	Age (>65 vs. <=65)	1.265882	0.891891	1.796697	0.186968
TCGA-LUAD	Gender (Male vs. Female)	0.992008	0.704902	1.396051	0.963287
TCGA-LUAD	Stage (III-IV vs. I-II)	1.52601	0.664199	3.506035	0.319315
TCGA-LUAD	T stage (T3-T4 vs. T1-T2)	1.75069	1.072971	2.856473	0.024966
TCGA-LUAD	N stage (N2-N3 vs. N0-N1)	1.890096	1.264955	2.824182	0.00189
TCGA-LUAD	M stage (M1 vs. M0)	1.283388	0.527437	3.122806	0.582364
**GSE30219**	**TRLS (Risk score)**	**47.69727788**	**11.66968014**	**194.9522429**	**7.43E-08**
GSE30219	Age (>65 vs. <=65)	2.669110948	1.391411582	5.120090523	0.003138849
GSE30219	Gender (Male vs. Female)	0.785037388	0.357892029	1.721982192	0.545914577
GSE30219	T stage (T3-T4 vs. T1-T2)	0.196397327	0.017500419	2.204056368	0.187053284
GSE30219	N Stage (N1 vs. N0)	0.497498384	0.08096883	3.056789161	0.451025812
**GSE31210**	**TRLS (Risk score)**	**9.051775**	**2.508588**	**32.66165**	**0.000766**
GSE31210	Age (>65 vs. <=65)	4.2514	2.005999	9.010178	0.000159
GSE31210	Gender (Male vs. Female)	0.937433	0.339494	2.5885	0.900779
GSE31210	Stage (II vs. I)	4.152957	2.012823	8.568588	0.000117
GSE31210	Smoke (Yes vs. No)	1.057295	0.380189	2.940307	0.914977
GSE31210	EGFR (Yes vs. No)	0.762918	0.35264	1.650535	0.491908
GSE31210	KRAS (Yes vs. No)	0.452934	0.130829	1.568066	0.211301
GSE31210	ALK (Yes vs. No)	1.356373	0.288459	6.377851	0.699549
**GSE50081**	**TRLS (Risk score)**	**7.704926**	**1.979374**	**29.99226**	**0.003234**
GSE50081	Age (>65 vs. <=65)	1.386981	0.690251	2.786983	0.358203
GSE50081	Gender (Male vs. Female)	1.667542	0.88306	3.148933	0.1149

GSE50081	Stage (II vs. I)	2.0483	1.068326	3.927206	0.030852
GSE50081	T stage (T3-T4 vs. T1-T2)	3.326755	0.669704	16.52566	0.141632
GSE50081	N Stage (N1 vs. N0)	1.558513	0.84828	2.863397	0.152781
GSE50081	Smoke (Yes vs. No)	0.948476	0.39599	2.271788	0.905513
**GSE72094**	**TRLS (Risk score)**	**7.92721363**	**3.230854782**	**19.45018275**	**6.16E-06**
GSE72094	Age (>65 vs. <=65)	1.135667755	0.692246387	1.863124567	0.614472912
GSE72094	Gender (Male vs. Female)	2.195570335	1.399220256	3.445153883	0.000623171
GSE72094	Stage (II vs. I)	3.485449976	2.194599586	5.535570867	1.22E-07
GSE72094	EGFR (Yes vs. No)	0.372920081	0.113183581	1.228706367	0.104930098
GSE72094	KRAS (Yes vs. No)	1.09203264	0.690248978	1.727688596	0.706804899
GSE72094	TP53 (Yes vs. No)	0.593750723	0.346801475	1.016546774	0.05741424
GSE72094	STK11 (Yes vs. No)	0.555396051	0.28343248	1.088318366	0.086642692
GSE72094	Smoke (Yes vs. No)	1.038127287	0.427795763	2.519212102	0.934069567
